# Structure–Activity Relationships and Biological Evaluation of 7-Substituted Harmine Analogs for Human β-Cell Proliferation

**DOI:** 10.3390/molecules25081983

**Published:** 2020-04-23

**Authors:** Kunal Kumar, Peng Wang, Ethan A. Swartz, Susmita Khamrui, Cody Secor, Michael B. Lazarus, Roberto Sanchez, Andrew F. Stewart, Robert J. DeVita

**Affiliations:** 1 Drug Discovery Institute, Icahn School of Medicine at Mount Sinai, New York, NY 10029, USA; kunal.kumar@mssm.edu (K.K.); Michael.Lazarus@mssm.edu (M.B.L.); Roberto.Sanchez@mssm.edu (R.S.); 2 Department of Pharmacological Sciences, Icahn School of Medicine at Mount Sinai, New York, NY 10029, USA; Susmita.Khamrui@mssm.edu (S.K.); Cody.Secor@mssm.edu (C.S.); 3 Diabetes, Obesity, and Metabolism Institute, Icahn School of Medicine at Mount Sinai, New York, NY 10029, USA; Peng.wang@mssm.edu (P.W.); ethan.swartz@mssm.edu (E.A.S.); Andrew.Stewart@mssm.edu (A.F.S.)

**Keywords:** dual-specificity tyrosine-regulated kinases (DYRKs), harmine, DYRK1A inhibitor, structure–activity relationship study, β-cell proliferation, diabetes

## Abstract

Recently, we have shown that harmine induces β-cell proliferation both in vitro and in vivo, mediated via the DYRK1A-NFAT pathway. We explore structure–activity relationships of the 7-position of harmine for both DYRK1A kinase inhibition and β-cell proliferation based on our related previous structure–activity relationship studies of harmine in the context of diabetes and β-cell specific targeting strategies. 33 harmine analogs of the 7-position substituent were synthesized and evaluated for biological activity. Two novel inhibitors were identified which showed DYRK1A inhibition and human β-cell proliferation capability. The DYRK1A inhibitor, compound **1-2b**, induced β-cell proliferation half that of harmine at three times higher concentration. From these studies we can draw the inference that 7-position modification is limited for further harmine optimization focused on β-cell proliferation and cell-specific targeting approach for diabetes therapeutics.

## 1. Introduction

The dual-specificity tyrosine-phosphorylation-regulated kinase 1A (DYRK1A) is a eukaryotic protein kinase that has been shown to play important roles in biological processes related to various diseases [1,2,3,4] and more recently, as a major regulator of human insulin-producing pancreatic β-cells [5,6,7,8]. Numerous studies have previously reported the development of DYRK1A inhibitor scaffolds for a variety of therapeutic applications including diabetes [2,3,4,5,6,8,9,10,11,12,13,14,15,16,17,18,19,20,21,22,23,24,25,26,27,28,29,30,31,32,33]. Among all the DYRK1A inhibitors, harmine and its analogues (β-carbolines) are the most commonly studied, and remain one of the most potent and orally bioavailable class of inhibitors known to date [2,10].

**Figure 1:**
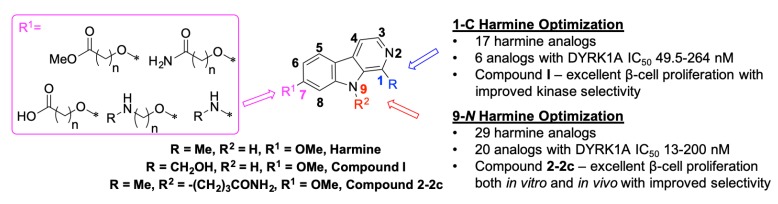
Optimization of the 7-position of harmine.

Recently, our group found that harmine is able to induce human β-cell proliferation and DYRK1A-NFAT pathway as being the major pathway for this cell proliferation [5]. These results have been confirmed in other labs with other DYRK1A inhibitors unrelated to the harmine scaffold, including from our own lab [6,8,34,35,36,37]. Since, modification of harmine has not been explored previously in context of β-cell proliferation, we therefore decided to carry out structure–activity relationship studies of harmine for both DYRK1A inhibition and β-cell proliferation. Based on the known binding pose of harmine, we surmised that there are three positions, 1-methyl,7-methoxy and 9-*N* indole, where rational modifications of harmine can be carried out (Figure 1) without disrupting key DYRK1A binding contacts. Previously, we reported the optimization of the 1-position of harmine which led to two compounds with robust human β-cell proliferation at doses of 3–30 µM and one of those, compound I (R = CH_2_OH), showing improved kinase selectivity as compared to harmine (Figure 1) [38]. Next, we recently reported the optimization of the *N*-9 position to identify **2-2c**, a highly optimized in vivo active harmine based DYRK1A inhibitor (Figure 1) [39]. To extend this systematic medicinal chemistry strategy, we last focused our effort on the harmine 7-position to understand the impact of modifications on DYRK1A inhibition and β-cell proliferation (Figure 1). An additional consideration was to explore the potential for 7-substituents to link to putative targeting molecules for cell specific delivery to the β-cells, without negatively affecting DYRK1A and β-cell proliferative biological activity. One such prototype targeting technology for cells that highly express GLP1-receptor, including β-cells, was reported by DiMarchi and colleagues [40,41]. Herein, we report the results of our 7-position of harmine medicinal chemistry studies. We first determined the effect of these modifications on DYRK1A inhibition, measured by FRET-based LanthaScreen^®^ Eu Kinase Binding Assay [42]. Since the overall goal of the project is to develop DYRK1A inhibitors that can induce β-cells to proliferate, we also assayed potent 7-substituted harmine analogs for human β-cell proliferation by quantifying Ki67 immunolabeling in beta cells [5,39,43,44,45].

## 2. Results and Discussion

### 2.1. Synthesis

**Scheme 1:**
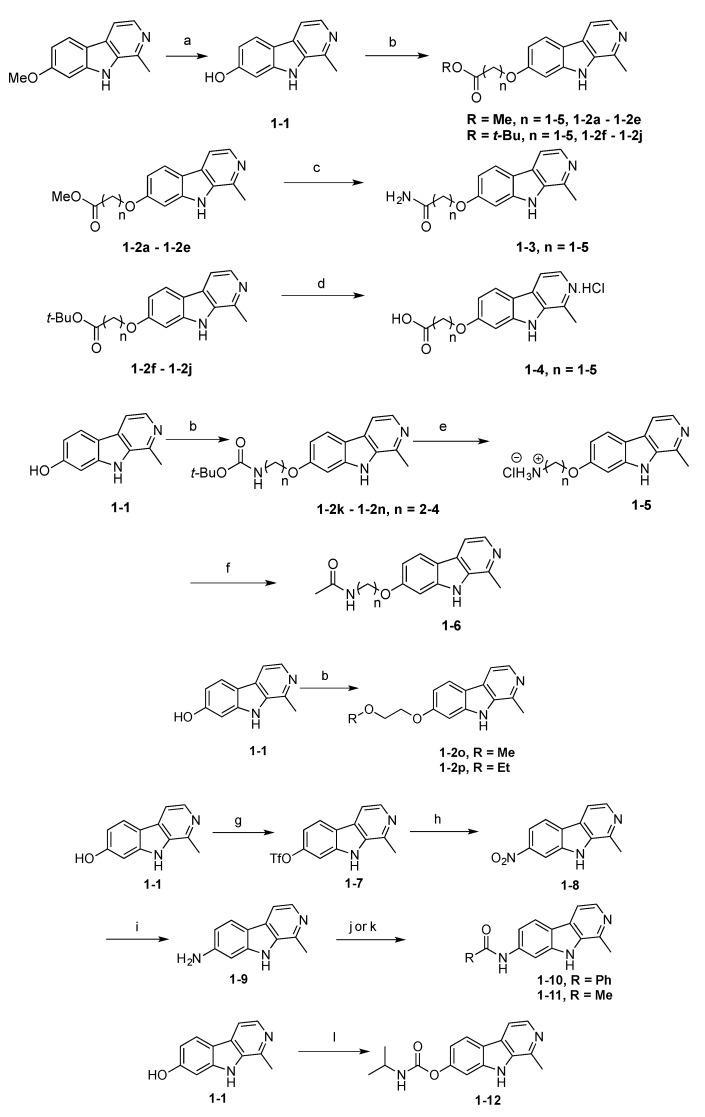
Synthesis of 7-Substituted Harmine Analogs. Reagents and conditions: (**a**) AcOH, 48% HBr, reflux, 12 h, 99%; (**b**) Cs_2_CO_3_ (1.5 eq.), Br(CH_2_)_n_CO_2_R (1.5 eq.), DMF, 50 °C, 12 h, 32–68%; (**c**) 7N NH_3_ in MeOH (20 eq.), 90 °C, 12 h, 84–97%; (**d**) 4N HCl in dioxane, rt, 12 h, 82–98%; (**e**) 4N HCl in dioxane, dioxane, rt, 12 h, 90–99%; (**f**) Acetic anhydride (1 eq.), Et_3_N (2.2eq.), DCM, rt, 12 h, 49–79%; (**g**) Tf_2_O (1.2 eq.), Pyridine, DCM, 0 °C-rt, 12h, 92%; (**h**) NaNO_2_ (2 eq.), Pd_2_(dba)_3_ (5 mol%), BrettPhos (12 mol%), TDA (5 mol%), *t*-BuOH, 150 °C, 24 h, 77%; (**i**) 10% wt Pd on C, N_2_H_4_.H_2_O (20 eq.), MeOH, reflux, 2 h, 98%; (**j**) Benzoyl chloride (1.05 eq.), Et_3_N (2 eq.), THF, 0 °C-rt, 12 h, 75%; (**k**) Acetic anhydride (1.2 eq.), Et_3_N (2 eq.), THF, rt, 24 h, 77%; (**l**) Isopropylisocyanate (1.1 eq.), DMF, rt, 12 h, 32%.

Modification of the harmine 7-position was carried out by several standard reaction sequences outlined in Scheme 1. Harmine underwent demethylation in the presence of HBr/AcOH to give harmol **1-1** in 99% yield [46]. Alkylation of harmol **1-1** with various alkyl bromides in the presence of cesium carbonate as base generated, in 40–68% yield, 7-ether harmine analogs **1-2a**–**1-2n** having a terminal methyl ester, *t*-butyl ester and BOC-protected amino group with carbon chain lengths of 1–5 carbons [46]. Harmine analogs **1-2a**–**1-2e** with terminal methyl esters were refluxed in excess methanolic ammonia solution to provide the corresponding terminal carboxamide analogs **1-3a**–**1-3e** in 84–95% yield. *t*-Butyl ester harmine analogs **1-2f**–**1-2j** were treated with hydrochloric acid at room temperature to give corresponding acids **1-4a**–**1-4e** in excellent yield. Compounds **1-2k**–**1-2n** underwent *N-BOC* deprotection to generate harmine analogs **1-5a**–**1-5d** with terminal amino functional group which then, underwent acylation using acetic anhydride to afford harmine 7-alkoxy acetamide analogs **1-6a**–**1-6d**. Harmol (**1-1**) was also treated with trifluoromethanesulfonic anhydride to form *O-*trifluoromethanesulfonyl harmine analog **1-8** which underwent palladium catalyzed nitration reaction, developed by Buchwald lab [47], followed by nitro group reduction to generate 7-amino harmine analog **1-9** in 77% yield. Derivatization of 7-amino functional group of **1-9** with acetic anhydride and benzoyl chloride in presence of triethyl amine afforded carboxamides **1-10** and **1-11,** respectively. Harmine analogs **1-2o** and **1-2p**, bearing alkyl ether substituents at the 7-positions, were synthesized by standard basic alkylation of harmol **1-1**. Compound **1-12** containing the carbamate functional group at the 7-position was synthesized in 32% yield by treating harmol **1-1** with isopropylisocyanate and trimethylamine [48]. HNMR spectra of synthesized compounds are included in Supplementary Material.

### 2.2. Structure–Activity Relationships of 7-Substituted Harmine Analogs

**Figure 2:**
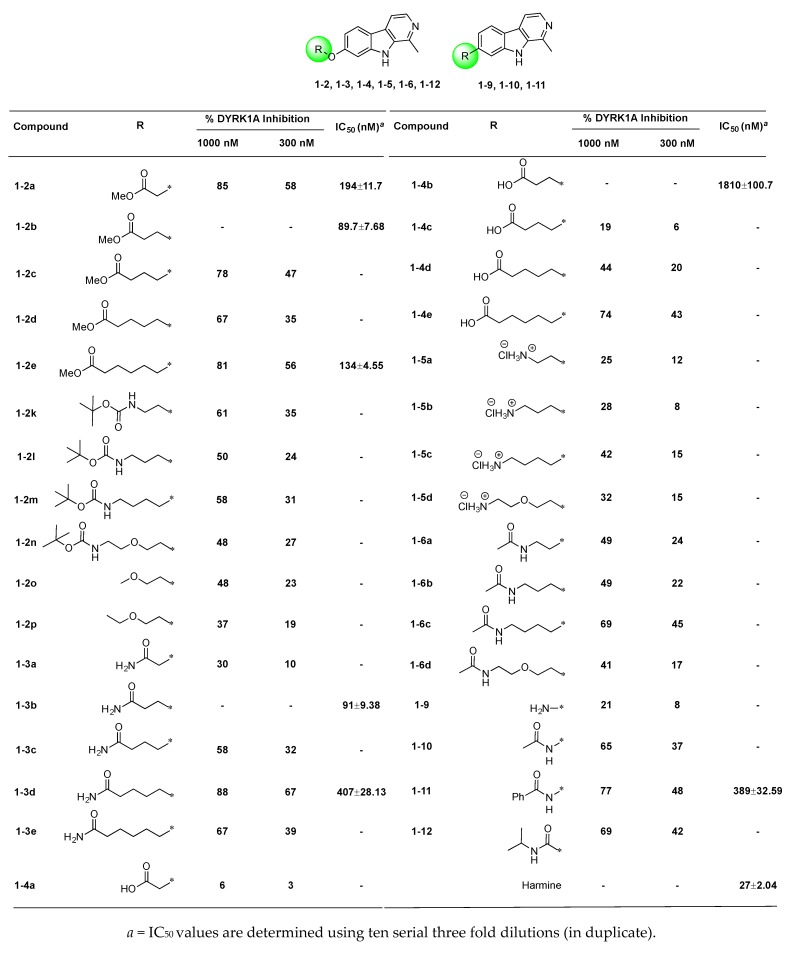
Dual-specificity Tyrosine-phosphorylation-Regulated Kinase A (DYRK1A) inhibition of 7-C harmine analogs *^a^*.

As outlined in Figure 1 and Scheme 1, modification of the harmine 7-position was carried out to identify linkable harmine analogs by first introducing substituents bearing a terminal methyl ester (**1-2a** to **1-2e**), carboxamide (**1-3a** to **1-3e**), carboxylic acid (**1-4a** to **1-4e**) and amino/substituted amino (**1-2k** to **1-2n**, **1-5a** to **1-5d**, and **1-6a** to **1-6d**) functional groups with chain length varying from 1–5 carbons. We first studied the effect of introducing these modifications on DYRK1A inhibition, measured by FRET-based LanthaScreen^®^ Eu Kinase Binding Assay [42]. Among the 33 harmine analogs, only carboxamide and methyl ester functional groups were well tolerated to provide DYRK1A inhibition IC_50_′s less than 100 nM. 7-substituted methyl ester harmine analog (**1-2b**) and related 3-carbon analog (**1-3b**) with ethylene (2-carbon) chain between harmine 7-oxygen and the carboxamide, showed the most potent DYRK1A inhibitory activity of this set of analogs with DYRK1A IC_50_ in the range of 89–90 nM (Figure 2). These compounds are ~3-fold less potent inhibitors of DYRK1A kinase in vitro as compared to harmine itself (27 nM).

**Figure 3:**
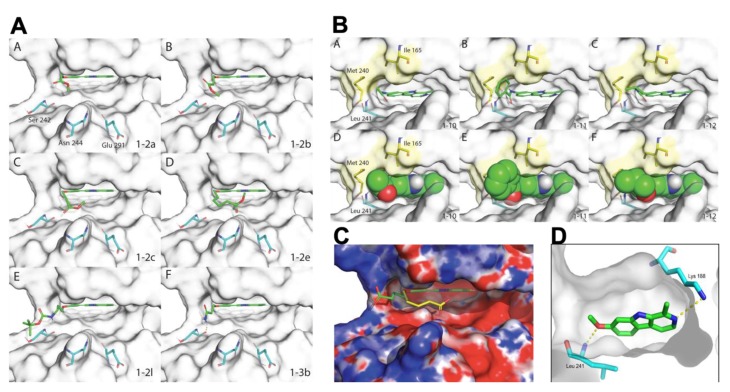
Docking of selected 7-substituted harmine analogs. **A.** Docking of **1-2a** (subpanel A), **1-2b** (subpanel B), **1-2c** (subpanel C), **1-2e** (subpanel D), **1-2l** (subpanel E), **1-3b** (subpanel F). Ligands are shown in green, the protein surface in gray, and selected residues in light blue. **B.** Docking of compounds **1-10** (subpanel A,D)**, 1-11** (subpanel B,E), and **1-12** (subpanel C,F). Subpanel A-C stick model of the ligand docked into the ATP-binding pocket of DYRK1A. Subpanel D–F, space filling models of the same structures shown in A–C. All three compounds are unable to hydrogen bond with the backbone of Leu 241 (used by harmine, panel D) due to the unfavorable orientation of their carbonyl oxygen. The hydrophobic substituents however interact with a hydrophobic cleft formed by the side chain of Ile 165 and Met 240 (shown in yellow). **C.** Docking of 7-substituted Harmine analogs containing carboxylic acid groups. Compound **1-4c** is shown in green and compounds **1-4e** in yellow. **D.** Harmine bound to DYRK1A (PDB 3ANR). The surface of the protein is colored according to the electrostatic potential (red for negative and blue for positive).

Interestingly, other analogs with longer carbon lengths did not provide any improvement for DYRK1A inhibition, a result that did not align with our original docking hypothesis, which indicated that extension into solvent would be possible. Subsequent docking studies of these novel 7-C harmine analogs based on the crystal structure of DYRK1A bound to harmine analog **2-2c** [39] were performed and suggested that the loss of activity with longer substituents at the 7-position could be due to interplay of several factors that diminish interactions with the DYRK1A back bone H-bond at Leu 241. These include steric hindrance, the entropic effects of longer, more flexible side chains, combined with the possibility of partially compensating additional new contacts (Figure 3A, subpanel A–F). The shorter chains (**1-2b** (Figure 3A, subpanel B), **1-3b** (Figure 3A, subpanel F)) are expected to have a reduced entropic effect and therefore, the activity is similar to that of harmine. In the case of carboxamide **1-3b** there is also the potential for a compensatory new contact with Ser 242 (Figure 3A, subpanel F). Several analogs with longer chains are accommodated as a result of their flexibility, which allows them to fold back in the case of **1-2c** (Figure 3A, subpanel C) or expand into unoccupied space, as the case of **1-2l** (Figure 3A, subpanel E) based on this model. These data suggest that analogs that do not contribute new contacts to DYRK1A, result in a loss of potency, likely due to of the increased entropic cost of more flexible 7-position side chains. These docking studies also indicate that the chain in **1-2e** (Figure 3A, subpanel D) folds back to make a hydrogen bond with Asn 244 (one of the crucial contacts made by the 9-*N* harmine carboxamide analog, Figure 3D) partially offsetting this entropic penalty.

It was also observed that carboxylic acid and amino functional groups at the 7-position, regardless of their chain length, did not exhibit DYRK1A inhibition in vitro at the concentrations tested. Docking of the harmine analogs that possess negatively charged substituents, such as carboxylic acid or positively charged protonated amines, indicate that they have a detrimental effect on binding due to the electrostatic environment of the DYRK1A binding pocket, irrespective of the orientation of the chain (Figure 3C). For these substituents the expectation is that, the longer the chain the lower the electrostatic effect (because it can extend out of the negatively charged binding pocket toward solvent), resulting in greater entropic cost. Thus, based on these data and our analysis of subsequent structural docking studies, it is now difficult to expect compounds with a carboxylic acid or amino containing substituents at the harmine 7-position to be potent DYRK1A inhibitors suitable for linkage to cell-specific targeting molecules.

Simple 7-position alkyl ether substitutions, for example, 7-oxo-(methoxyethyl) (**1-2o**) and 7-oxo-(ethoxyethyl) (**1-2p**), were also not favorable for the DYRK1A inhibition. We speculate that these modifications hinder the hydrogen bond from Leu241 backbone N-H with the 7-oxygen atom that is more effectively made with the sterically less demanding 7-OMe group of harmine. Additionally, we also investigated the effect of replacing 7-methoxy group with amino and substituted amino groups. Unfortunately, 7-amino harmine analog **1-9** completely lost its DYRK1A inhibitory activity. Docking results indicate that due to the orientation of the Leu241 carbonyl oxygen, hydrogen bonding with Leu241 residue seems unlikely (Figure 3B, subpanel A–F. DYRK1A inhibitory activity was partially recovered when the 7-amino group was modified to benzamido (**1-11**) group (Figure 3B, subpanel B,E). From modeling studies, it appears that favorable packing of the benzyl ring between the sidechains of Met240 and Ile165 may contribute to the DYRK1A binding of this compound. These two side chains form a small hydrophobic cleft (shown in yellow in Figure 3B, subpanel B,E). The 7-carbamate harmine analog **1-12** was also found to be moderately active for DYRK1A inhibition. The majority of 7-substituted analogs demonstrate that these harmine modifications do not result in DYRK1A inhibitors superior in potency to harmine, though some retain potency in the 90–100 nM range.

### 2.3. Effects of Harmine Analogs on Human β-Cell Proliferation

**Figure 4:**
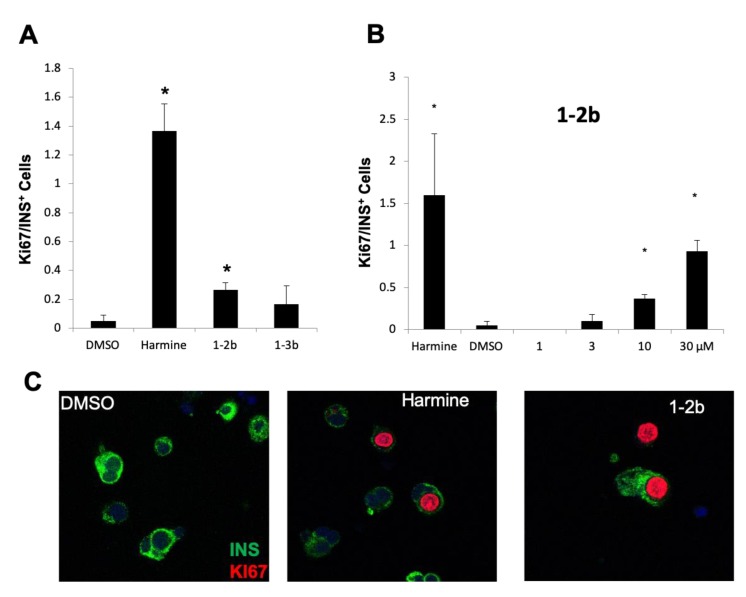
Effects of harmine analogues on human β-cell proliferation. (**A**). Initial screening of harmine analogues on human β-cell proliferation at 10 μM. DMSO was used as a negative control, and harmine was used as a positive control (*n* = 3). (**B**) Dose–response curves for human β-cell proliferation for **1-2b** in human β-cells (*n* = 3 human islet donors for each dose; harmine (10 μM) is the positive control. (**C**) A representative example from (**B**) of Ki67-insulin double-positive cells induced by **1-2b** and harmine. Error bars indicate SEM and * indicates *p* < 0.05. A minimum of 1000 β-cells was counted for each graph. In all relevant panels, error bars indicate SEM and, * indicates *p* < 0.05. A minimum of 1000 β-cells was counted for each graph.

After assessing the effect of these modifications on DYRK1A inhibition, we next explored the structure–activity relationships for in vitro β-cell proliferation activity of the most potent 7-O-substituted DYRK1A inhibitor harmine analogs. Compounds **1-2b** and **1-3b**, with DYRK1A inhibitory activities (IC_50_) of 89 and 91 nM, respectively, were studied as previously reported [5,39,43,44,45], for their ability to induce human β-cell proliferation in vitro as assessed by Ki-67-insulin co-immunolabeling after 4 days, our standard assay protocol [49] (Figure 4A). Both the harmine analogues, **1-2b** and **1-3b** exhibited human β-cell proliferation at 10 μM (Figure 4A). However, the extent of β-cell proliferation was significantly lower than that of harmine itself at that concentration, with analog **1-2b** trending slightly better than **1-3b**. β-cell proliferation dose-response experiments indicated that **1-2b** induced proliferation in a dose-dependent manner. Compound **1-2b** induced proliferation half that of harmine (10 μM) at the highest tested concentration of 30 μM (Figure 4B). This could be attributed to the fact that compound **1-2b** is 4-fold less potent in DYRK1A inhibition as compared to harmine, though it can be inferred that it effectively engages with the target DYRK1A in the live β-cell. Note that the harmine dose-response curve is identical to that reported earlier [5]. Additionally, we can also speculate that slightly lower β-cell proliferation activity of **1-3b** as compared to **1-2B** could be caused by inhibition of kinases which could have anti-proliferative effects. Collectively, these data indicate that modification at 7-position of harmine does provide us with compounds which show real and comparable increases in β-cell proliferation but less than that of harmine itself.

## 3. Materials and Methods

^1^H-NMR were acquired on a Bruker DRX-600 spectrometer at 600 MHz. (Brucker Corporation, Billerica, MA, USA). TLC was performed on silica coated aluminum sheets (thickness 200 µm) or alumina coated (thickness 200 µm) aluminum sheets supplied by Sorbent Technologies (Sorbent Technologies, Inc., Norcross, GA, USA) and column chromatography was carried out on Teledyne ISCO combiflash (Teledyne ISCO, Lincoln, NE, USA) equipped with a variable wavelength detector and a fraction collector using a RediSep Rf high performance silica flash columns by Teledyne ISCO. LCMS/HPLC analysis for purity and HRMS was conducted on an Agilent Technologies G1969A high-resolution API-TOF mass spectrometer attached to an Agilent Technologies 1200 HPLC system (Agilent Technologies, Santa Clara, CA, USA). Samples were ionized by electrospray ionization (ESI) in positive mode. Chromatography was performed on a 2.1 × 150 mm Zorbax 300SB-C18 5-μm column with water containing 0.1% formic acid as solvent A and acetonitrile containing 0.1% formic acid as solvent B at a flow rate of 0.4 mL/min. The gradient program was as follows: 1% B (0−1 min), 1−99% B (1−4 min), and 99% B (4−8 min). The temperature of the column was held at 50 °C for the entire analysis. The chemicals and reagents were purchased from Sigma Aldrich Co. (Sigma Aldrich Corp., St. Louis, MO, USA) Alfa Aesar (Alfa Aesar, Tewksbury, MA, USA), Enamine (Enamine Ltd, Monmouth Jct. NJ, USA), TCI America (TCI America, Seekonk, MA, USA). All solvents were purchased in anhydrous from Acros Organics (Thermo Fisher Scientific, Washington, USA) and used without further purification.

### 3.1. Chemistry

*1-Methyl-9H-pyrido [3,4-b]indol-7-ol* (**1-1**), a solution of harmine (1.0 g, 4.02 mmol) in glacial acetic acid (16 mL) and 48% hydrobromic acid solution (20 mL) was heated at reflux for 10 h. After cooling to room temperature, the mixture was adjusted to pH 8 with a saturated aqueous solution of NaHCO_3_. The yellow slurry was filtered, and the cake was washed with water to afford harmol **1-1** as a white solid (0.91 g, 99%). ^1^H-NMR (600 MHz, *d_6_*-DMSO): δ 10.46 (s, 1H, NH), 8.39 (d, *J* = 6.6 Hz, 1H, ArH3), 8.35 (d, *J* = 6 Hz, 1H, ArH4), 8.26 (d, *J* = 8.4 Hz, 1H, ArH5), 7.00 (s, 1H, ArH8), 6.90 (m,1H, ArH6), 2.93 (s, 3H, CH_3_); MS (ESI) *m/z* 199.08 [M + H]^+^.

General procedure for the synthesis of (**1-2**), a solution of harmalol **1-1** (2.02 mmol) and cesium carbonate (1.5 eq.) in DMF (7 mL) was stirred at 60 °C for 1 h. To this solution was added alkyl bromide (1.5 eq.) and stirred at 50 °C for 12 h. After completion of the reaction confirmed by TLC, the reaction mixture was diluted with water, transferred to separatory funnel and extracted with ethyl acetate (50 mL × 2). The organic layer was washed with water, dried over magnesium sulfate, filtered, evaporated and purified by flash column chromatography to yield the desired product **1-2** as white solid.

*2-(7-Oxy-1-methyl-9-H-b-carbolin)acetic acid methyl ester* (**1-2a**), white solid. Yield 59%. ^1^H-NMR (600 MHz, CD_3_OD): δ 8.07 (d, *J* = 5.4 Hz, 1H, ArH3), 7.96 (d, *J* = 9 Hz, 1H, ArH5), 7.76 (d, *J* = 5.4 Hz, 1H, ArH4), 6.96 (d, *J* = 2.4 Hz, 1H, ArH8), 6.87 (m, 1H, ArH6), 4.79 (s, 2H, CH_2_), 3.80 (s, 3H, CH_3_), 2.73 (s, 3H, CH_3_); HRMS (ESI): *m*/*z* [M + H]^+^ calcd for C_15_H_15_N_2_O_3_^+^: 271.1077, found: 271.1071; Purity >95%.

*3-(7-Oxy-1-methyl-9-H-b-carbolin)propionic acid methyl ester* (**1-2b**), white solid. Yield 44%. ^1^H-NMR (600 MHz, CD_3_OD): δ 8.09 (d, *J* = 5.4 Hz, 1H, ArH3), 7.95 (d, *J* = 8.4 Hz, 1H, ArH5), 7.80 (d, *J* = 5.4 Hz, 1H, ArH4), 6.92 (s, 1H, ArH8), 6.79 (m, 1H, ArH6), 4.84 (t, *J* = 7.2 Hz, 2H, CH_2_), 3.57 (s, 3H, CH_3_), 2.98 (s, 3H, CH_3_), 2.80 (t, *J* = 7.2 Hz, 2H, CH_2_); HRMS (ESI): *m*/*z* [M + H]+ calcd for C_16_H_17_N_2_O_3_^+^: 285.1234, found: 285.1235; Purity >95%.

*4-(7-Oxy-1-methyl-9-H-b-carbolin)butnoic acid methyl ester* (**1-2c**), white solid. Yield 68%. ^1^H-NMR (600 MHz, CD_3_OD): δ 8.08 (d, *J* = 5.4 Hz, 1H, ArH3), 7.98 (d, *J* = 8.4 Hz, 1H, ArH5), 7.79 (d, *J* = 5.4 Hz, 1H, ArH4), 7.02 (s, 1H, ArH8), 6.84 (d, *J* = 9 Hz, 1H, ArH6), 4.11 (t, *J* = 6 Hz, 2H, CH_2_), 3.68 (s, 3H, CH_3_), 2.75 (s, 3H, CH_3_), 2.56 (t, *J* = 7.2 Hz, 2H, CH_2_), 2.13 (t, *J* = 6.6 Hz, 2H, CH_2_); HRMS (ESI): *m*/*z* [M + H]^+^ calcd for C_17_H_19_N_2_O_3_^+^: 299.1390, found: 299.1393; Purity >95%.

*5-(7-Oxy-1-methyl-9-H-b-carbolin)pentanoic acid methyl ester* (**1-2d**), white solid. Yield 50%. ^1^H-NMR (600 MHz, CD_3_OD): δ 8.08 (d, *J* = 5.4 Hz, 1H, ArH3), 7.96 (d, *J* = 8.4 Hz, 1H, ArH5), 7.79 (d, *J* = 6 Hz, 1H, ArH4), 7.02 (d, *J* = 1.8 Hz, 1H, ArH8), 6.84 (m, 1H, ArH6), 4.10 (t, *J* = 6 Hz, 2H, CH_2_), 3.65 (s, 3H, CH_3_), 2.75 (s, 3H, CH_3_), 2.45 (t, *J* = 7.2 Hz, 2H, CH_2_), 1.85 (m, 4H, CH_2_); HRMS (ESI): *m*/*z* [M + H]^+^ calcd for C_18_H_21_N_2_O_3_^+^: 313.1547, found: 313.1554; Purity >95%.

*6-(7-Oxy-1-methyl-9-H-b-carbolin)hexanoic acid methyl ester* (**1-2e**), white solid. Yield 50%. ^1^H-NMR (600 MHz, CD_3_OD): δ 8.08 (d, *J* = 5.4 Hz, 1H, ArH3), 7.97 (d, *J* = 9 Hz, 1H, ArH5), 7.78 (d, *J* = 5.4 Hz, 1H, ArH4), 7.00 (d, *J* = 2.4 Hz, 1H, ArH8), 6.84 (m, 1H, ArH6), 4.06 (t, *J* = 6 Hz, 2H, CH_2_), 3.65 (s, 3H, CH_3_), 2.75 (s, 3H, CH_3_), 2.37 (t, *J* = 7.8 Hz, 2H, CH_2_), 1.84 (m, 2H, CH_2_), 1.70 (m, 2H, CH_2_), 1.54 (m, 2H, CH_2_); HRMS (ESI): *m*/*z* [M + H]+ calcd for C_19_H_23_N_2_O_3_^+^: 327.1709, found: 327.1703; Purity >95%.

*2-(7-Oxy-1-methyl-9-H-b-carbolin)acetic acid tert-butyl ester* (**1-2f**), white solid. Yield 42%. ^1^H-NMR (600 MHz, *d_6_*-DMSO): δ 11.49 (s, 1H, NH), 8.16 (d, *J* = 5.4 Hz, 1H, ArH3), 8.07 (d, *J* = 8.4 Hz, 1H, ArH5), 7.84 (d, *J* = 5.4 Hz, 1H, ArH4), 6.93 (d, *J* = 2.4 Hz, 1H, ArH8), 6.84 (m, 1H, ArH6), 4.77 (s, 2H, CH_2_), 2.73 (s, 3H, CH_3_), 1.45 (s, 9H, (CH_3_)_3_); MS (ESI) *m/z* 313.81 [M + H]^+^.

*3-(7-Oxy-1-methyl-9-H-b-carbolin)propionic acid tert-butyl ester* (**1-2g**), white solid. Yield 32%. ^1^H-NMR (600 MHz, *d_6_*-DMSO): δ 9.82 (s, 1H, NH), 8.13 (d, *J* = 4.8 Hz, 1H, ArH3), 7.98 (d, *J* = 8.4 Hz, 1H, ArH5), 7.80 (d, *J* = 4.8 Hz, 1H, ArH4), 6.93 (d, *J* = 1.2 Hz, 1H, ArH8), 6.74 (m, 1H, ArH6), 4.72 (t, *J* = 7.2 Hz, 2H, CH_2_), 2.92 (s, 3H, CH_3_), 2.67 (t, *J* = 7.2 Hz, 2H, CH_2_), 1.24 (s, 9H, (CH_3_)_3_); MS (ESI) *m/z* 327.22 [M + H]^+^.

*4-(7-Oxy-1-methyl-9-H-b-carbolin)butnoic acid tert-butyl ester* (**1-2h**), White solid. Yield 55%. ^1^H-NMR (600 MHz, *d_6_*-DMSO): δ 11.37 (s, 1H, NH), 8.13 (d, *J* = 5.4 Hz, 1H, ArH3), 8.03 (d, *J* = 9 Hz, 1H, ArH5), 7.80 (d, *J* = 5.4 Hz, 1H, ArH4), 6.98 (d, *J* = 1.8 Hz, 1H, ArH8), 6.83 (m, 1H, ArH6), 4.08 (t, *J* = 6.6 Hz, 2H, CH_2_), 2.71 (s, 3H, CH_3_), 2.41 (t, *J* = 7.2 Hz, 2H, CH_2_), 1.99 (m, 2H, CH_2_), 1.41 (s, 9H, (CH_3_)_3_); MS (ESI) *m/z* 341.17 [M + H]^+^.

*5-(7-Oxy-1-methyl-9-H-b-carbolin)pentanoic acid tert-butyl ester* (**1-2i**), white solid. Yield 40%. ^1^H-NMR (600 MHz, CDCl_3_): δ 9.40 (s, 1H, NH), 8.31 (d, *J* = 4.8 Hz, 1H, ArH3), 7.95 (d, *J* = 8.4 Hz, 1H, ArH5), 7.72 (d, *J* = 5.4 Hz, 1H, ArH4), 6.89 (d, *J* = 1.8 Hz, 1H, ArH8), 6.86 (m, 1H, ArH6), 4.00 (t, *J* = 6 Hz, 2H, CH_2_), 2.79 (s, 3H, CH_3_), 2.31 (t, *J* = 6.6 Hz, 2H, CH_2_), 1.81 (m, 4H, CH_2_), 1.45 (s, 9H, (CH_3_)_3_); MS (ESI) *m/z* 355.63 [M + H]^+^.

*6-(7-Oxy-1-methyl-9-H-b-carbolin)hexanoic acid tert-butyl ester (**1-2j**),* white solid. Yield 39%. ^1^H-NMR (600 MHz, CDCl_3_): δ 9.75 (s, 1H, NH), 8.24 (d, *J* = 5.4 Hz, 1H, ArH3), 7.92 (d, *J* = 8.4 Hz, 1H, ArH5), 7.73 (d, *J* = 5.4 Hz, 1H, ArH4), 7.00 (s, 1H, ArH8), 6.85 (d, *J* = 8.4 Hz, 1H, ArH6), 3.99 (t, *J* = 6.6 Hz, 2H, CH_2_), 2.84 (s, 3H, CH_3_), 2.25 (t, *J* = 7.8 Hz, 2H, CH_2_), 1.81 (m, 2H, CH_2_), 1.66 (m, 2H, CH_2_), 1.49 (m, 2H, CH_2_), 1.44 (s, 9H, (CH_3_)_3_); MS (ESI) *m/z* 369.77 [M + H]^+^.

*2-(7-Oxy-1-methyl-9-H-b-carbolin)ethyl tert-butyl carbamate (**1-2k**),* white solid. Yield 67%. ^1^H-NMR (600 MHz, CD_3_OD): δ 8.08 (d, *J* = 5.4 Hz, 1H, ArH3), 7.98 (d, *J* = 9 Hz, 1H, ArH5), 7.80 (d, *J* = 5.4 Hz, 1H, ArH4), 7.05 (d, *J* = 1.8 Hz, 1H, ArH8), 6.88 (m, 1H, ArH6), 4.10 (t, *J* = 5.4 Hz, 2H, CH_2_), 3.48 (t, *J* = 6 Hz, 2H, CH_2_), 2.75 (s, 3H, CH_3_), 1.44 (s, 9H, (CH_3_)_3_); HRMS (ESI): *m*/*z* [M + H]^+^ calcd for C_19_H_24_N_3_O_3_^+^: 342.1812, found: 342.1822; Purity >95%.

*3-(7-Oxy-1-methyl-9-H-b-carbolin)propyl tert-butyl carbamate (**1-2l**),* white solid. Yield 57%. ^1^H-NMR (600 MHz, *d_6_*-DMSO): δ 8.13 (d, *J* = 4.8 Hz, 1H, ArH3), 8.04 (d, *J* = 8.4 Hz, 1H, ArH5), 7.79 (d, *J* = 4.8 Hz, 1H, ArH4), 6.97 (d, *J* = 1.8 Hz, 1H, ArH8), 6.94 (t, *J* = 5.4 Hz, 1H, NH), 6.82 (m, 1H, ArH6), 4.07 (t, *J* = 6.6 Hz, 2H, CH_2_), 3.12 (t, *J* = 6.6 Hz, 2H, CH_2_), 2.71 (s, 3H, CH_3_), 1.89 (m, 2 H, CH_2_), 1.38 (s, 9H, (CH_3_)_3_); HRMS (ESI): *m*/*z* [M + H]^+^ calcd for C_20_H_26_N_3_O_3_^+^: 356.1969, found: 356.1982; Purity >95%.

*4-(7-Oxy-1-methyl-9-H-b-carbolin)butyl tert-butyl carbamate* (**1-2m**), white solid. Yield 67%. ^1^H-NMR (600 MHz, *d_6_*-DMSO): δ 8.13 (d, *J* = 4.8 Hz, 1H, ArH3), 8.04 (d, *J* = 9 Hz, 1H, ArH5), 7.81 (d, *J* = 5.4 Hz, 1H, ArH4), 6.97 (d, *J* = 2.4 Hz, 1H, ArH8), 6.87 (t, *J* = 5.4 Hz, 1H, NH), 6.82 (m, 1H, ArH6), 4.06 (t, *J* = 6 Hz, 2H, CH_2_), 2.99 (m, 2H, CH_2_), 2.71 (s, 3H, CH_3_), 1.75 (m, 2H, CH_2_), 1.56 (m, 2H, CH_2_), 1.37 (s, 9H, (CH_3_)_3_); HRMS (ESI): *m*/*z* [M + H]^+^ calcd for C_21_H_28_N_3_O_3_^+^: 370.2125, found: 370.2189; Purity >95%.

*2-((7-Oxy-1-methyl-9-H-b-carbolin)ethoxy)ethyl tert-butyl carbamate* (**1-2n**), white solid. Yield 46%. ^1^H-NMR (600 MHz, *d_6_*-DMSO): δ 8.13 (d, *J* = 4.8 Hz, 1H, ArH3), 8.04 (d, *J* = 8.4 Hz, 1H, ArH5), 7.80 (d, *J* = 5.4 Hz, 1H, ArH4), 7.00 (d, *J* = 1.8 Hz, 1H, ArH8), 6.84 (m, 1H, ArH6), 6.81 (t, *J* = 5.4 Hz, 1H, NH), 4.19 (t, *J* = 4.8 Hz, 2H, CH_2_), 3.78 (t, *J* = 4.2 Hz, 2H, CH_2_), 3.48 (t, *J* = 6 Hz, 2H, CH_2_), 3.12 (m, 2H, CH_2_), 2.71 (s, 3H, CH_3_), 1.37 (s, 9H, (CH_3_)_3_); HRMS (ESI): *m*/*z* [M + H]^+^ calcd for C_21_H_28_N_3_O_4_^+^: 386.2074, found: 386.2110; Purity >95%.

*2-(7-oxy-1-methyl-9-H-b-carbolin)methoxyethyl* (**1-2o**), white solid. Yield 38%. ^1^H-NMR (600 MHz, CD_3_OD): δ 8.10 (d, *J* = 6 Hz, 1H, ArH3), 8.01 (d, *J* = 8.4 Hz, 1H, ArH5), 7.83 (d, *J* = 5.4 Hz, 1H, ArH4), 7.05 (d, *J* = 2.4 Hz, 1H, ArH8), 6.89 (m, 1H, ArH6), 4.22 (t, *J* = 4.8 Hz, 2H, CH_2_), 3.80 (t, *J* = 4.2 Hz, 2H, CH_2_), 3.45 (s, 3H, CH_3_), 2.77 (s, 3H, CH_3_); HRMS (ESI): *m*/*z* [M + H]^+^ calcd for C_15_H_17_N_2_O_2_^+^: 257.1285, found: 257.1291; Purity >95%.

*3-(7-Oxy-1-methyl-9-H-b-carbolin)ethoxyethyl (**1-2p**),* white solid. Yield 48%. ^1^H-NMR (600 MHz, CD_3_OD): δ 8.10 (d, *J* = 5.4 Hz, 1H, ArH3), 8.0 (d, *J* = 8.4 Hz, 1H, ArH5), 7.80 (d, *J* = 5.4 Hz, 1H, ArH4), 7.06 (d, *J* = 1.2 Hz, 1H, ArH8), 6.90 (m, 1H, ArH7), 4.22 (t, *J* = 4.8 Hz, 2H, CH_2_), 3.84 (t, *J* = 4.2 Hz, 2H, CH_2_), 3.63 (m, 2H, CH_2_), 3.45 (s, 3H, CH_3_), 2.76 (s, 3H, CH_3_), 1.23 (t, *J* = 6.6 Hz, 2H, CH_2_); HRMS (ESI): *m*/*z* [M + H]^+^ calcd for C_16_H_19_N_2_O_2_^+^: 271.1441, found: 271.1429; Purity >95%.

General procedure for the synthesis of (**1-3**), a solution of **1-2a** to **1-2e** (0.175 mmol) and 7N ammonia in methanol (4 mL) in a sealed pressure vessel was stirred at 90 °C for 12 h. After the completion of the reaction, the mixture was evaporated and purified by flash column chromatography using DCM/MeOH/Ammonia (90/9/1) as eluent to give the desired final compound **1-3** as white solid.

*2-(7-Oxy-1-methyl-9-H-b-carbolin)acetamide* (**1-3a**), white solid. Yield 91%. ^1^H-NMR (600 MHz, *d_6_*-DMSO): δ 8.14 (d, *J* = 5.4 Hz, 1H, ArH3), 8.07 (d, *J* = 8.4 Hz, 1H, ArH5), 7.80 (d, *J* = 5.4 Hz, 1H, ArH4), 7.63 (s, 1H, NH), 7.46 (s, 1H, NH), 7.0 (d, *J* = 1.8 Hz, 1H, ArH8), 6.90 (m, 1H, ArH6), 4.53 (s, 2H, CH_2_), 2.71 (s, 3H, CH_3_); HRMS (ESI): *m*/*z* [M + H]^+^ calcd for C_14_H_14_N_3_O_2_^+^: 256.1081, found: 256.1084; Purity >95%.

*3-(7-Oxy-1-methyl-9-H-b-carbolin)propionamide* (**1-3b**), white solid. Yield 97%. ^1^H-NMR (600 MHz, *d_6_*-DMSO): δ 8.13 (d, *J* = 5.4 Hz, 1H, ArH3), 7.98 (d, *J* = 8.4 Hz, 1H, ArH5), 7.81 (d, *J* = 5.4 Hz, 1H, ArH4), 7.44 (s, 1H, NH), 6.96 (s, 2H, ArH8, NH), 6.73 (m, 1H, ArH6), 4.67 (t, *J* = 7.8 Hz, 2H, CH_2_), 2.95 (s, 3H, CH_3_), 2.54 (t, *J* = 7.8 Hz, 2H, CH_2_); HRMS (ESI): *m*/*z* [M + H]^+^ calcd for C_15_H_16_N_3_O_2_+: 270.1237, found: 270.1245; Purity >95%.

*4-(7-Oxy-1-methyl-9-H-b-carbolin)butanamide* (**1-3c**), white solid. Yield 84%. ^1^H-NMR (600 MHz, *d_6_*-DMSO): δ 8.13 (d, *J* = 5.4 Hz, 1H, ArH3), 8.03 333e3(d, *J* = 8.4 Hz, 1H, ArH5), 7.80 (d, *J* = 5.4 Hz, 1H, ArH4), 7.35 (s, 1H, NH), 6.98 (s, 1H, ArH8), 6.82 (m, 2H, ArH6, NH), 4.06 (t, *J* = 6 Hz, 2H, CH_2_), 2.71 (s, 3H, CH_3_), 2.27 (t, *J* = 7.8 Hz, 2H, CH_2_), 1.98 (m, 2H, CH_2_); HRMS (ESI): *m*/*z* [M + H]^+^ calcd for C_16_H_18_N_3_O_2_^+^: 284.1394, found: 284.1396; Purity >95%.

*5-(7-Oxy-1-methyl-9-H-b-carbolin)pentanamide* (**1-3d**), white solid. Yield 85%. ^1^H-NMR (600 MHz, *d_6_*-DMSO): δ 8.13 (d, *J* = 5.4 Hz, 1H, ArH3), 8.03 (d, *J* = 8.4 Hz, 1H, ArH5), 7.80 (d, *J* = 5.4 Hz, 1H, ArH4), 7.29 (s, 1H, NH), 6.98 (d, *J* = 2.4 Hz,1H, ArH8), 6.82 (m, 1H, ArH6), 6.75 (s, 1H, NH), 4.07 (t, *J* = 6.6 Hz, 2H, CH_2_), 2.71 (s, 3H, CH_3_), 2.13 (t, *J* = 7.2 Hz, 2H, CH_2_), 1.76 (m, 2H, CH_2_), 1.69 (m, 2H, CH_2_); HRMS (ESI): *m*/*z* [M + H]^+^ calcd for C_17_H_20_N_3_O_2_^+^: 298.1550, found: 298.1560; Purity >95%.

*6-(7-Oxy-1-methyl-9-H-b-carbolin)hexanamide* (**1-3e**), white solid. Yield 94%. ^1^H-NMR (600 MHz, *d_6_*-DMSO): δ 8.13 (d, *J* = 5.4 Hz, 1H, ArH3), 8.03 (d, *J* = 8.4 Hz, 1H, ArH5), 7.80 (d, *J* = 4.8 Hz, 1H, ArH4), 7.26 (s, 1H, NH), 6.98 (d, *J* = 2.4 Hz,1H, ArH8), 6.82 (m, 1H, ArH6), 6.27 (s, 1H, NH), 4.06 (t, *J* = 6.6 Hz, 2H, CH_2_), 2.71 (s, 3H, CH_3_), 2.09 (t, *J* = 7.2 Hz, 2H, CH_2_), 1.77 (m, 2H, CH_2_), 1.57 (m, 2H, CH_2_), 1.45 (m, 2H, CH_2_); HRMS (ESI): *m*/*z* [M + H]^+^ calcd for C_18_H_22_N_3_O_2_^+^: 312.1707, found: 312.1728; Purity >95%.

General procedure for the synthesis of (**1-4**), a solution of **1-2f** to **1-2j** (0.18 mmol) and 4N hydrochloric acid in dioxane (4 mL) was stirred at room temperature for 24 h. The reaction mixture was evaporated and triturated with diethyl ether to get the desired acid **1-4** as white solid.

*2-(7-Oxy-1-methyl-9-H-b-carbolin)acetic acid* (**1-4a**), white solid. Yield 89%. ^1^H-NMR (600 MHz, *d_6_*-DMSO): δ 8.48 (d, *J* = 6 Hz, 1H, ArH3), 8.43 (d, *J* = 6 Hz, 1H, ArH4), 8.38 (d, *J* = 9 Hz, 1H, ArH5), 7.09 (m, 2H, ArH6, ArH8), 4.90 (s, 2H, CH_2_), 2.97 (s, 3H, CH_3_); HRMS (ESI): *m*/*z* [M + H]^+^ calcd for C_14_H_13_N_2_O_3_^+^: 257.0921, found: 257.0927; Purity >95%.

*3-(7-Oxy-1-methyl-9-H-b-carbolin)propionic acid* (**1-4b**), white solid. Yield 98%. ^1^H-NMR (600 MHz, *d_6_*-DMSO): δ 8.45 (d, *J* = 6 Hz, 1H, ArH3), 8.40 (d, *J* = 6.6 Hz, 1H, ArH4), 8.30 (d, *J* = 9 Hz, 1H, ArH5), 7.14 (s, 1H, ArH8), 6.97 (d, *J* = 9 Hz, 1H, ArH6), 4.81 (t, *J* = 7.8 Hz, 2H, CH_2_), 3.16 (s, 3H, CH_3_), 2.82 (t, *J* = 7.2 Hz, 2H, CH_2_); HRMS (ESI): *m*/*z* [M + H]^+^ calcd for C_15_H_15_N_2_O_3_^+^: 271.1077, found: 271.1075; Purity >95%.

*4-(7-Oxy-1-methyl-9-H-b-carbolin)butnoic acid* (**1-4c**), white solid. Yield 82%. ^1^H-NMR (600 MHz, *d_6_*-DMSO): δ 8.45 (d, *J* = 6 Hz, 1H, ArH3), 8.40 (d, *J* = 6 Hz, 1H, ArH4), 8.35 (d, *J* = 9 Hz, 1H, ArH5), 7.12 (s, 1H, ArH8), 7.05 (d, *J* = 9 Hz, 1H, ArH6), 4.17 (t, *J* = 6 Hz, 2H, CH_2_), 2.97 (s, 3H, CH_3_), 2.45 (t, *J* = 7.2 Hz, 2H, CH_2_), 2.03 (m, 2H, CH_2_); HRMS (ESI): *m*/*z* [M + H]^+^ calcd for C_16_H_17_N_2_O_3_^+^: 285.1234, found: 285.1236; Purity >95%.

*5-(7-Oxy-1-methyl-9-H-b-carbolin)pentanoic acid* (**1-4d**), white solid. Yield 85%. ^1^H-NMR (600 MHz, *d_6_*-DMSO): δ 8.45 (d, *J* = 6.6 Hz, 1H, ArH3), 8.40 (d, *J* = 6 Hz, 1H, ArH4), 8.35 (d, *J* = 9 Hz, 1H, ArH5), 7.11 (s, 1H, ArH8), 7.05 (d, *J* = 9 Hz, 1H, ArH6), 4.16 (t, *J* = 6.6 Hz, 2H, CH_2_), 2.97 (s, 3H, CH_3_), 2.32 (t, *J* = 7.2 Hz, 2H, CH_2_), 1.83 (m, 2H, CH_2_), 1.72 (m, 2H, CH_2_); HRMS (ESI): *m*/*z* [M + H]^+^ calcd for C_17_H_19_N_2_O_3_^+^: 299.1390, found: 299.1401; Purity >95%.

*6-(7-Oxy-1-methyl-9-H-b-carbolin)hexanoic acid* (**1-4e**), White solid. Yield 91%. ^1^H-NMR (600 MHz, *d_6_*-DMSO): δ 8.46 (d, *J* = 6.6 Hz, 1H, ArH3), 8.40 (d, *J* = 6 Hz, 1H, ArH4), 8.35 (d, *J* = 9 Hz, 1H, ArH5), 7.11 (s, 1H, ArH8), 7.05 (d, *J* = 9 Hz, 1H, ArH6), 4.14 (t, *J* = 6.6 Hz, 2H, CH_2_), 2.97 (s, 3H, CH_3_), 2.26 (t, *J* = 7.8 Hz, 2H, CH_2_), 1.80 (m, 2H, CH_2_), 1.59 (m, 2H, CH_2_), 1.49 (m, 2H, CH_2_); HRMS (ESI): *m*/*z* [M + H]^+^ calcd for C_18_H_21_N_2_O_3_^+^: 313.1547, found: 313.1557; Purity >95%.

General procedure for the synthesis of (**1-5**), to a solution of **1-2k** to **1-2n** (1.93 mmol) in 1,4-dioxane (4 mL) was added 4N HCl in dioxane (4 eq.) and stirred at room temperature for 24 h. After the completion of the reaction as monitored by LCMS, the mixture was evaporated to give the desired product **10-5** as white solid. 

*2-(7-Oxy-1-methyl-9-H-b-carbolin)ethylamine hydrochloric acid* (**1-5a**), white solid. Yield 90%. ^1^H-NMR (600 MHz, *d_6_*-DMSO): δ 8.47 (d, *J* = 6.6 Hz, 1H, ArH3), 8.38 (m, 2H, ArH4, ArH5), 8.33 (bs, 2H, NH_2_), 7.19 (d, *J* = 1.8 Hz, 1H, ArH8), 7.10 (m, 1H, ArH6), 4.37 (t, *J* = 5.4 Hz, 2H, CH_2_), 3.30 (t, *J* = 4.8 Hz, 2H, CH_2_), 3.03 (s, 3H, CH_3_); HRMS (ESI): *m*/*z* [M + H]^+^ calcd for C_14_H_16_N_3_O^+^: 242.1288, found: 242.1301; Purity >95%.

*3-(7-Oxy-1-methyl-9-H-b-carbolin)propylamine hydrochloric acid (**1-5b**),* white solid. Yield 98%. ^1^H-NMR (600 MHz, *d_6_*-DMSO): δ 8.45 (d, *J* = 6 Hz, 1H, ArH3), 8.38 (m, 2H, ArH4, ArH5), 8.09 (bs, 2H,NH_2_), 7.15 (d, *J* = 2.4 Hz, 1H, ArH8), 7.06 (m, 1H, ArH6), 4.25 (t, *J* = 6 Hz, 2H, CH_2_), 3.03 (m, 5H, CH_2_, CH_3_), 2.12 (t, *J* = 6.6 Hz, 2H, CH_2_); HRMS (ESI): *m*/*z* [M + H]^+^ calcd for C_15_H_18_N_3_O^+^: 256.1444, found: 256.1433; Purity >95%.

*4-(7-Oxy-1-methyl-9-H-b-carbolin)butylamine hydrochloric acid* (**1-5c**), white solid. Yield 99%. ^1^H-NMR (600 MHz, *d_6_*-DMSO): δ 8.45 (d, *J* = 6 Hz, 1H, ArH3), 8.38 (d, *J* = 6 Hz, 1H, ArH4), 8.35 (d, *J* = 8.4 Hz, 1H, ArH5), 7.97 (bs, 2H, NH_2_), 7.14 (d, *J* = 1.8 Hz, 1H, ArH8), 7.05 (d, *J* = 9 Hz, 1H, ArH6), 4.17 (t, *J* = 6 Hz, 2H, CH_2_), 3.02 (s, 3H, CH_3_), 2.87 (t, *J* = 6.6 Hz, 2H, CH_2_), 1.87 (m, 2H, CH_2_), 1.77 (m, 2H, CH_2_); HRMS (ESI): *m*/*z* [M + H]^+^ calcd for C_16_H_20_N_3_O^+^: 270.1601, found: 270.1594; Purity >95%.

*2-((7-Oxy-1-methyl-9-H-b-carbolin)ethoxy)ethylamine hydrochloric acid* (**1-5d**), white solid. Yield 97%. ^1^H-NMR (600 MHz, *d_6_*-DMSO): δ 8.45 (d, *J* = 6 Hz, 1H, ArH3), 8.36 (m, 2H, ArH4, ArH5), 8.10 (bs, 2H, NH_2_), 7.18 (d, *J* = 1.8 Hz, 1H, ArH8), 7.06 (d, *J* = 9 Hz,1H, ArH6), 4.31 (t, *J* = 4.8 Hz, 2H, CH_2_), 3.88 (t, *J* = 4.2 Hz, 2H, CH_2_), 3.73 (t, *J* = 5.4 Hz, 2H, CH_2_), 3.03 (s, 3H, CH_3_), 3.0 (t, *J* = 5.4 Hz, 2H, CH_2_); HRMS (ESI): *m*/*z* [M + H]^+^ calcd for C_16_H_20_N_3_O^+^: 286.1550, found: 286.1552; Purity >95%.

General procedure for the synthesis of (**1-6**), to a solution of **1-5** (0.36 mmol) and triethylamine (2.2 eq.) in DCM was added acetic anhydride dropwise and the reaction mixture was stirred at room temperature for 1 h. Upon completion of the reaction, the mixture was diluted with DCM, transferred to separatory funnel and washed with water. The organic layer was collected, dried over magnesium sulfate, filtered and evaporated to get the desired product **1-6** as white solid. 

*2-(7-Oxy-1-methyl-9-H-b-carbolin)-2-acetyl-ethylamine* (**1-6a**), white solid. Yield 74%. ^1^H-NMR (600 MHz, *d_6_*-DMSO): δ 8.17 (s, 1H, NH), 8.15 (d, *J* = 5.4 Hz, 1H, ArH3), 8.06 (d, *J* = 8.4 Hz, 1H, ArH5), 7.82 (d, *J* = 5.4 Hz, 1H, ArH4), 7.00 (s, 1H, ArH8), 6.84 (m, 1H, ArH6), 4.09 (t, *J* = 6 Hz, 2H, CH_2_), 3.48 (t, *J* = 5.4 Hz, 2H, CH_2_), 2.72 (s, 3H, CH_3_), 1.84 (s, 3H, CH_3_); HRMS (ESI): *m*/*z* [M + H]^+^ calcd for C_16_H_18_N_3_O_2_^+^: 284.1394, found: 284.1419; Purity >95%.

*3-(7-Oxy-1-methyl-9-H-b-carbolin) -2-acetyl-propylamine* (**1-6b**), white solid. Yield 79%. ^1^H-NMR (600 MHz, *d_6_*-DMSO): δ 8.14 (d, *J* = 5.4 Hz, 1H, ArH3), 8.06 (d, *J* = 8.4 Hz, 1H, ArH5), 7.95 (s, 1H, NH), 7.80 (t, *J* = 5.4 Hz, 1H, ArH4), 6.99 (d, *J* = 2.4 Hz, 1H, ArH8), 6.82 (m, 1H, ArH6), 4.08 (t, *J* = 6 Hz, 2H, CH_2_), 3.23 (m, 2H, CH_2_), 2.71 (s, 3H, CH_3_), 1.90 (m, 2H, CH_2_), 1.81 (s, 3H, CH_3_); HRMS (ESI): *m*/*z* [M + H]^+^ calcd for C_17_H_20_N_3_O_2_^+^: 298.1550, found: 298.1552; Purity >95%.

*4-(7-Oxy-1-methyl-9-H-b-carbolin) -2-acetyl-butylamine* (**1-6c**), white solid. Yield 70%. ^1^H-NMR (600 MHz, *d_6_*-DMSO): δ 8.16 (d, *J* = 5.4 Hz, 1H, ArH3), 8.05 (d, *J* = 8.4 Hz, 1H, ArH5), 7.87 (bs, 1H, NH), 7.83 (d, *J* = 5.4 Hz, 1H, ArH4), 6.99 (d, *J* = 1.8 Hz, 1H, ArH8), 6.84 (m, 1H, ArH6), 4.08 (t, *J* = 5.4 Hz, 2H, CH_2_), 3.10 (m, 2H, CH_2_), 2.73 (s, 3H, CH_3_), 1.80 (m, 5H, CH_2_, CH_3_), 1.59 (m, 2H, CH_2_); HRMS (ESI): *m*/*z* [M + H]^+^ calcd for C_18_H_22_N_3_O_2_^+^: 312.1707, found: 312.1724; Purity >95%.

*2-((7-Oxy-1-methyl-9-H-b-carbolin)ethoxy)-2-acetyl-ethylamine* (**1-6d**), white solid. Yield 49%. ^1^H-NMR (600 MHz, *d_6_*-DMSO): δ 8.14 (d, *J* = 5.4 Hz, 1H, ArH3), 8.06 (d, *J* = 9.0 Hz, 1H, ArH5), 7.93 (m, 1H, NH), 7.80 (d, *J* = 5.4 Hz, 1H, ArH4), 7.01 (s, 1H, ArH8), 6.85 (m, 1H, ArH6), 4.20 (t, *J* = 4.8 Hz, 2H, CH_2_), 3.79 (m, 2H, CH_2_), 3.50 (t, *J* = 6 Hz, 2H, CH_2_), 3.55 (m, 2H, CH_2_), 2.71 (s, 3H CH_3_), 1.79 (s, 3H, CH_3_); HRMS (ESI): *m*/*z* [M + H]^+^ calcd for C_18_H_22_N_3_O_3_^+^: 328.1656, found: 328.1669; Purity >95%.

*O-Trifluoromethanesulfonyl-1-methyl-9H-b-carbolin-7-ol* (**1-7**), to a solution of **1-1** (500 mg, 2.52 mmol) and pyridine (25.2 mmol) in DCM (4 mL) was added trifluoromethanesulfonic anhydride (1.2 eq.) at 0°C dropwise. The reaction mixture was allowed to warm to room temperature and stirred for 12 h. Upon completion of the reaction, the mixture was evaporated and purified by flash column chromatography using DCM/MeOH (95/5) as eluent to get the desired product **1-7** as white solid (875 mg, 92%). ^1^H-NMR (600 MHz, *d_6_*-DMSO): δ 13.01 (s, 1H, NH), 8.67 (d, *J* = 9 Hz, 1H, ArH3), 8.65 (d, *J* = 6 Hz, 1H, ArH4), 8.54 (d, *J* = 6 Hz, 1H, ArH5), 7.89 (m, 1H, ArH8), 7.54 (d, *J* = 9 Hz, 1H, ArH6), 3.01 (s, 3H, CH_3_); MS (ESI) *m/z* 331.5 [M + H]^+^.

*7-Nitro-1-methyl-9H-b-carboline* (**1-8**), a pressure vessel was charged with **1-7** (100 mg, 0.30 mmol), Pd_2_(dba)_3_ (5 mol%), BrettPhos (6 mol%) and sodium nitrite (42 mg, 0.6 mmol) and evacuated and back filled with argon three times. To the mixture was added tert-butanol (0.6 mL) and TDA (5 mol%) under argon. The pressure was sealed and heated to 150 °C for 24 h. Upon completion of the reaction, the mixture was evaporated and purified by column chromatography to get **1-8** as yellow solid (80 mg, 77%). ^1^H-NMR (600 MHz, CD_3_OD): δ 8.41 (m, 1H, ArH3), 8.30 (d, *J* = 8.4 Hz, 1H, ArH5), 8.23 (d, *J* = 5.4 Hz, 1H, ArH4), 8.08 (m, 1H, ArH8), 8.01 (d, *J* = 5.4 Hz, 1H, ArH6), 2.82 (s, 3H, CH_3_); MS (ESI) *m/z* 228.31 [M + H]^+^.

*7-Amino-1-methyl-9H-b-carboline* (**1-9**), to a solution of **1-8** (80 mg, 0.35 mmol) and palladium on carbon (10% by wt, 100 mg) in methanol (4 mL) was added hydrazine monohydrate (0.34 mL, 7 mmol) and heated to 85 °C for 1 h. Upon completion of the reaction, catalyst was filtered over Celite and filtrate was evaporated and purified by column chromatography using DCM/MeOH (90/10) as eluent to provide the desired amino compound **1-9** as brown solid (65 mg, 98%). ^1^H-NMR (600 MHz, CD_3_OD): δ 8.02 (d, *J* = 5.4 Hz, 1H, ArH3), 7.82 (d, *J* = 8.4 Hz, 1H, ArH5), 7.69 (d, *J* = 4.8 Hz, 1H, ArH4), 6.80 (s, 1H, ArH8), 6.68 (d, *J* = 8.4 Hz, 1H, ArH9), 2.71 (s, 3H, CH_3_); HRMS (ESI): m/z [M + H]^+^ calcd for C_12_H_12_N_3_^+^: 198.1026, found: 198.1050; Purity >95%.

*7-Acetamide-1-methyl-9H-b-carboline* (**1-10**), to a solution of **1-9** (20 mg, 0.10 mmol) and triethylamine (0.028 mL, 0.20 mmol) in THF (2 mL) was added acetic anhydride dropwise at 0 °C and the reaction mixture was stirred at room temperature for 12 h. Upon completion of the reaction, the mixture was diluted with DCM, transferred to separatory funnel and washed with water. The organic layer was collected, dried over magnesium sulfate, filtered and evaporated to get the desired product **1-10** as white solid (18 mg, 75%). ^1^H-NMR (600 MHz, *d_6_*-DMSO): δ 11.46 (s, 1H, NH), 10.16 (s, 1H, NH), 8.19 (s, 1H, ArH3), 8.15 (d, *J* = 5.4 Hz, 1H, ArH4), 8.06 (d, *J* = 8.4 Hz, 1H, ArH5), 7.81 (s, 1H, ArH8), 7.23 (d, *J* = 8.4 Hz, 1H, ArH6), 2.71 (s, 3H, CH_3_), 2.10 (s, 3H, CH_3_); HRMS (ESI): *m*/*z* [M + H]^+^ calcd for C_14_H_14_N_3_^+^: 240.1131, found: 240.1135; Purity >95%.

*7-Benzamide-1-methyl-9H-b-carboline* (**1-11**), to a solution of **1-9** (20 mg, 0.10 mmol) and triethylamine (0.028 mL, 0.20 mmol) in THF (1 mL) was added benzoyl chloride at 0 °C and stirred at room temperature for 2 h. Upon completion of the reaction monitored by LCMS, the mixture was evaporated and purified by column chromatography using DCM/MeOH (95/5) as eluent to get the desired product **1-11** as white solid (23 mg, 77%). ^1^H-NMR (600 MHz, *d_6_*-DMSO): δ 8.32 (s, 1H, NH), 8.17 (d, *J* = 4.8 Hz, 1H, ArH3), 8.13 (d, *J* = 8.4 Hz, 1H, ArH5), 8.00 (d, *J* = 7.2 Hz, 2H, ArH (benzamido)), 7.85 (d, *J* = 5.4 Hz, 1H, ArH4), 7.61 (m, 1H, ArH8), 7.57 (m, 1H, ArH6); 7.54 (m, 3H, ArH (benzamido)), 2.74 (s, 3H, CH_3_); HRMS (ESI): *m*/*z* [M + H]^+^ calcd for C_19_H_16_N_3_^+^: 302.1288, found: 302.1296; Purity >95%.

*1-Methyl-9H-pyrido[3,4-b]indol-7-yl isopropylcarbamate* (**1-12**), to a solution of harmalol **1-1** (50 mg, 0.25 mmol) in DMF (2 mL) was added isopropyl isocyanate (0.030 mL, 0.3 mmol) and stirred at room temperature for 12 h. The reaction mixture was evaporated and purified by column chromatography using DCM/MeOH (95/5) as eluent to get the desired product **1-12** as white solid (23 mg, 32%). ^1^H-NMR (600 MHz, CD_3_OD): δ 8.15 (d, *J* = 5.4 Hz, 1H, ArH3), 8.12 (d, *J* = 8.4 Hz, 1H, ArH5), 7.90 (d, *J* = 5.4 Hz, 1H, ArH4), 7.31 (m, 1H, ArH8), 7.09 (m, 1H, ArH6), 3.80 (m, 1H, CH), 2.79 (s, 3H, CH_3_), 1.23 (d, *J* = 6.6 Hz, 6H); HRMS (ESI): *m*/*z* [M + H]^+^ calcd for C_16_H_18_N_3_O_2_^+^: 284.1394, found: 284.1416; Purity >95%.

### 3.2. DYRK1A Binding Assays

Compounds were tested for DYRK1A binding activity by a commercial kinase profiling services, Life Technologies which uses the FRET-based LanthaScreen^®^ Eu Kinase Binding Assay. [38] Compounds were screened for DYRK1A activity at concentrations of 1000 nM and 300 nM in duplicates. The IC_50_ was determined by 10 point LanthaScreen^®^ Eu Kinase Binding Assay [38] in duplicates.

### 3.3. β-Cell Proliferation Assay

Human pancreatic islets were obtained from the NIH/NIDDK-supported Integrated Islet Distribution Program (IIDP) Duarte, CA, USA (https://iidp.coh.org) and studied using protocols that have been published in detail [5]. Briefly, islets were first dispersed with Accutase (Sigma, St. Louis, MO, USA) onto coverslips, and treated with vehicle (0.1% DMSO) or with harmine-related compounds in RPMI1640 complete medium for 96 h. Then the cells were then, fixed with formaldehyde and immunolabeled for insulin and Ki67 as described below. Total insulin-positive cells and cells double positive Ki67 and insulin positive cells were imaged and counted. Islets from 3–5 different adult donors were tested as described in Figure 4. At least 1000 β-cells were counted for human islet donor.

### 3.4. Docking

All docking calculations and molecule preparations were carried out using tools in the Schrödinger Small Molecule Drug Discovery suite release 2018-4. Proteins were prepared using the protein preparation wizard and ligands were prepared using ligprep. The target structure for all docking calculations was the crystal structure of DYRK1A in complex with compound **2-2c**. Compound were docked using Glide with XP precision, restricting the Harmine core to its reference position in the **2-2c** complex [50,51,52].

### 3.5. Electrostatic Potential

Electrostatic potential calculation was carried out on the DYRK1A crystal structure in complex with compound **2-2c** using the poisson–boltzmann method as implemented in APBS within Maestro (Schrödinger, LLC). The potential was mapped on the protein surface [53,54].

## 4. Conclusions

In these translational studies, we have reported our efforts to study the harmine 7-position with the objective to identify novel harmine-based DYRK1A inhibitors with human β-cell proliferation activity and chemical potential for targeted delivery. We developed chemistry to carry out modifications of the 7-position of harmine by introducing substituents bearing terminal methyl ester, carboxamide, carboxylic acid and amino/substituted amino groups with chain lengths varying from 1–5 carbons. Two compounds were active at less than 100 nM in the biochemical DYRK1A inhibition assay (**1-2b** (89 nM) and **1-3b** (91nM), respectively), ~3–4-fold less than that of harmine (27 nM). However, the majority of 7-substituted analogs regardless of carbon chain lengths did not show any improvement in DYRK1A inhibitory activity. It can be inferred from modeling studies that harmine 7-position analogs result in disruption of a key binding contact of harmine (H-bond to Leu241) or introduce unproductive electrostatic interactions with the harmine binding pocket. These results indicate that these 7-position modifications are not well tolerated for further optimization of harmine scaffold for β-cell proliferation and conjugation for cell-specific targeted delivery. We did however observe that both of the potent 7-*O* DYRK1A inhibitor harmine analogues, **1-2b** and **1-3b** did exhibit human β-cell proliferation at screening concentration of 10 μM *in vitro*, with analog **1-2b** performing slightly better than **1-3b**. Dose-response experiments further indicated that **1-2b** induced proliferation in a dose-dependent manner, but required 3-fold higher concentration (30 μM) to induce maximal proliferation, 66% that of harmine (10 μM). The reduced β-cell proliferation could be a result of the following: a) its reduced potency for inhibition of DYRK1A as compared to harmine; b) reduced cell permeability; c) reduced DYRK1A cell target engagement; d) off-target kinase activity; e) a combination of these. Collectively, these data indicate that modifications of 7-position of harmine are less promising for retaining potent DYRK1A inhibition for this scaffold as compared to those at the harmine 1- and 9-positions. We did observe that two 7-*O* harmine analogs **1-2b** and **1-3b** showed DYRK1A inhibitory and β-cell proliferation assay activity in vitro only three-fold less that of harmine. It is important to note that this study provides deeper insights into the effects of 7-position modifications of harmine on DYRK1A inhibition in context of diabetes than previously reported, with two novel analogs compounds showing robust β-cell proliferation activity. In conclusion, this study, based on the structure–activity relationships that have been developed, demonstrates that the harmine 7-position is less than optimal to develop a β-cell proliferative and cell-specific targeted diabetes therapeutic from this heterocyclic scaffold.

## Supplementary Materials

NMR spectral data are available in the Supplementary Material.
