# Supplementary material for: Structure–Activity Relationships and Biological Evaluation of 7-Substituted Harmine Analogs for Human β-Cell Proliferation
Source: Molecules. 2020 Apr 23;25(8):1983. doi: 10.3390/molecules25081983 (PMC7221803; doi:10.3390/molecules25081983)
Supplement: Supplementary file 1 [file molecules-25-01983-s001.pdf]

^{1}H-NMR of Compound 1-1

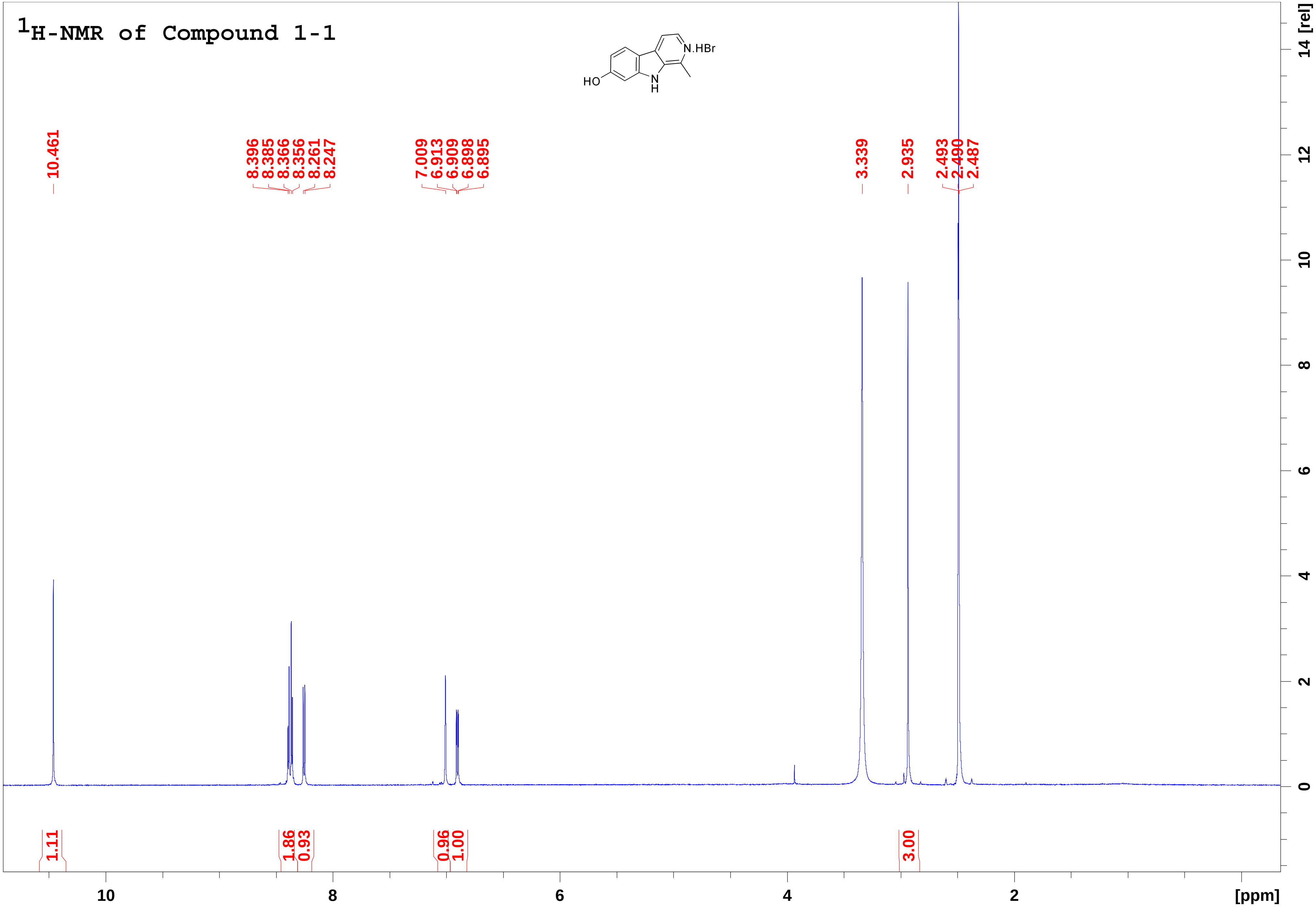

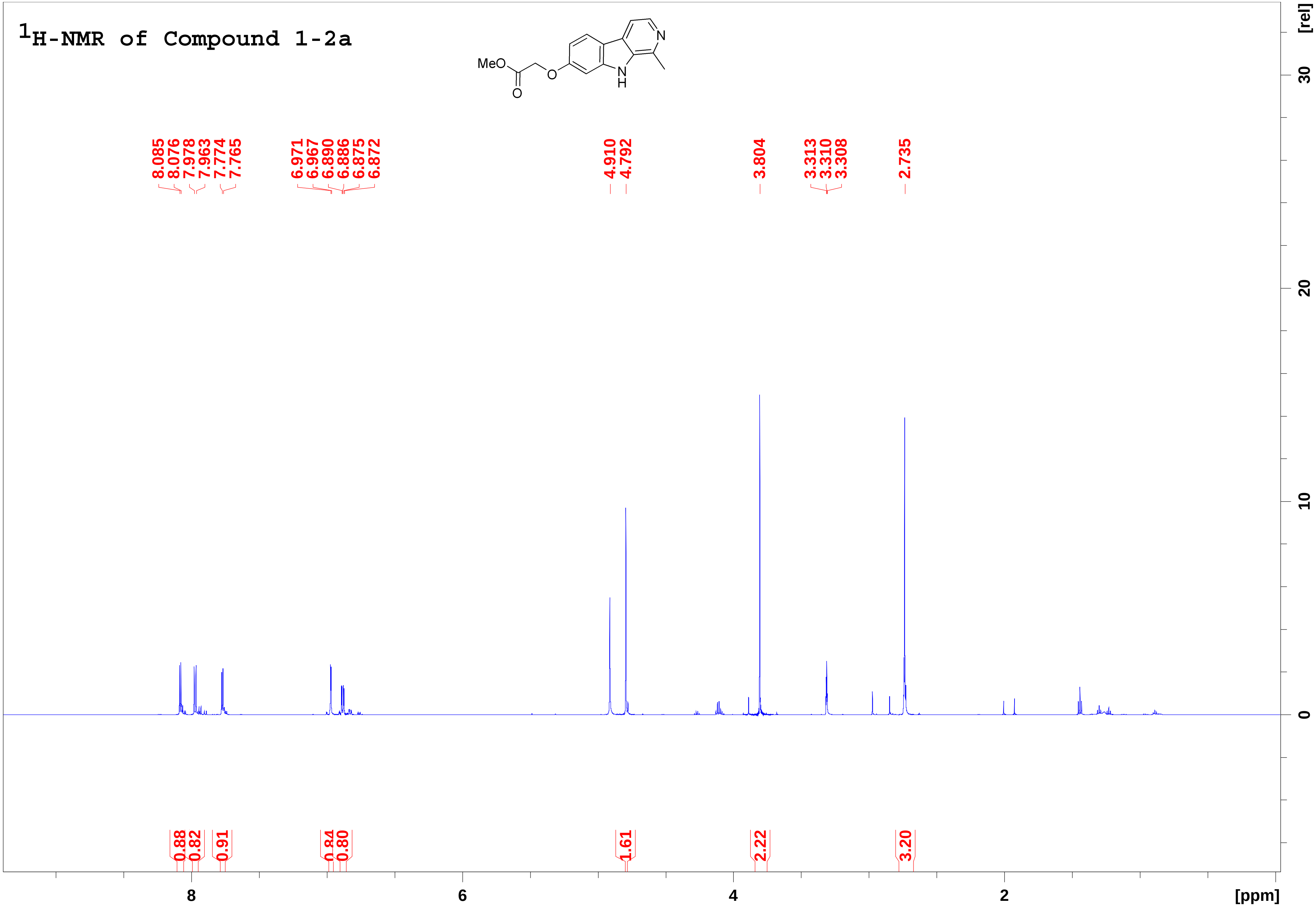

1H-NMR of Compound 1-2a
MeO
O
O
N
H
N
8.085
8.076
7.978
7.963
7.774
7.765
6.971
6.967
6.890
6.886
6.875
6.872
4.910
4.792
3.804
3.313
3.310
3.308
2.735
0.88
0.82
0.91
0.84
0.80
1.61
2.22
3.20
[rel]
30
20
10
0
8
6
4
2
[ppm]

^{1}H-NMR of Compound 1-2b

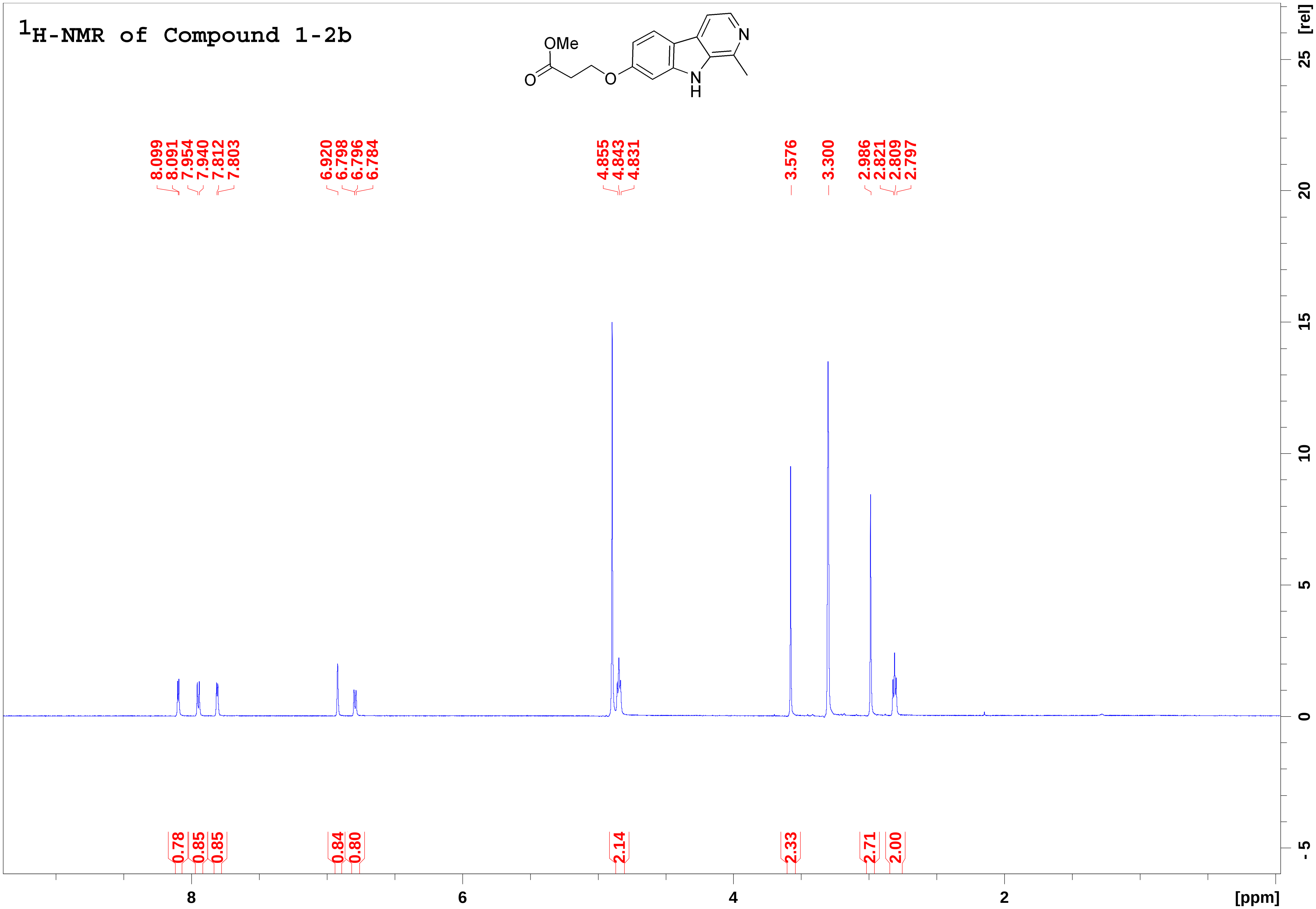

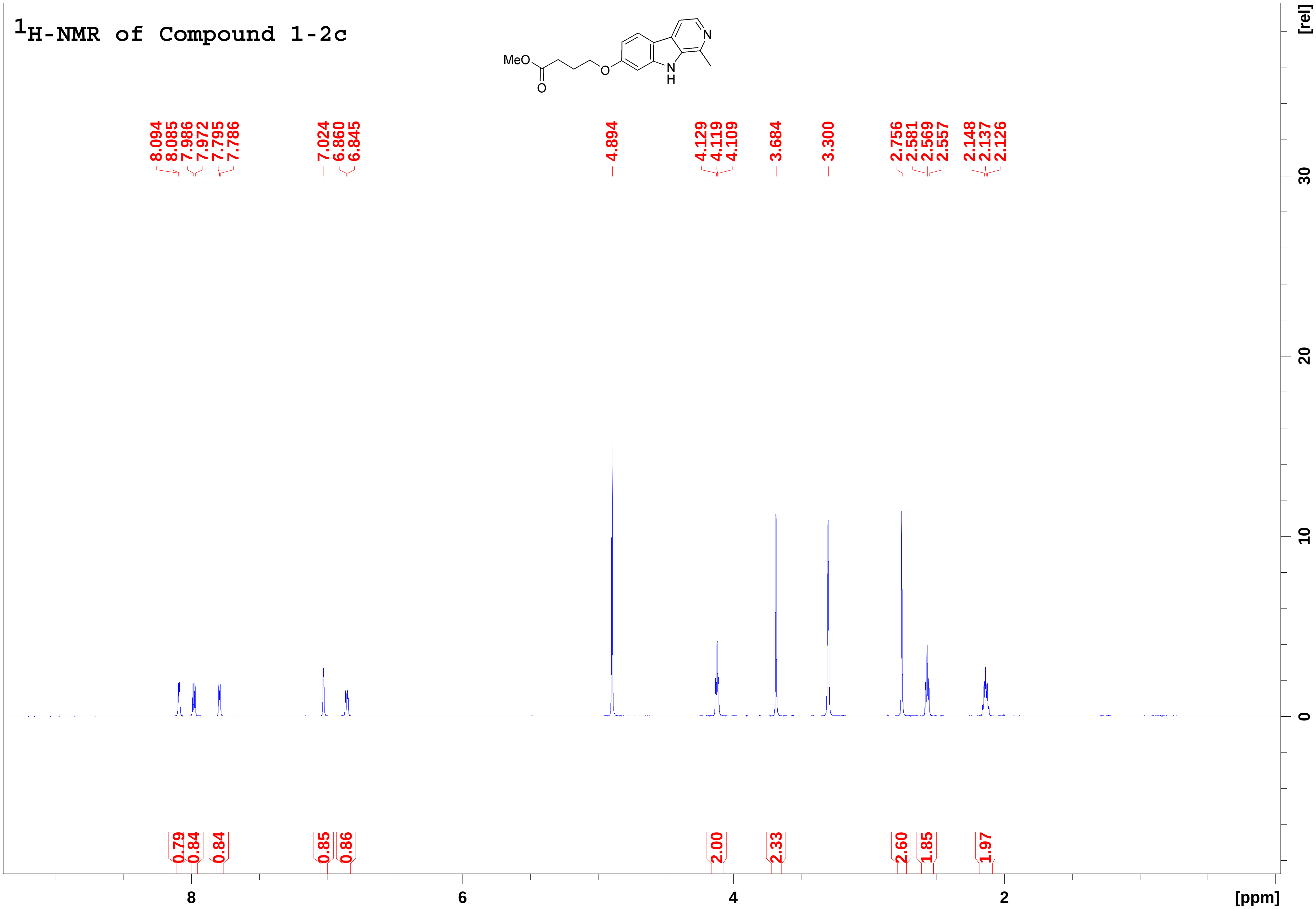
1H-NMR of Compound 1-2c
MeO
O
O
N
N
H
8.094
8.085
7.986
7.972
7.795
7.786
7.024
6.860
6.845
4.894
4.129
4.119
4.109
3.684
3.300
2.756
2.581
2.569
2.557
2.148
2.137
2.126
0.79
0.84
0.84
0.85
0.86
2.00
2.33
2.60
1.85
1.97
[rel]
30
20
10
0
8
6
4
2
[ppm]

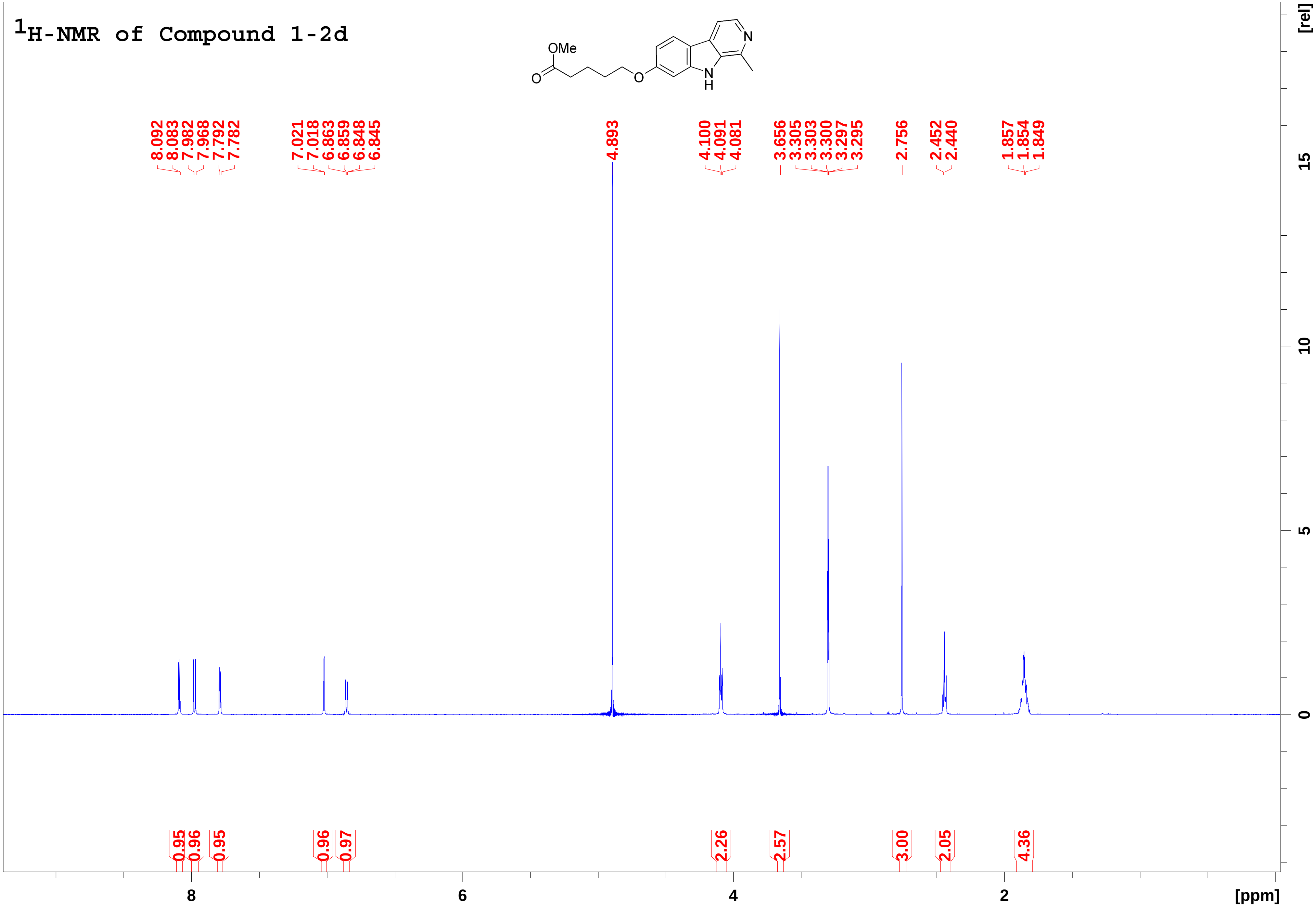
1H-NMR of Compound 1-2d
OMe
O
O
N
H
N
8.092
8.083
7.982
7.968
7.792
7.782
7.021
7.018
6.863
6.859
6.848
6.845
4.893
4.100
4.091
4.081
3.656
3.305
3.303
3.300
3.297
3.295
2.756
2.452
2.440
1.857
1.854
1.849
0.95
0.96
0.95
0.96
0.97
2.26
2.57
3.00
2.05
4.36
8
6
4
2
[ppm]
[rel]
15
10
5
0

^{1}H-NMR of Compound 1-2e

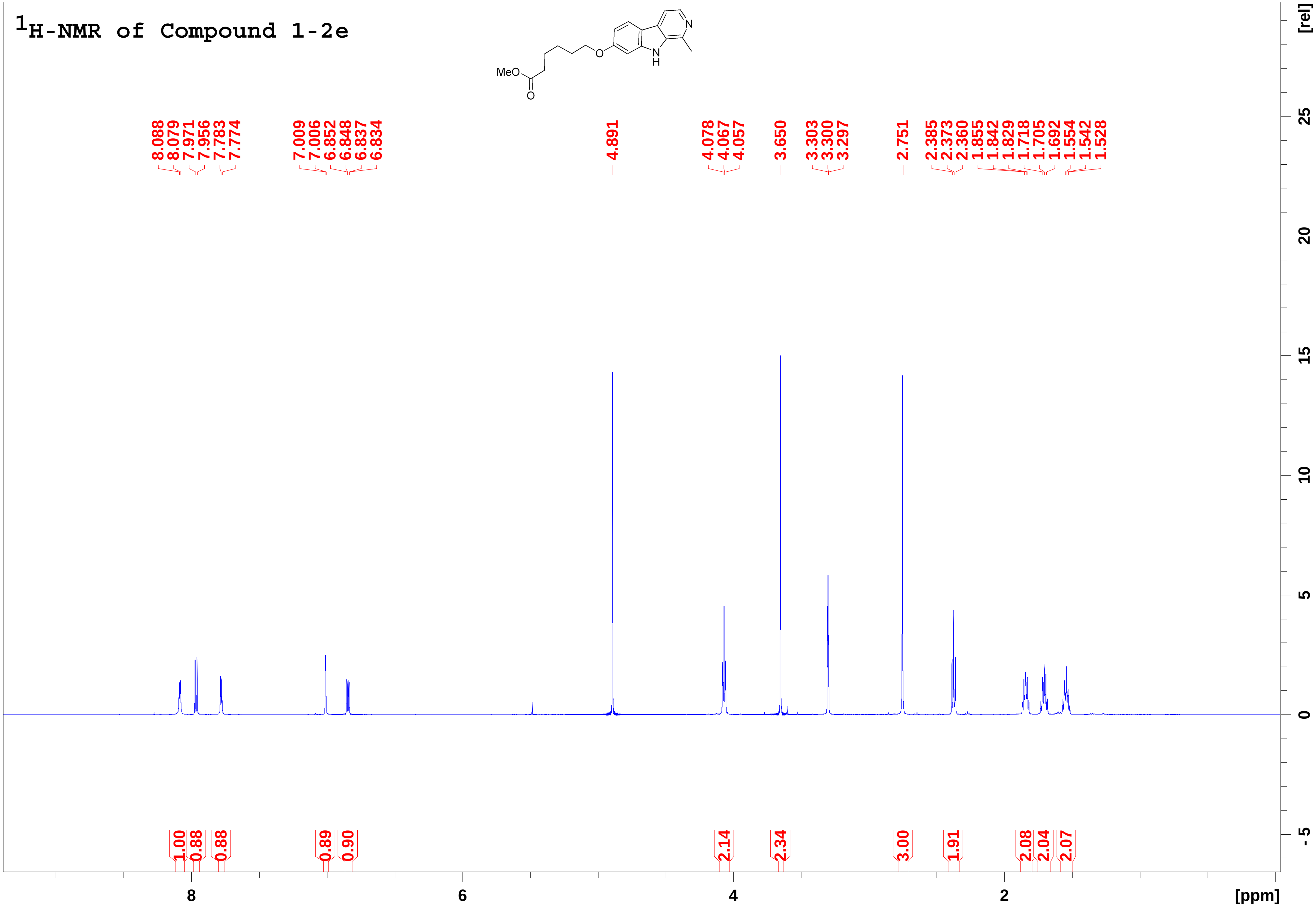

^{1}H-NMR of Compound 1-2f

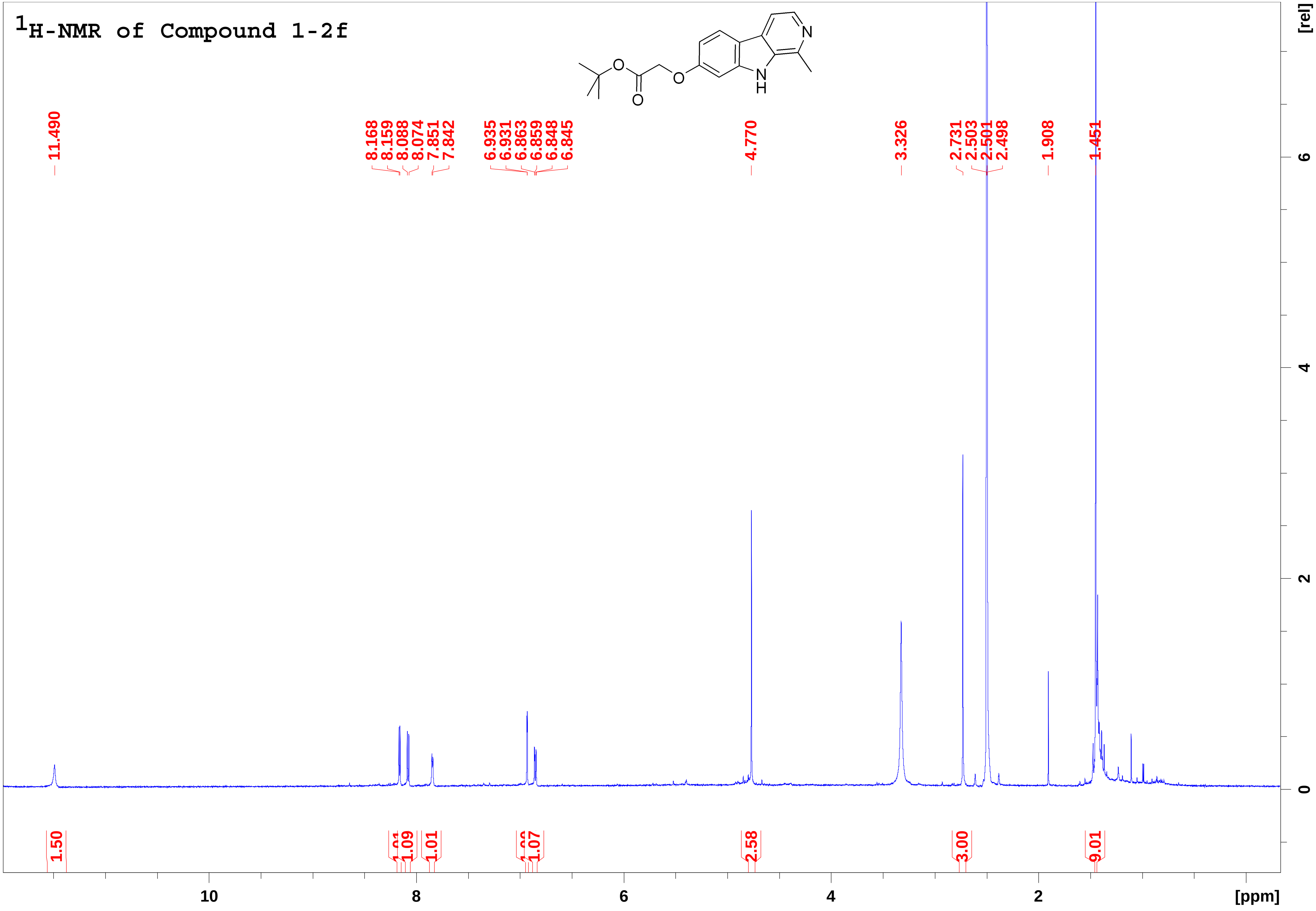

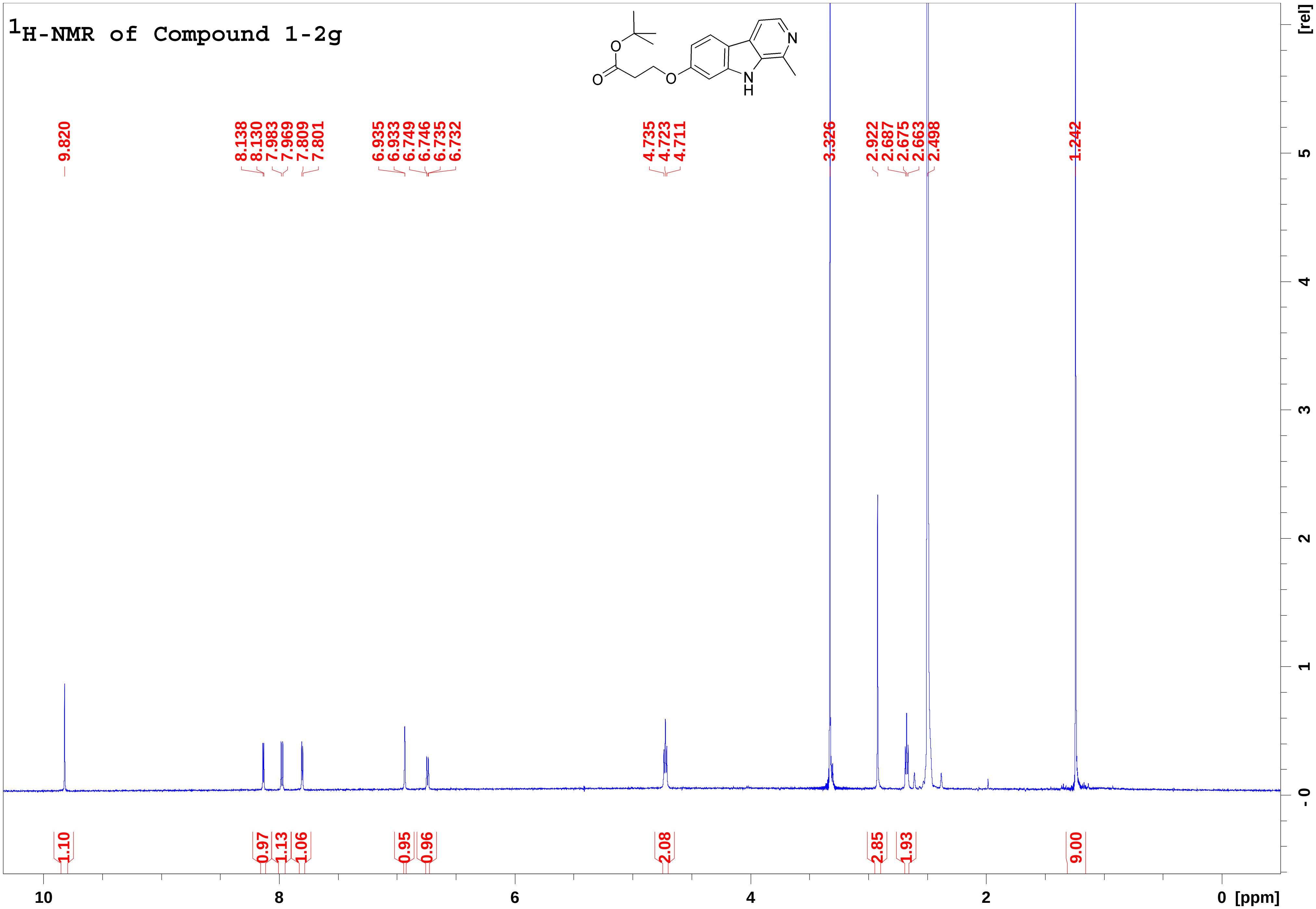
1H-NMR of Compound 1-2g
O
O
O
N
N
H
9.820
8.138
8.130
7.983
7.969
7.809
7.801
6.935
6.933
6.749
6.746
6.735
6.732
4.735
4.723
4.711
3.326
2.922
2.687
2.675
2.663
2.498
1.242
1.10
0.97
1.13
1.06
0.95
0.96
2.08
2.85
1.93
9.00
10
8
6
4
2
0 [ppm]
[rel]
5
4
3
2
1
-0

¹H-NMR of Compound 1-2h

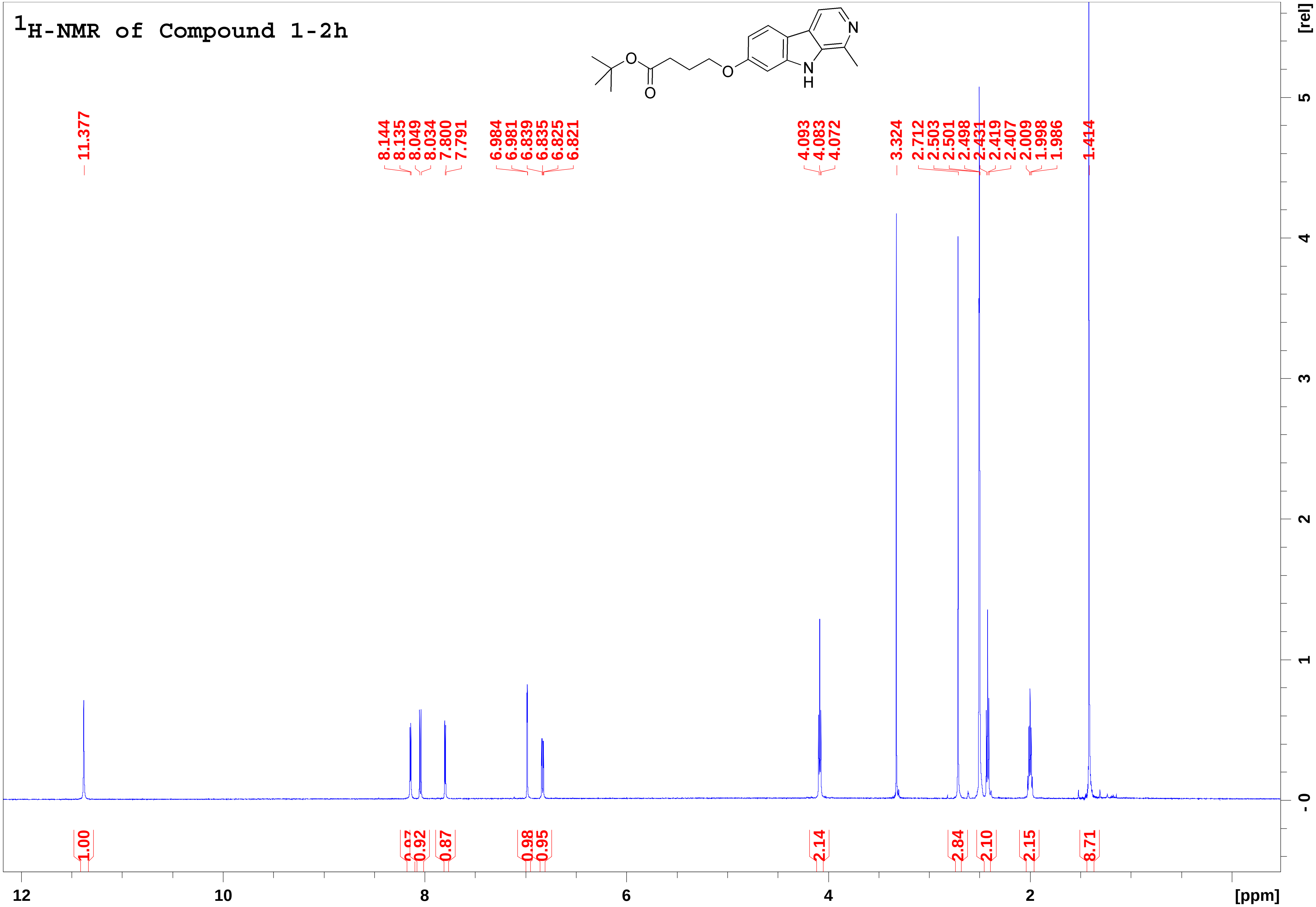

^{1}H-NMR of Compound 1-2i

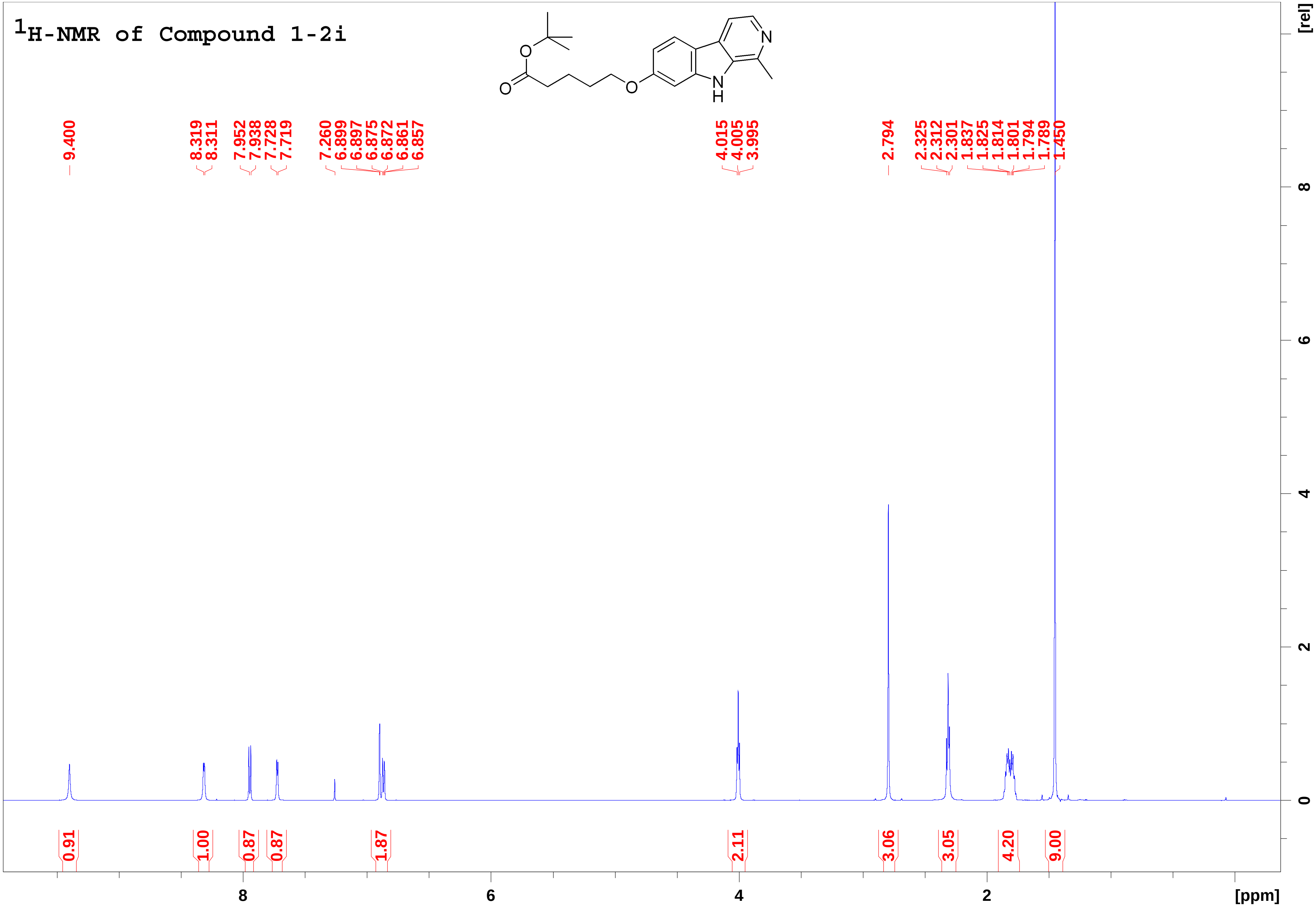

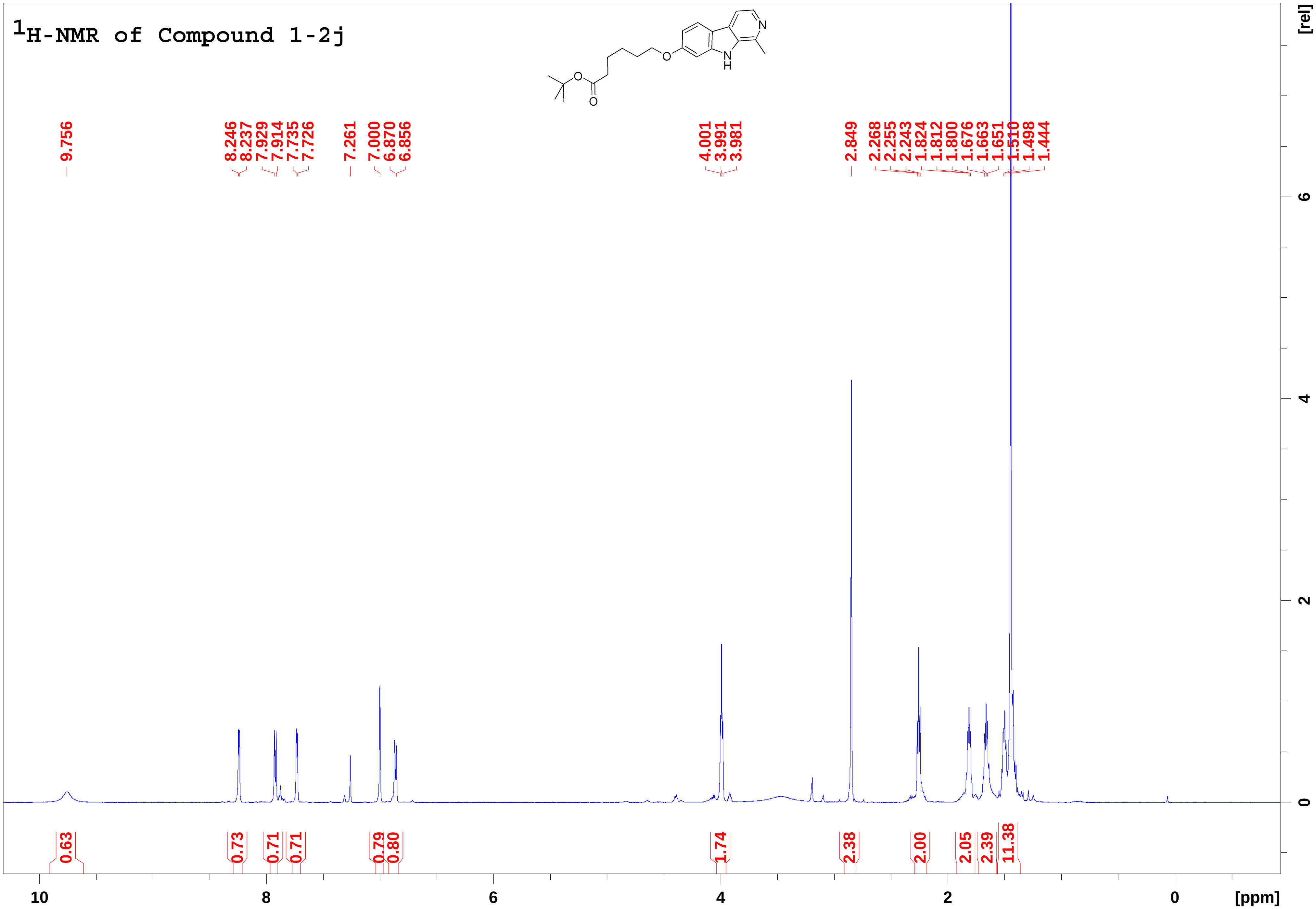

1H-NMR of Compound 1-2j
N
H
O
O
O
N
9.756
8.246
8.237
7.929
7.914
7.735
7.726
7.261
7.000
6.870
6.856
4.001
3.991
3.981
2.849
2.268
2.255
2.243
1.824
1.812
1.800
1.676
1.663
1.651
1.510
1.498
1.444
0.63
0.73
0.71
0.71
0.79
0.80
1.74
2.38
2.00
2.05
2.39
11.38
[rel]
[ppm]

^{1}H-NMR of Compound 1-2k

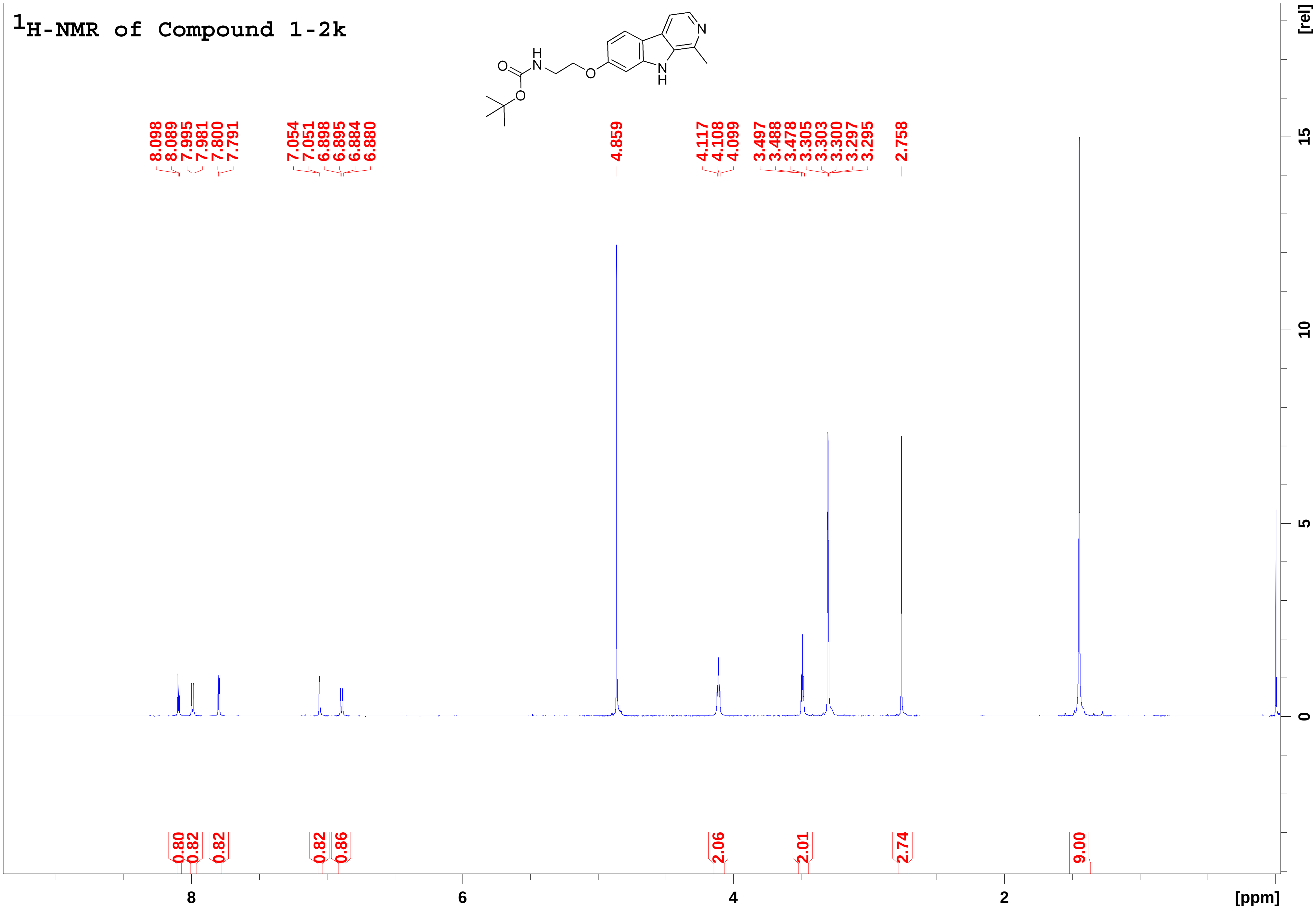

^{1}H-NMR of Compound 1-21

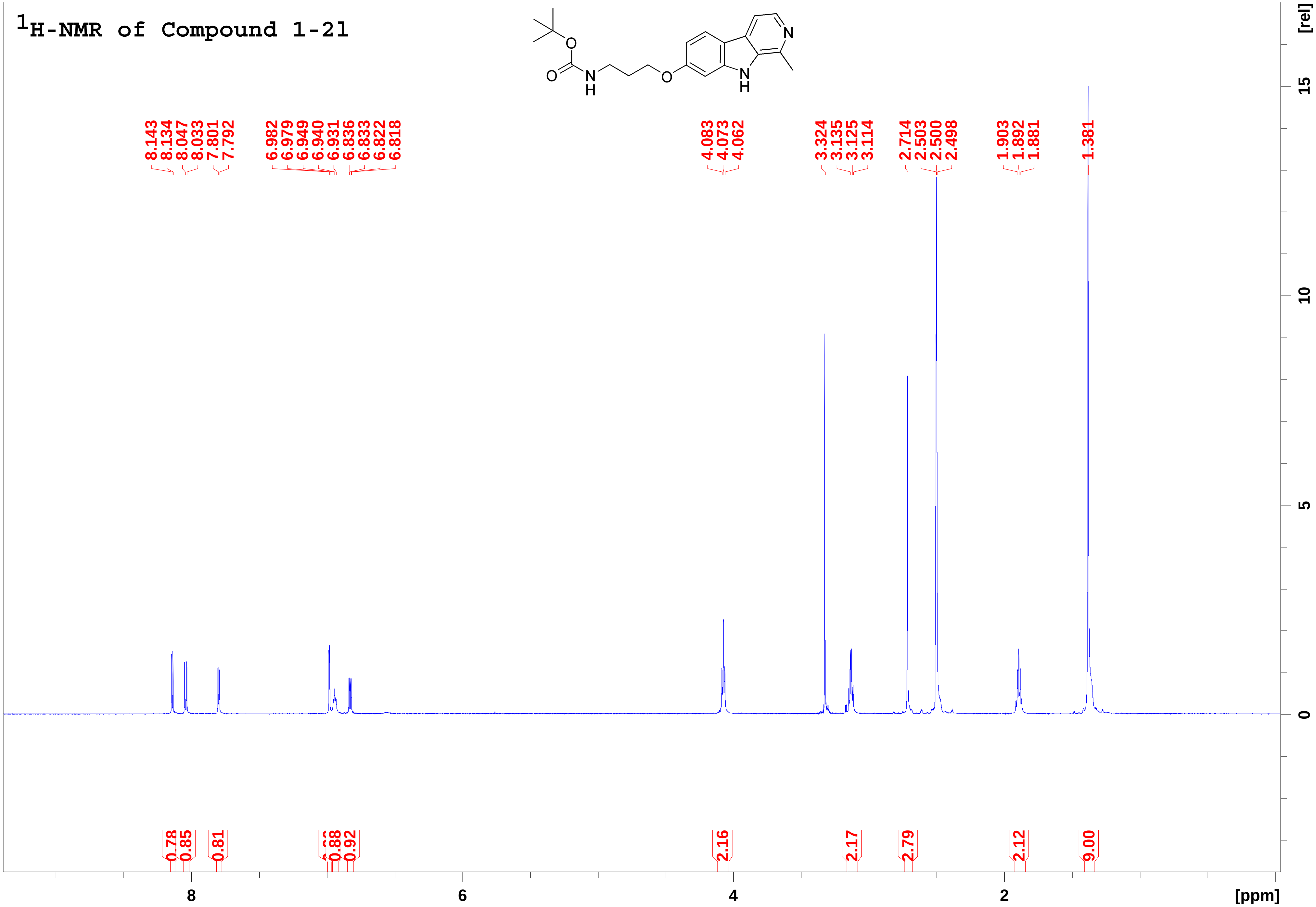

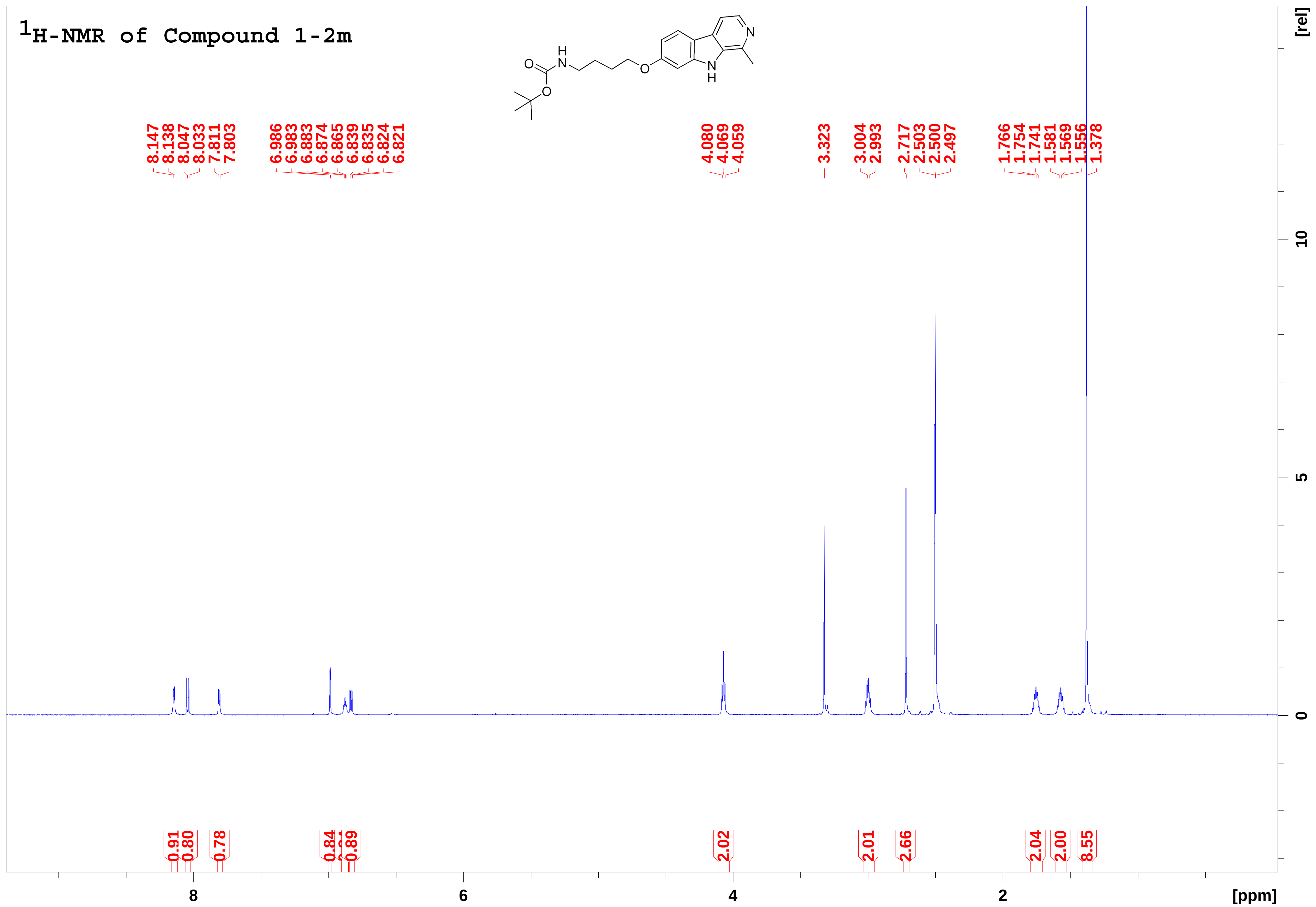

1H-NMR of Compound 1-2m
8.147
8.138
8.047
8.033
7.811
7.803
6.986
6.983
6.883
6.874
6.865
6.839
6.835
6.824
6.821
4.080
4.069
4.059
3.323
3.004
2.993
2.717
2.503
2.500
2.497
1.766
1.754
1.741
1.581
1.569
1.556
1.378
0.91
0.80
0.78
0.84
0.89
2.02
2.01
2.66
2.04
2.00
8.55
[rel]
10
5
0
8
6
4
2
[ppm]

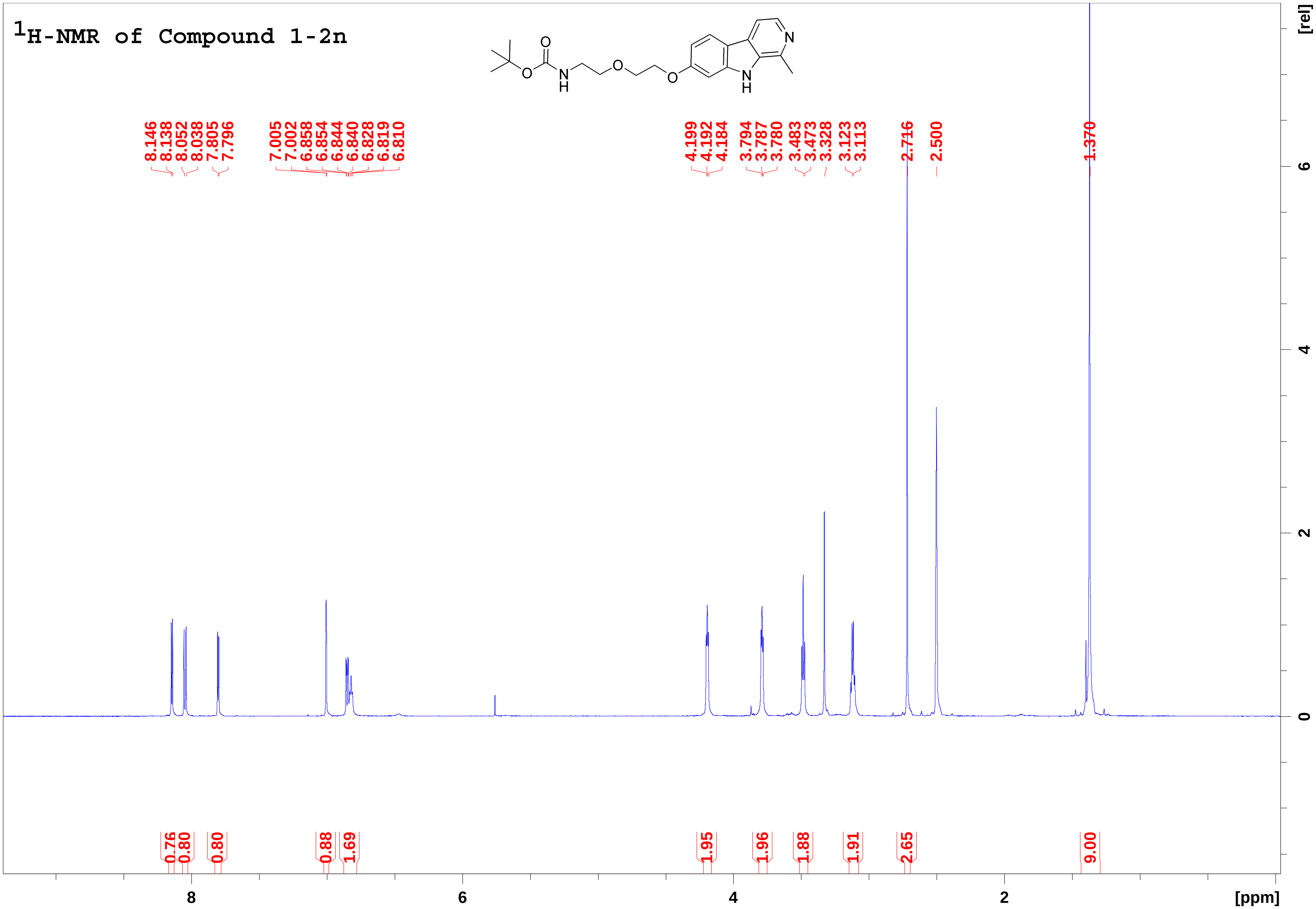

1H-NMR of Compound 1-2n
8.146 8.138 8.052 8.038 7.805 7.796
7.005 7.002 6.858 6.854 6.844 6.840 6.828 6.819 6.810
4.199 4.192 4.184 3.794 3.787 3.780 3.483 3.473 3.328 3.123 3.113
2.716
2.500
1.370
0.76 0.80 0.80 0.88 1.69 1.95 1.96 1.88 1.91 2.65 9.00
[rel]
[ppm]

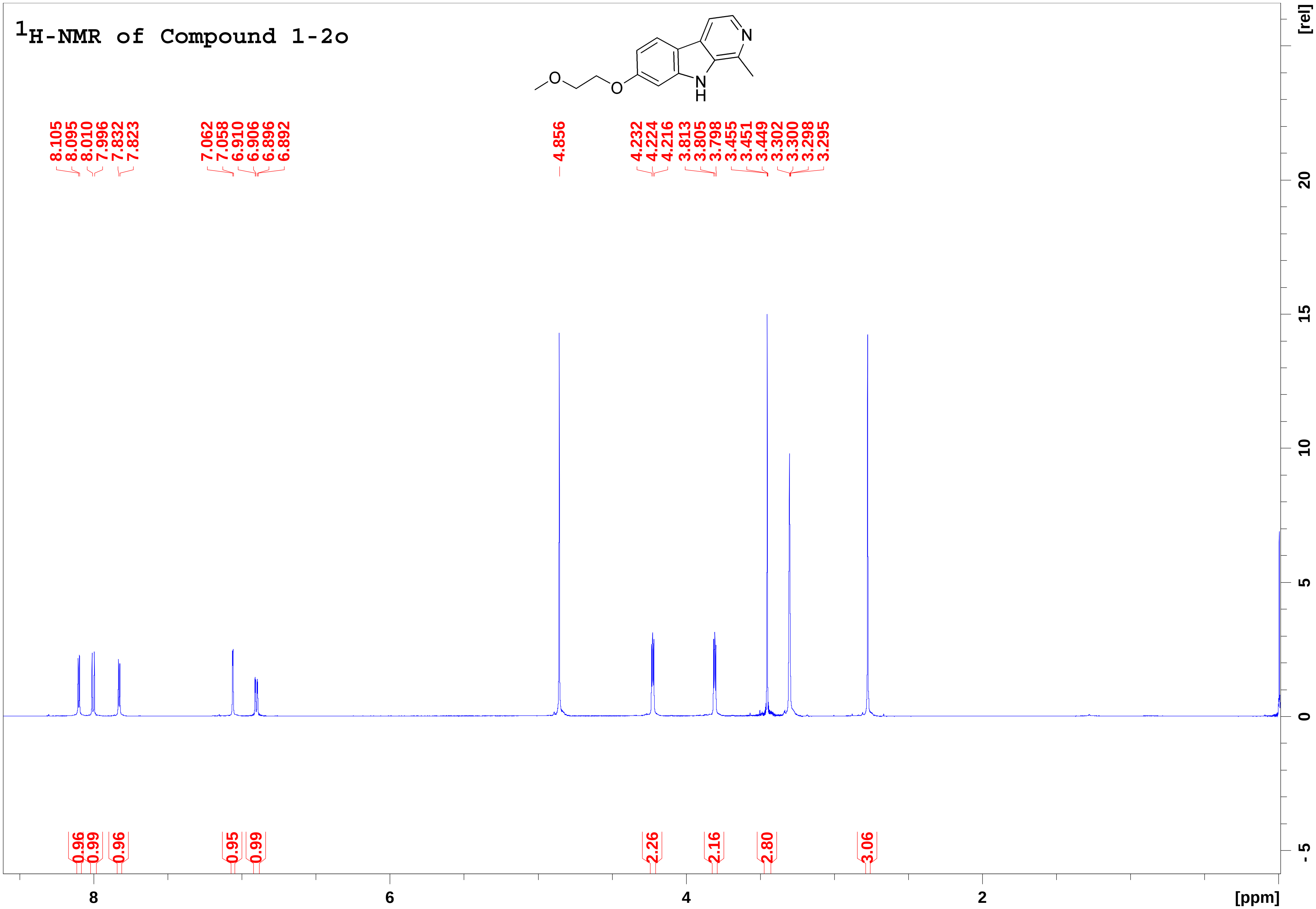

1H-NMR of Compound 1-2o
8.105
8.095
8.010
7.996
7.832
7.823
7.062
7.058
6.910
6.906
6.896
6.892
4.856
4.232
4.224
4.216
3.813
3.805
3.798
3.455
3.451
3.449
3.302
3.300
3.298
3.295
0.96
0.99
0.96
0.95
0.99
2.26
2.16
2.80
3.06
[rel]
[ppm]

^{1}H-NMR of Compound 1-2p

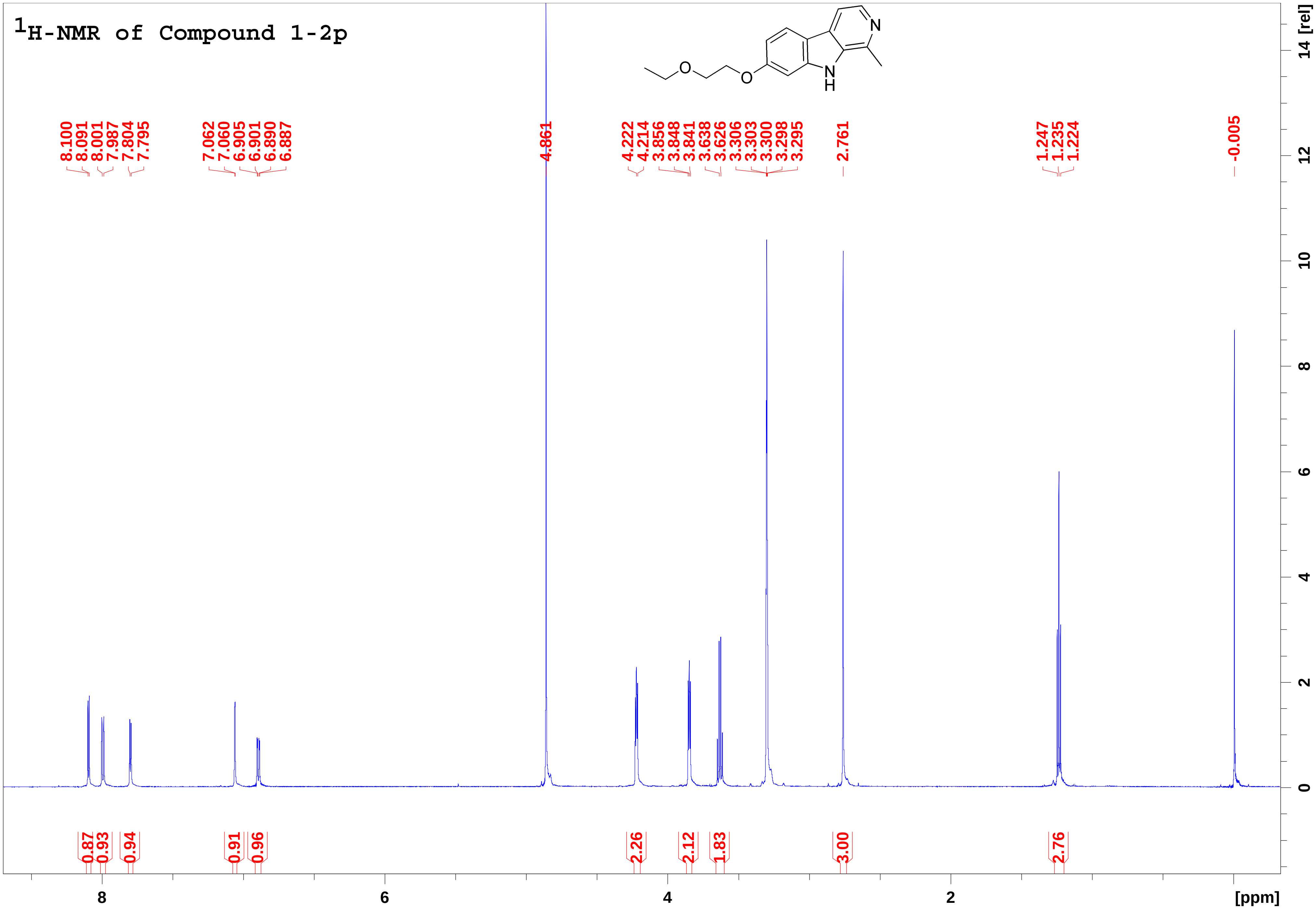

^{1}H-NMR of Compound 1-3a

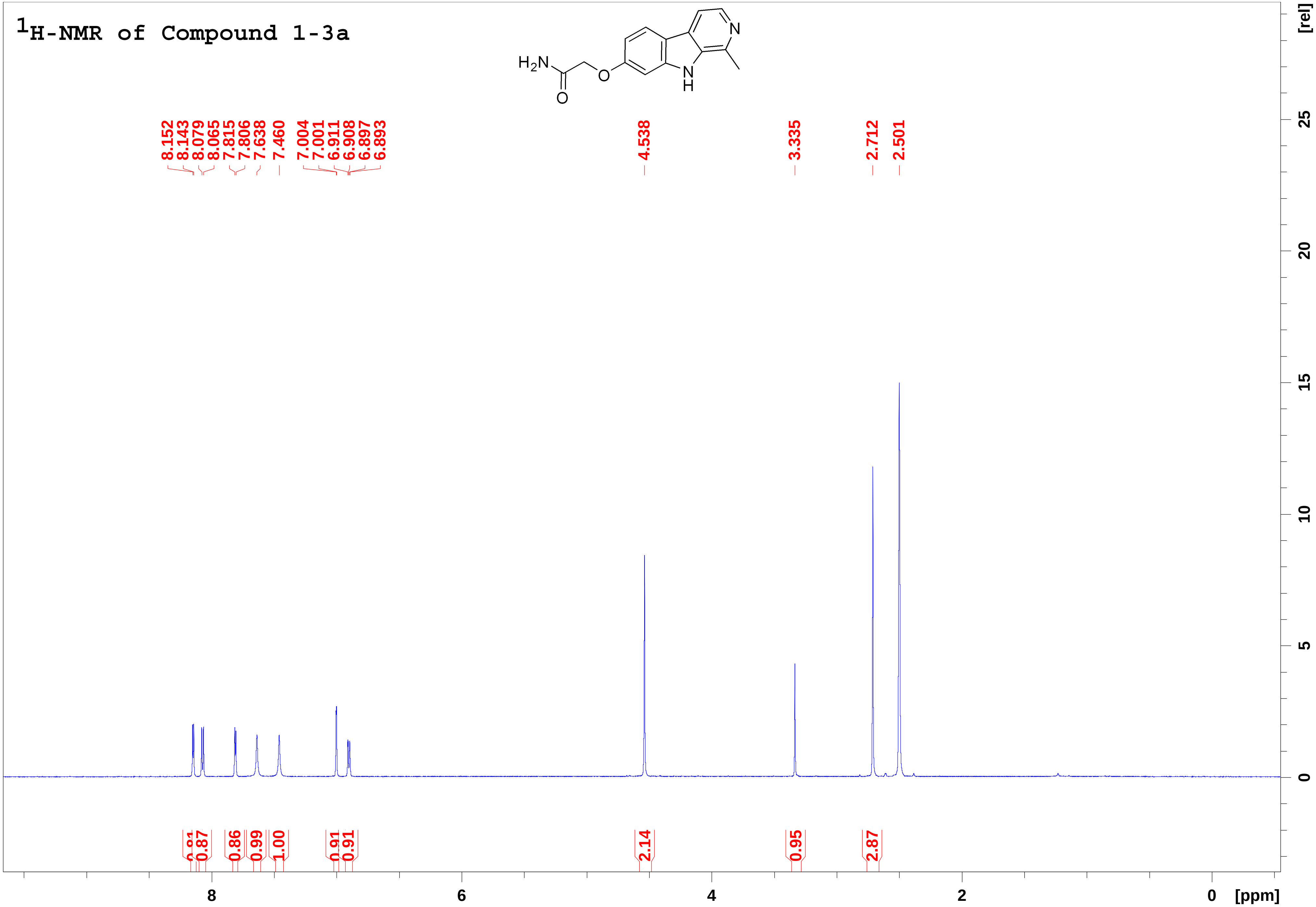

^{1}H-NMR of Compound 1-3b

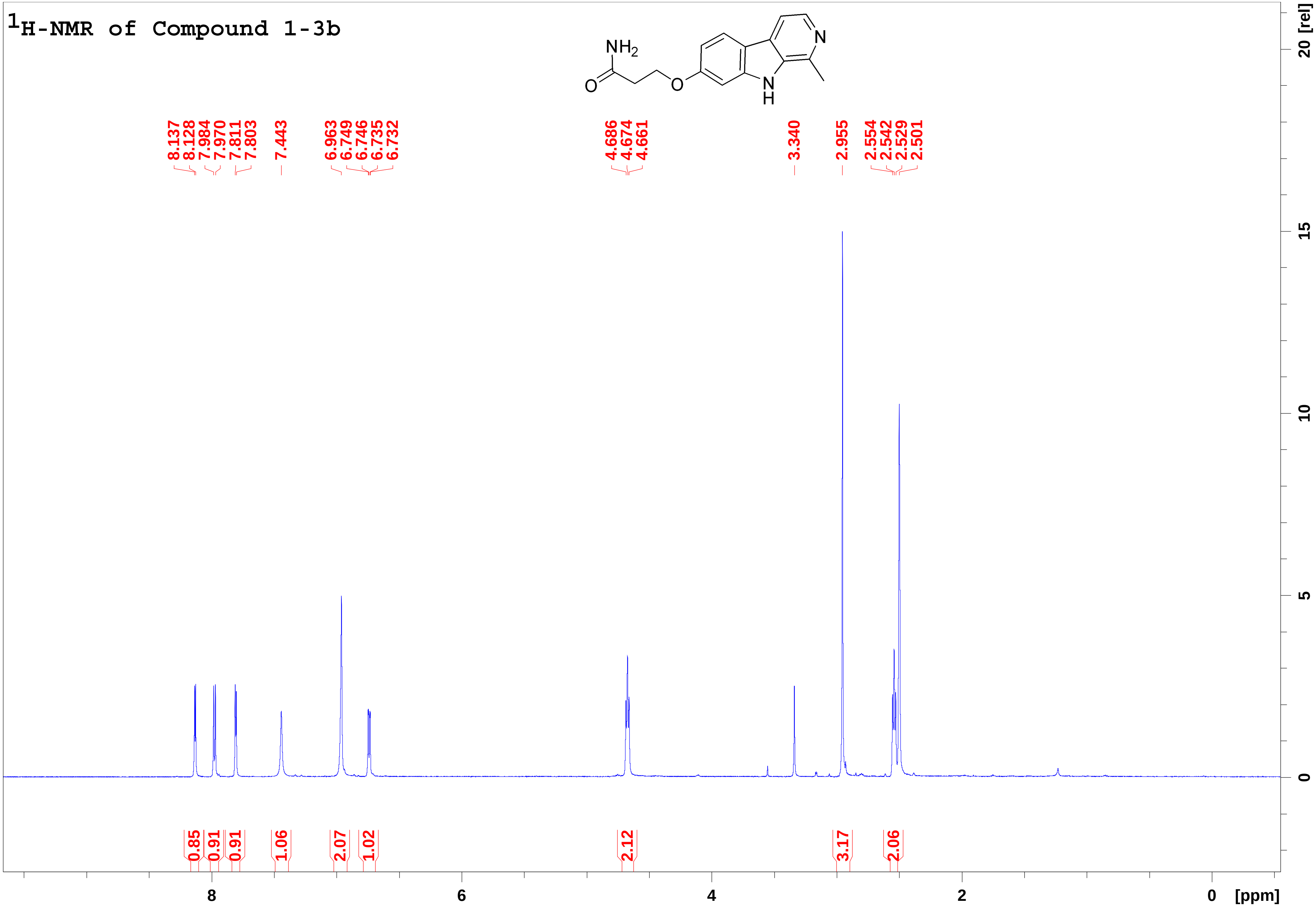

^{1}H-NMR of Compound 1-3c

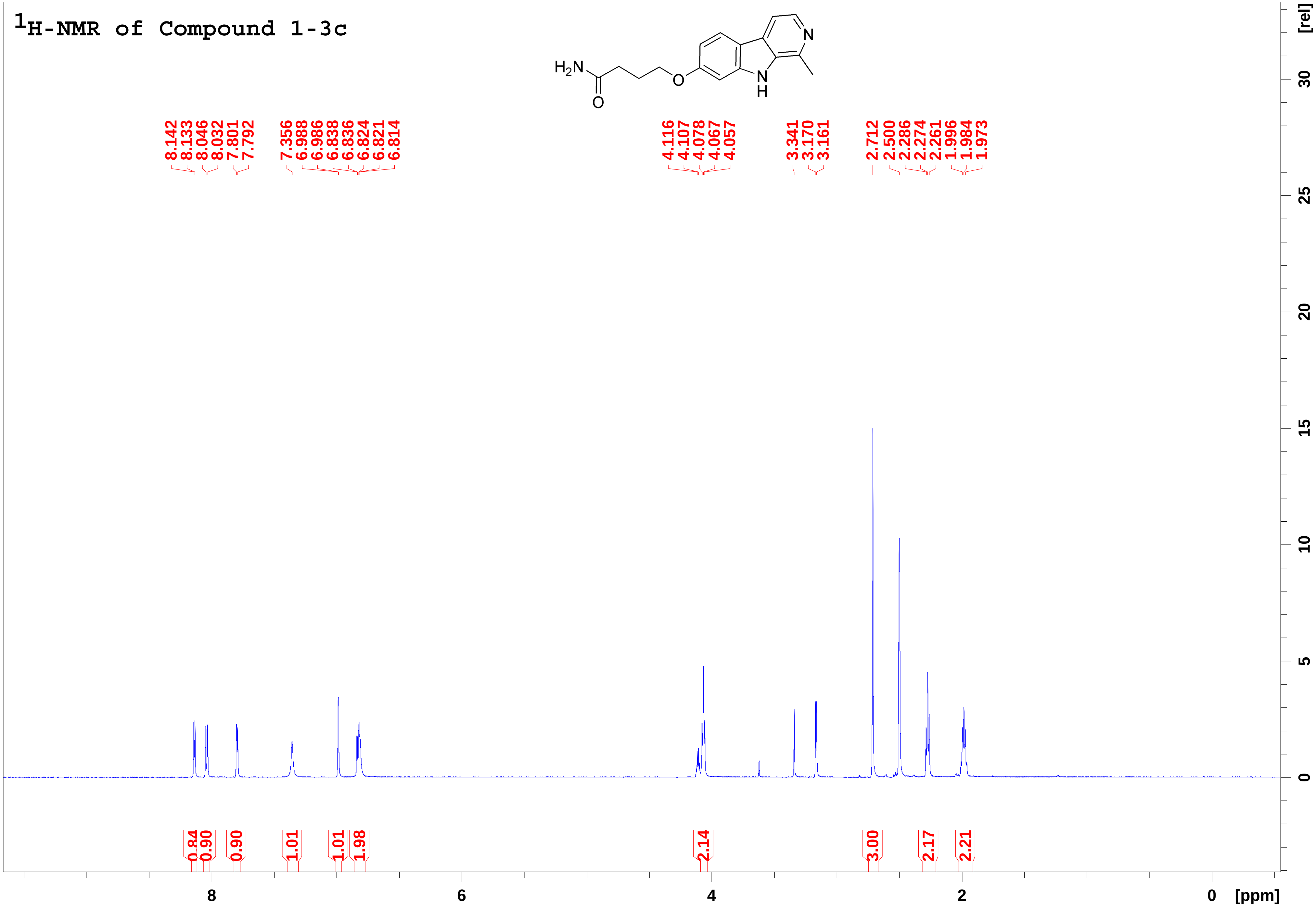

^{1}H-NMR of Compound 1-3d

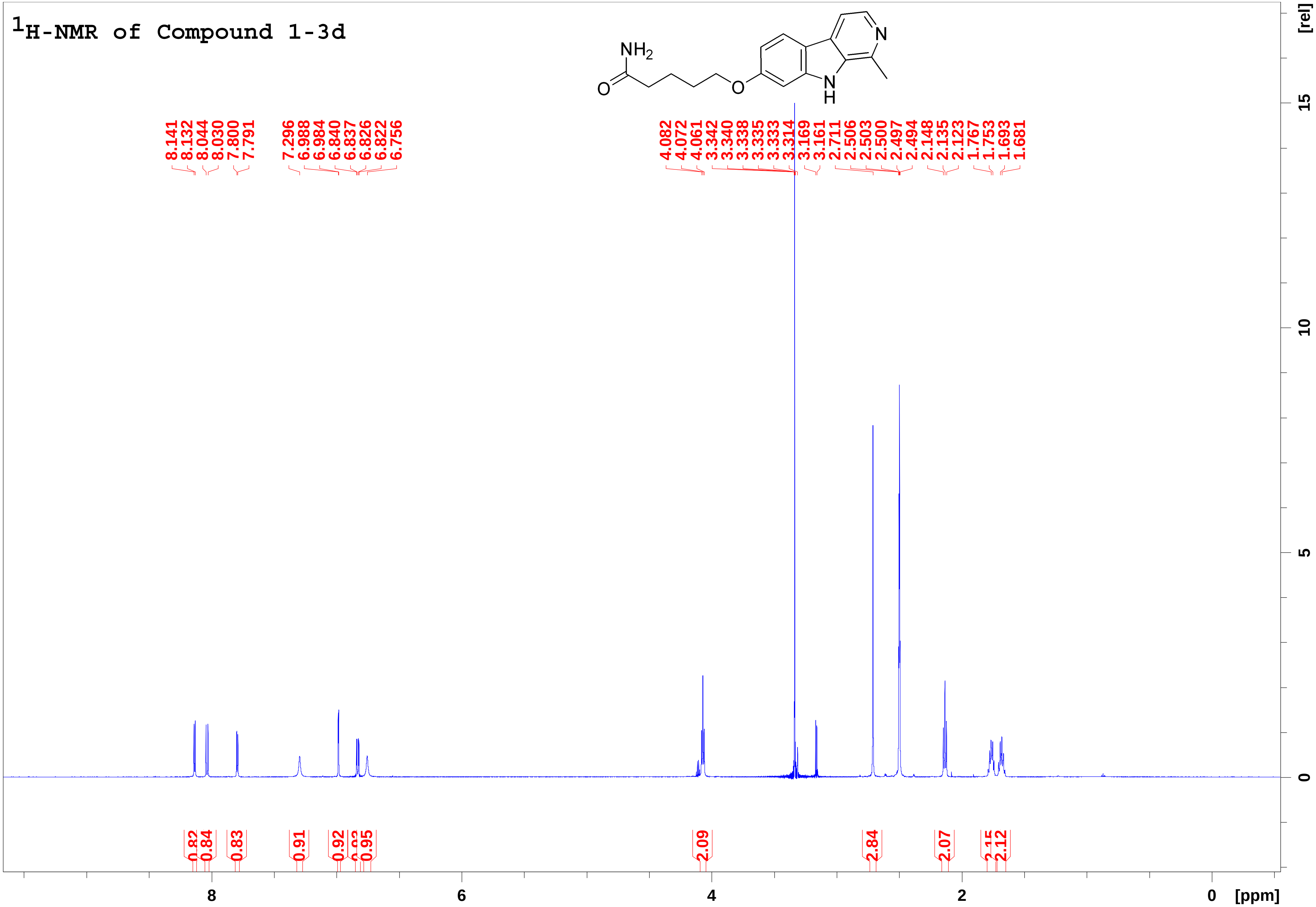

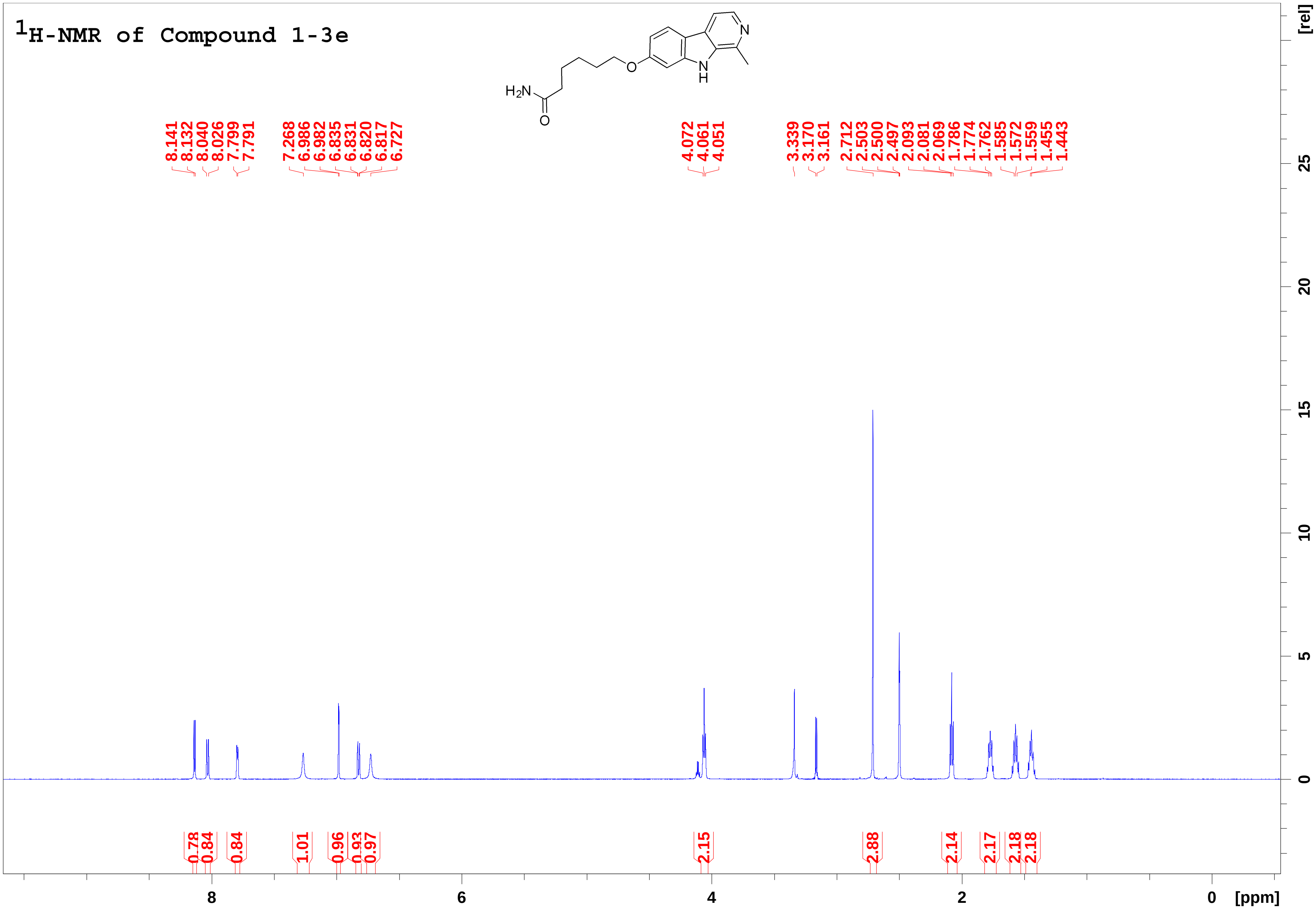
1H-NMR of Compound 1-3e
H2N
O
N
H
N
8.141
8.132
8.040
8.026
7.799
7.791
7.268
6.986
6.982
6.835
6.831
6.820
6.817
6.727
4.072
4.061
4.051
3.339
3.170
3.161
2.712
2.503
2.500
2.497
2.093
2.081
2.069
1.786
1.774
1.762
1.585
1.572
1.559
1.455
1.443
0.78
0.84
0.84
1.01
0.96
0.93
0.97
2.15
2.88
2.14
2.17
2.18
2.18
[rel]
[ppm]

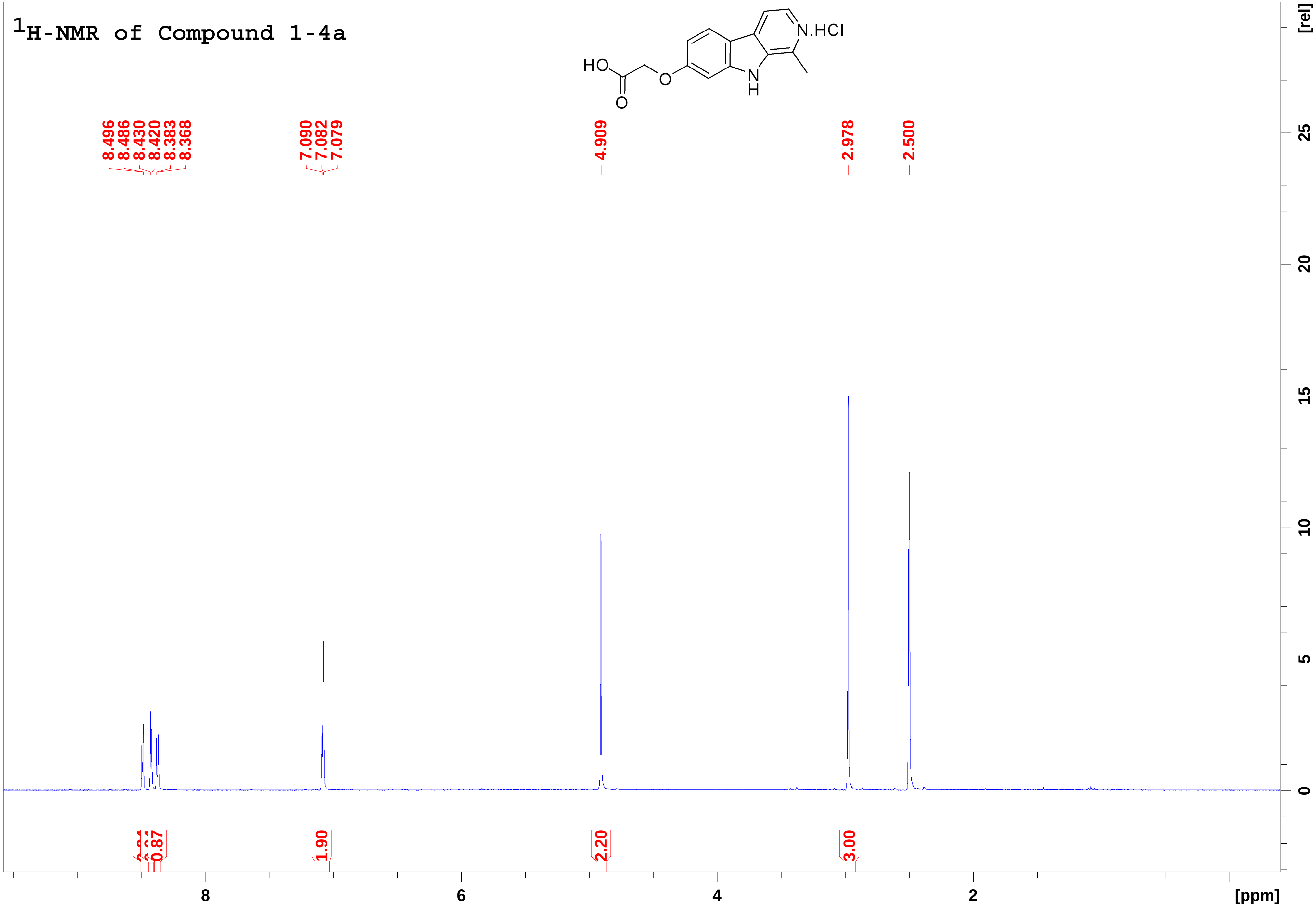

1H-NMR of Compound 1-4a
N.HCl
HO
O
O
N
H
8.496
8.486
8.430
8.420
8.383
8.368
7.090
7.082
7.079
4.909
2.978
2.500
0.87
1.90
2.20
3.00
[rel]
[ppm]

1H-NMR of Compound 1-4b

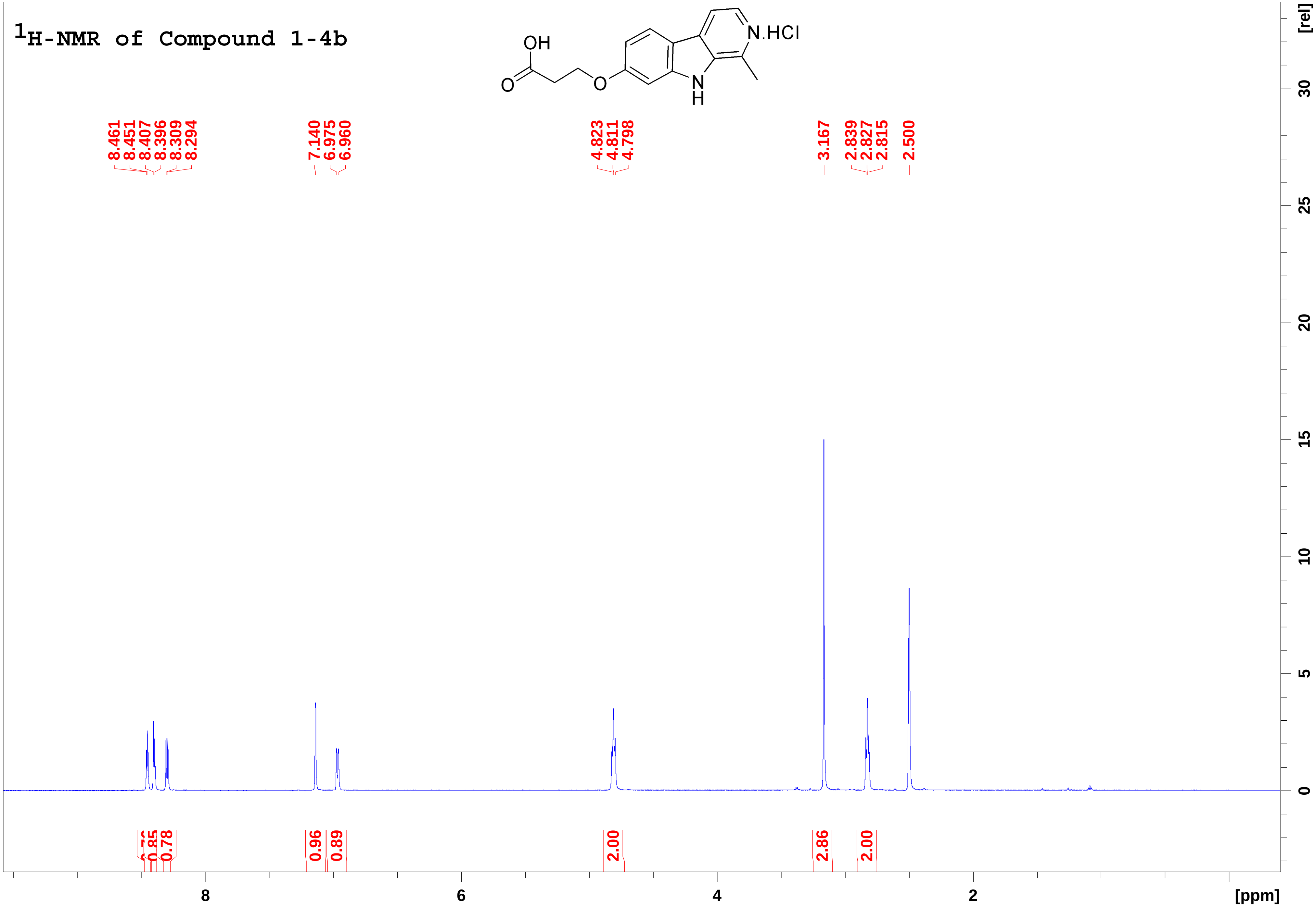

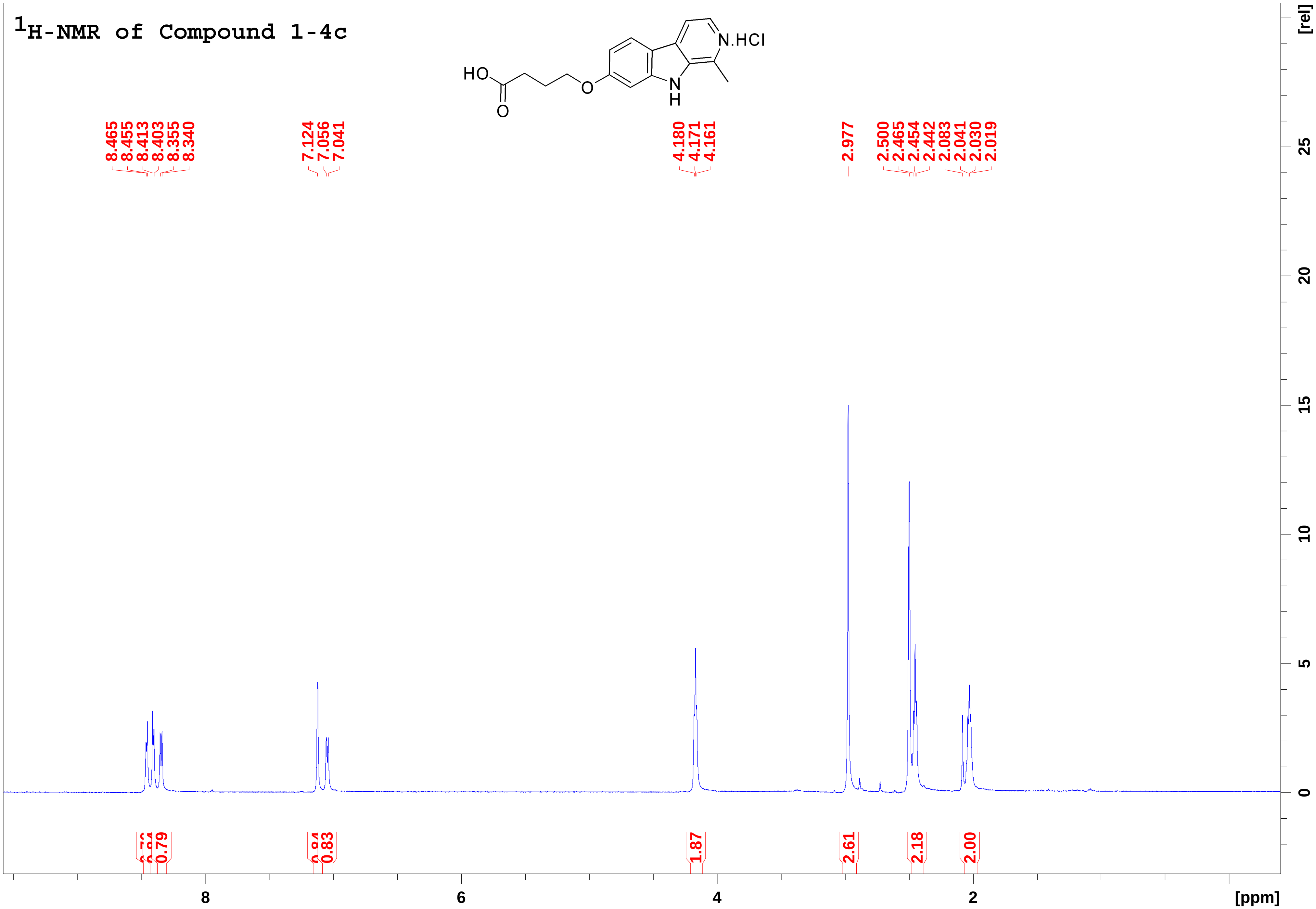

1H-NMR of Compound 1-4c
HO
O
O
N
H
N.HCl
8.465
8.455
8.413
8.403
8.355
8.340
7.124
7.056
7.041
4.180
4.171
4.161
2.977
2.500
2.465
2.454
2.442
2.083
2.041
2.030
2.019
0.79
0.83
1.87
2.61
2.18
2.00
[rel]
25
20
15
10
5
0
8
6
4
2
[ppm]

^{1}H-NMR of Compound 1-4d

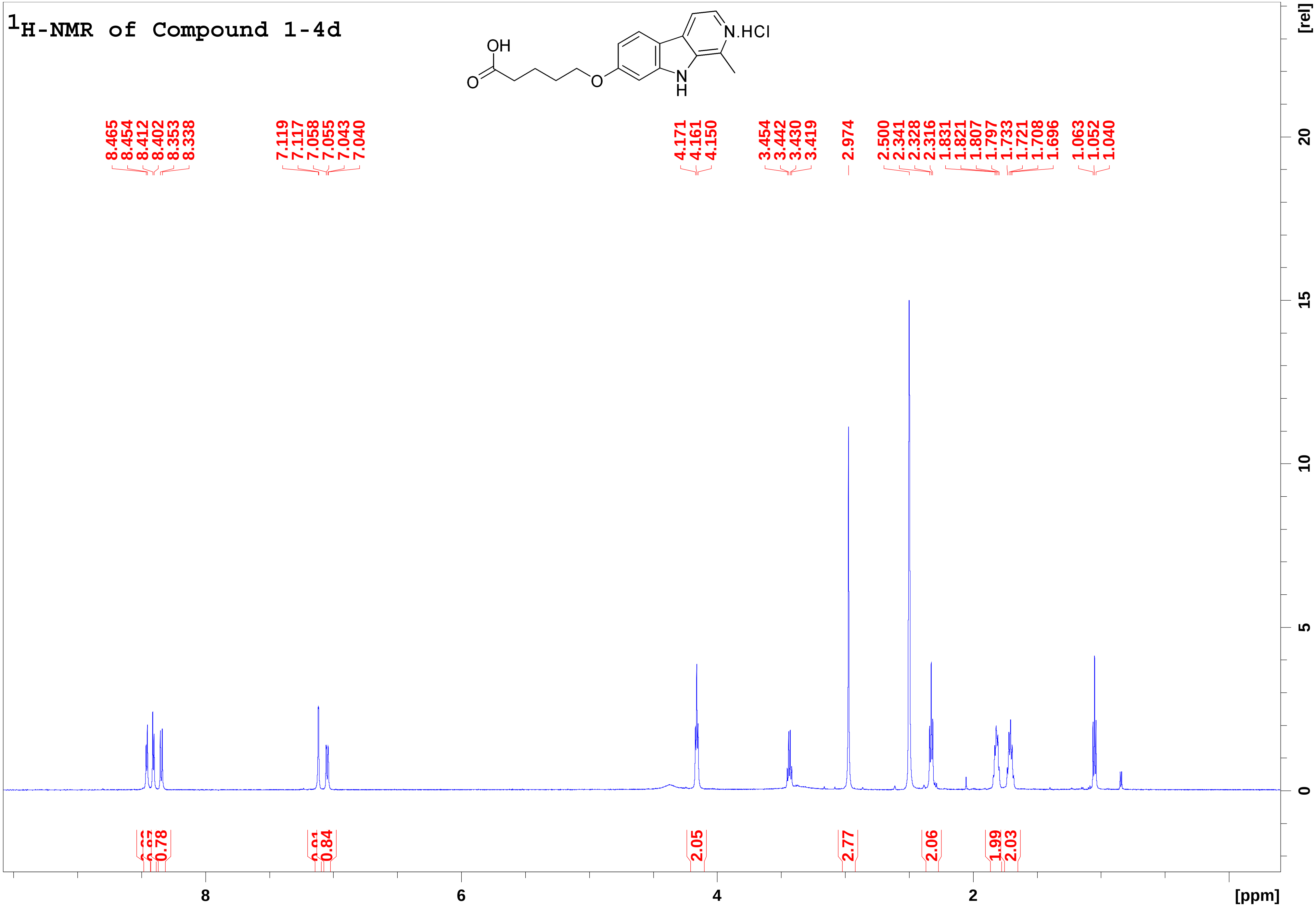

^{1}H-NMR of Compound 1-4e

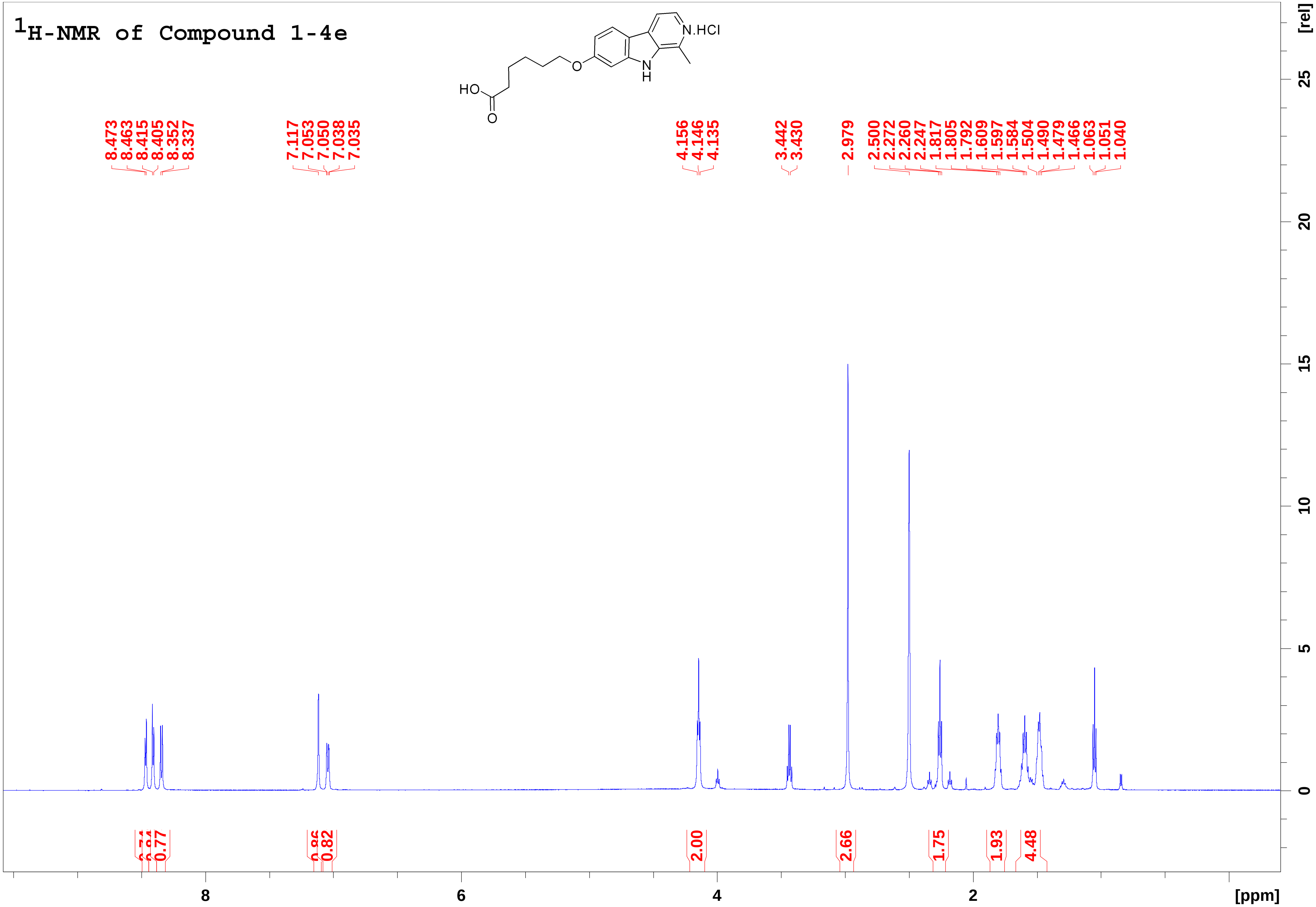

^{1}H-NMR of Compound 1-5a

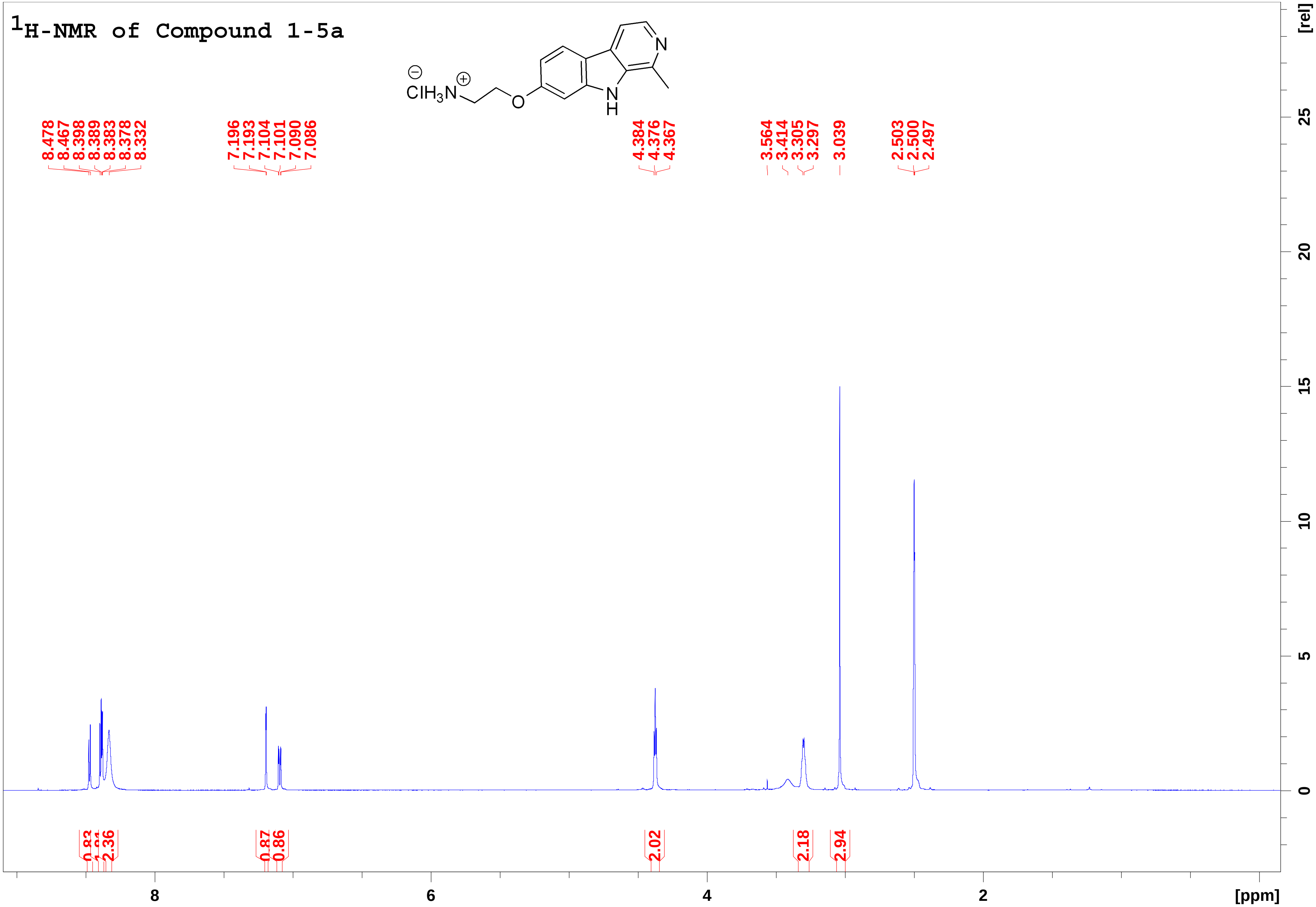

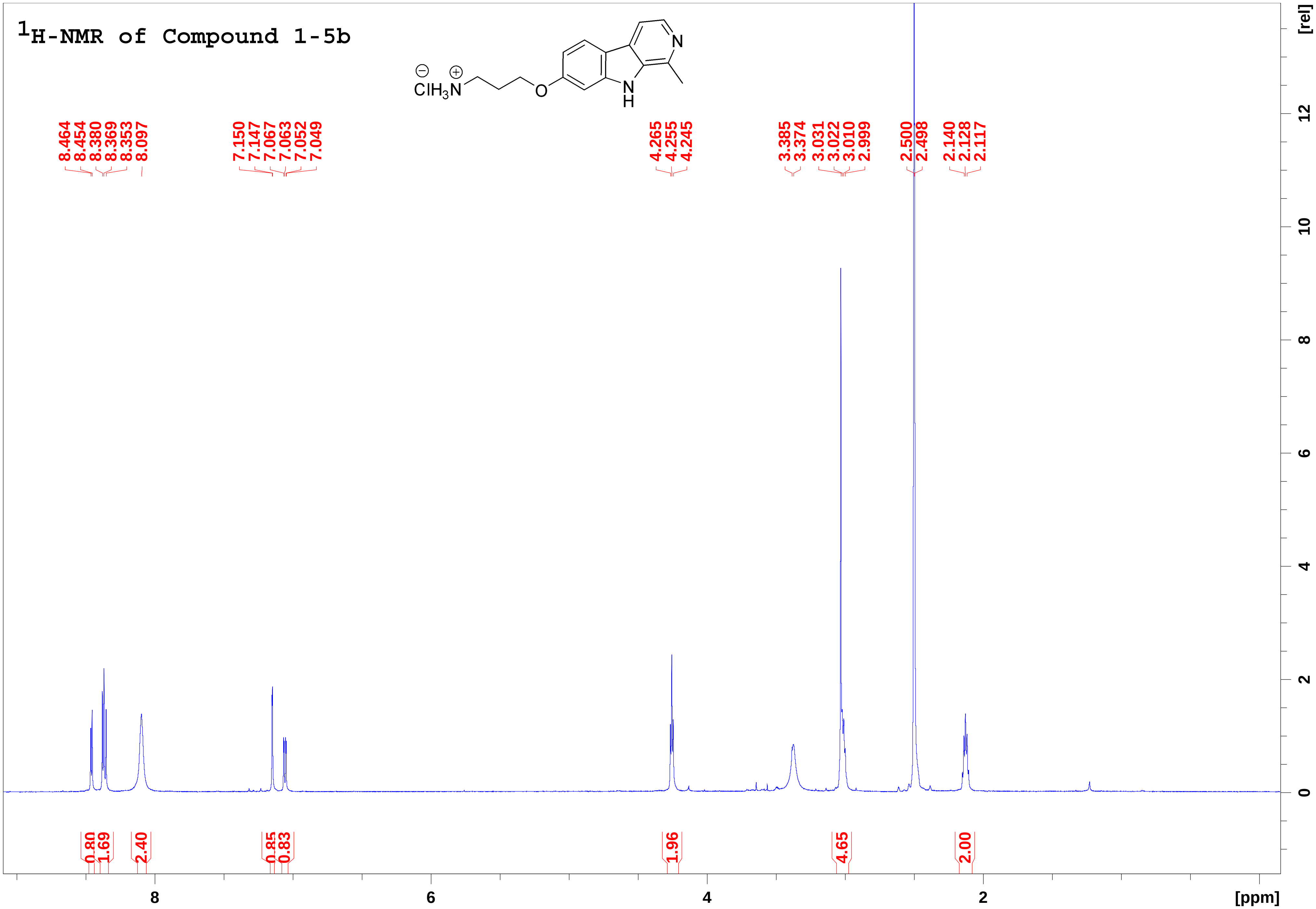

1H-NMR of Compound 1-5b
ClH3N
O
N
H
N
8.464
8.454
8.380
8.369
8.353
8.097
7.150
7.147
7.067
7.063
7.052
7.049
4.265
4.255
4.245
3.385
3.374
3.031
3.022
3.010
2.999
2.500
2.498
2.140
2.128
2.117
0.80
1.69
2.40
0.85
0.83
1.96
4.65
2.00
[rel]
[ppm]

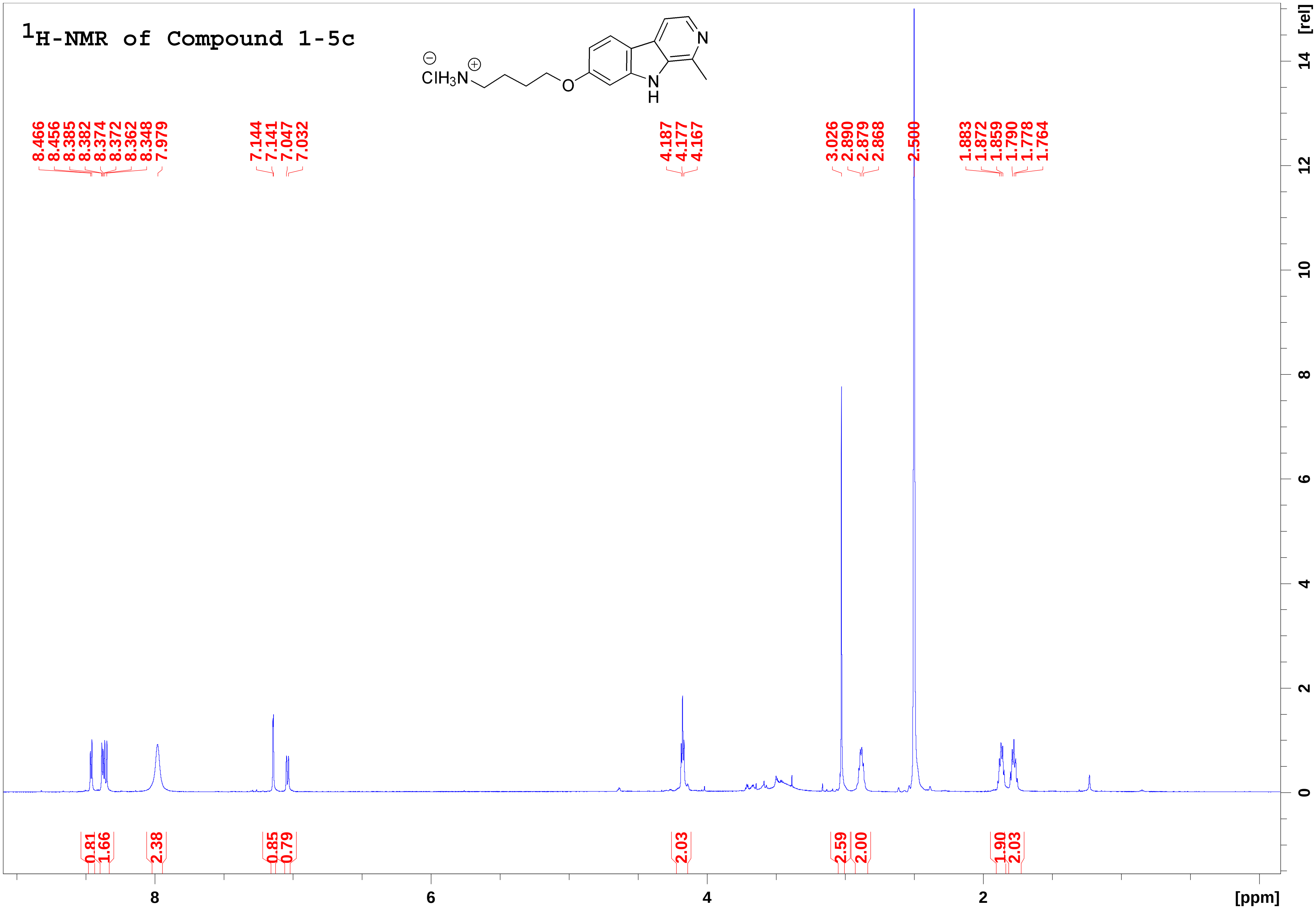
1H-NMR of Compound 1-5c
ClH3N
O
N
H
N
8.466
8.456
8.385
8.382
8.374
8.372
8.362
8.348
7.979
7.144
7.141
7.047
7.032
4.187
4.177
4.167
3.026
2.890
2.879
2.868
2.500
1.883
1.872
1.859
1.790
1.778
1.764
0.81
1.66
2.38
0.85
0.79
2.03
2.59
2.00
1.90
2.03
[rel]
[ppm]

^{1}H-NMR of Compound 1-5d

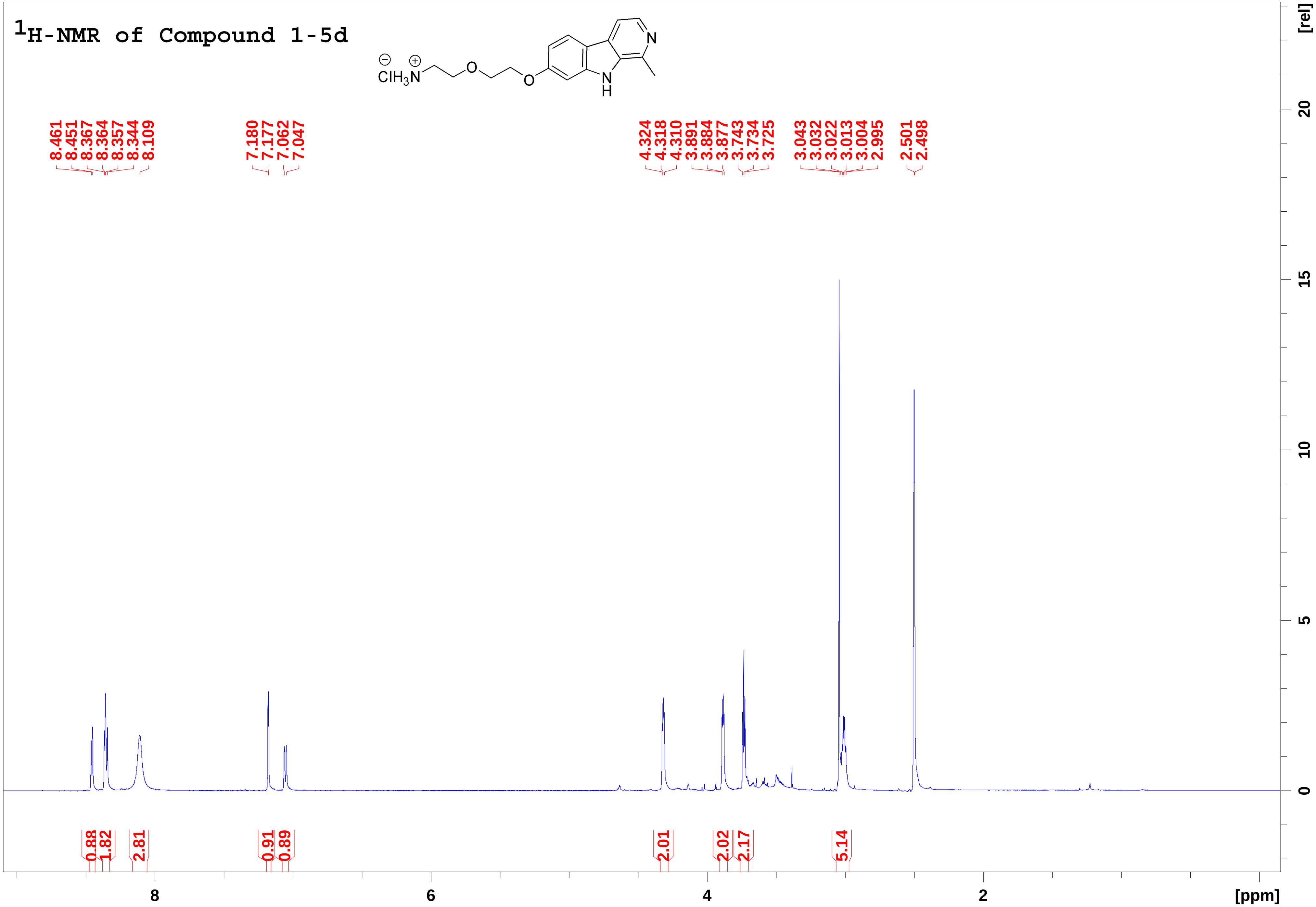

^{1}H-NMR of Compound 1-6a

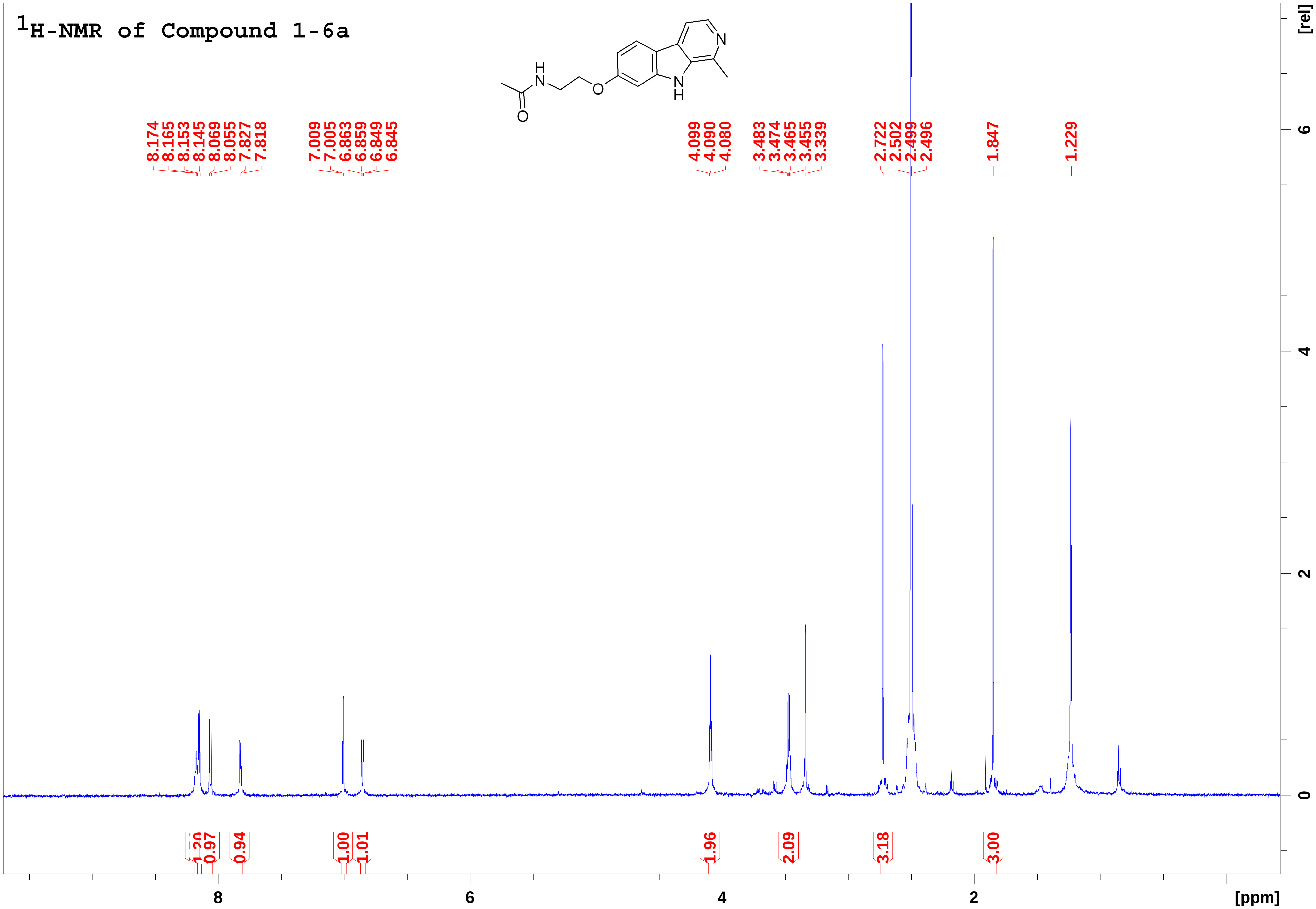

^{1}H-NMR of Compound 1-6b

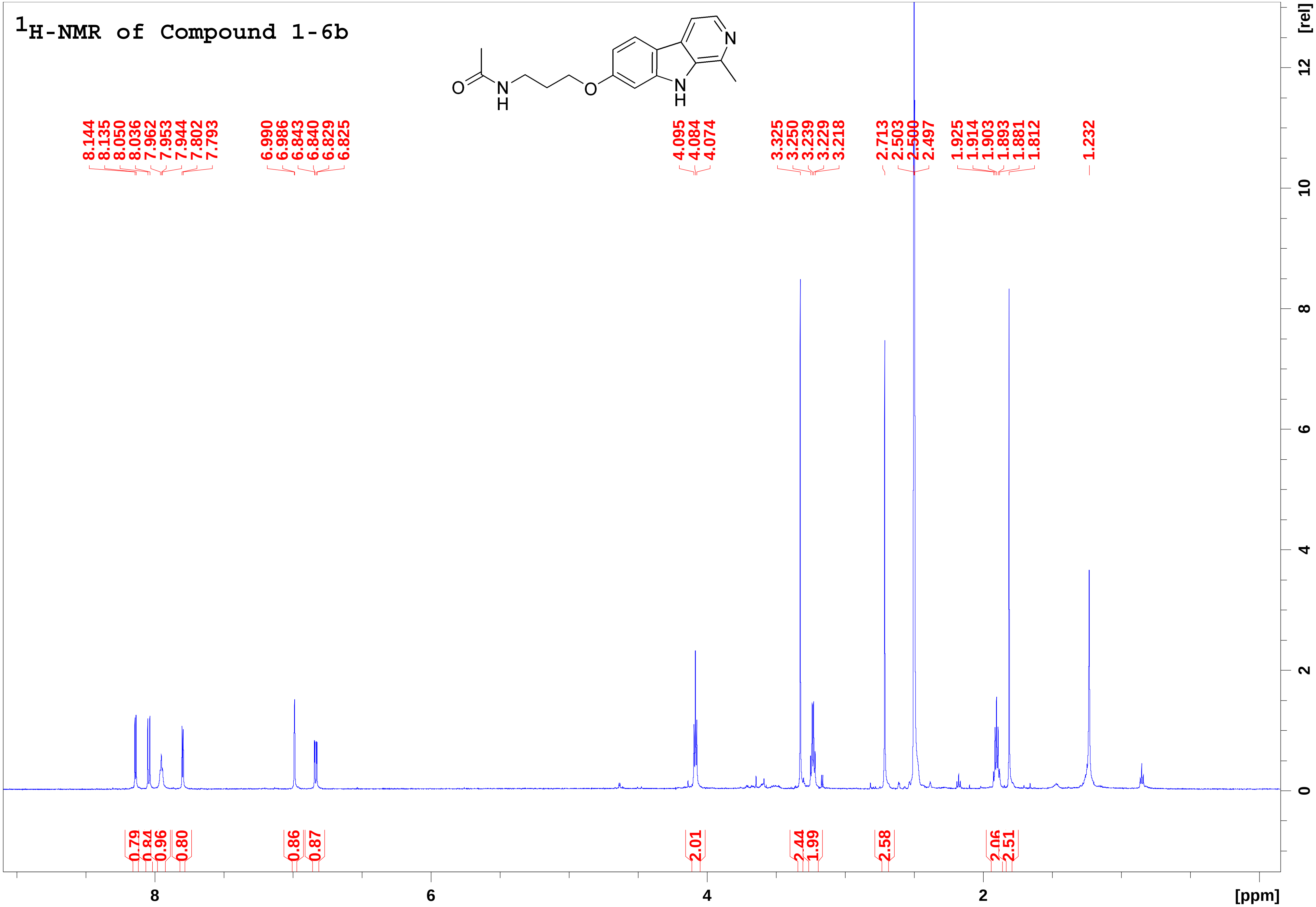

^{1}H-NMR of Compound 1-6c

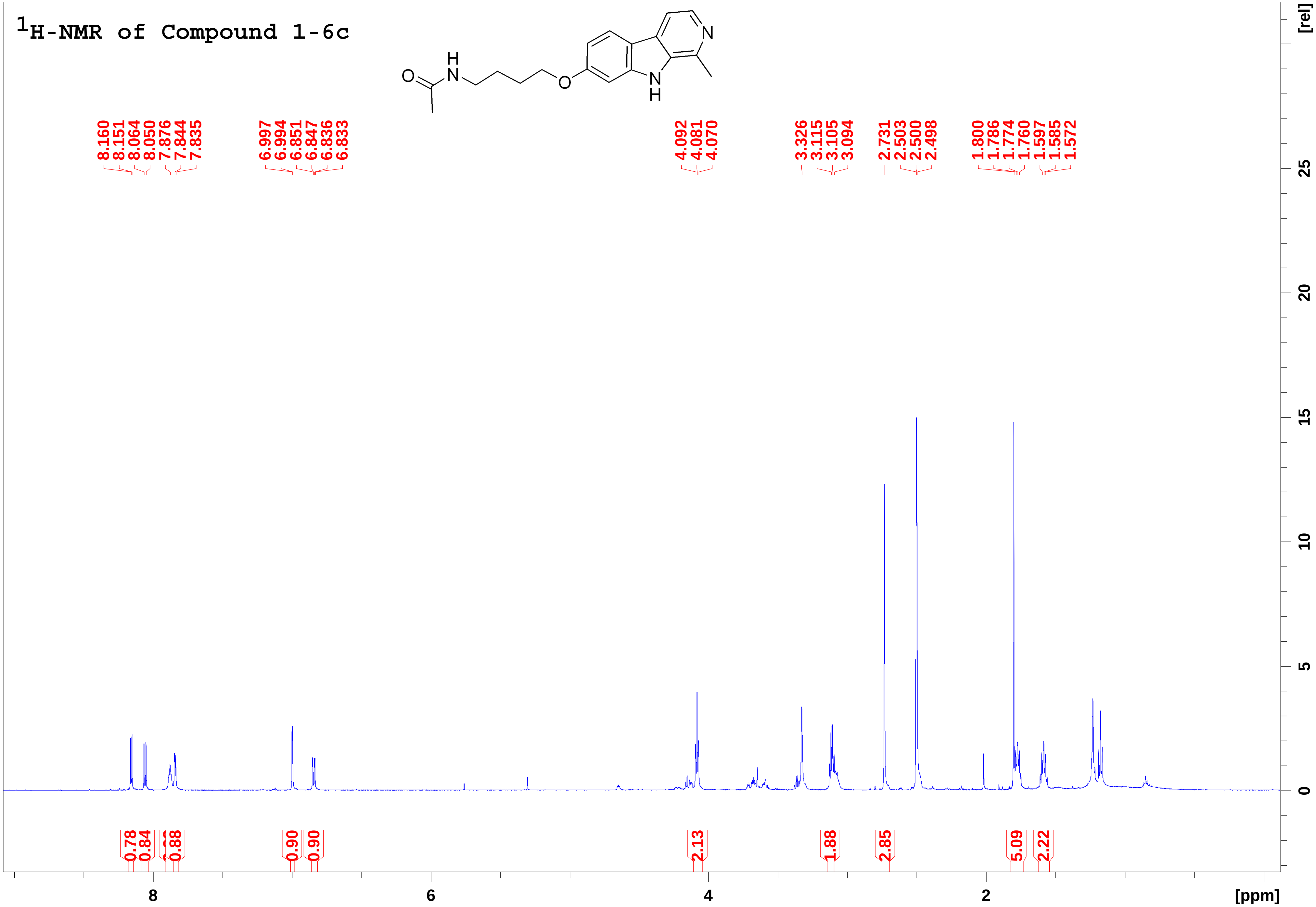

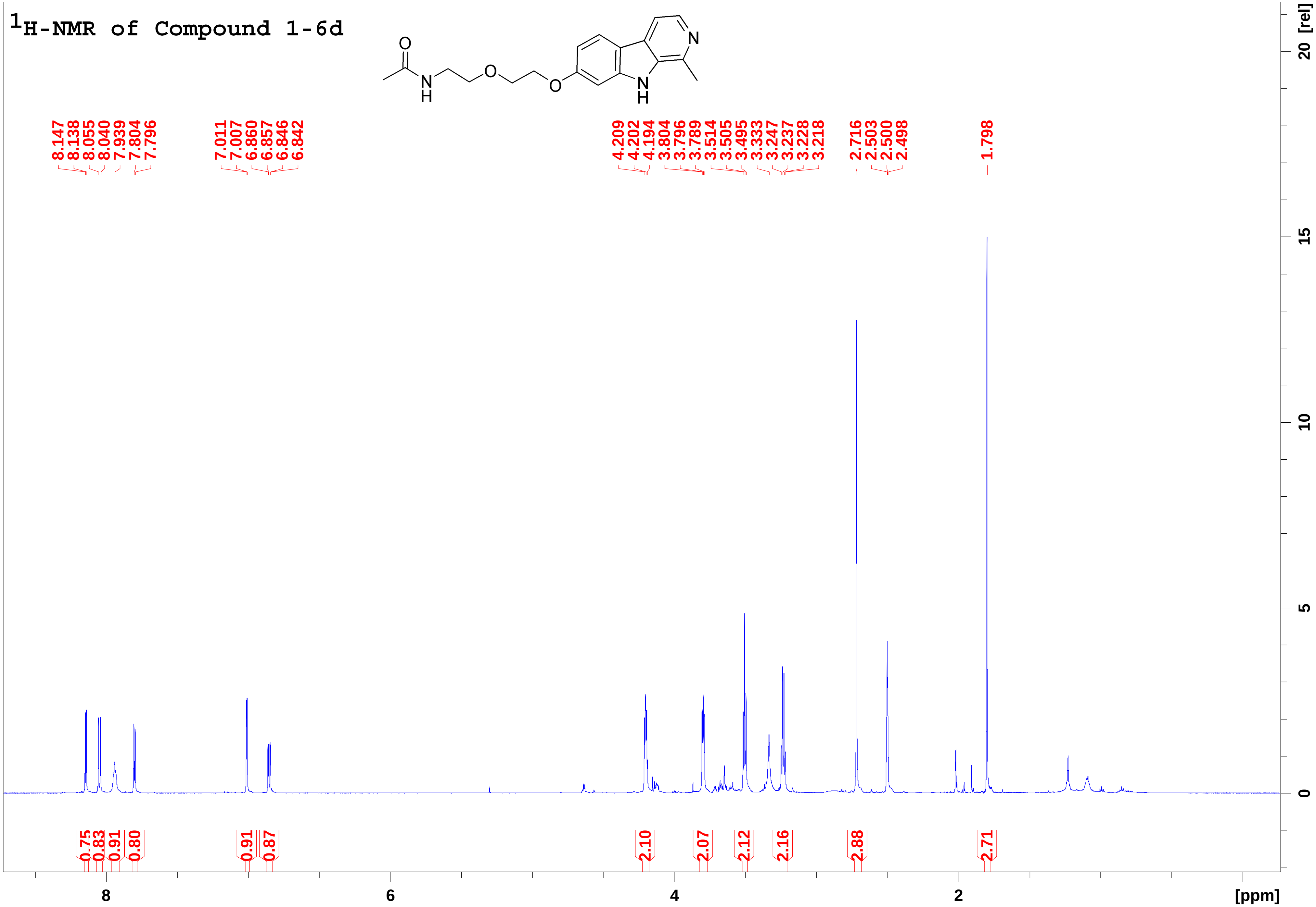
1H-NMR of Compound 1-6d
O
N
H
O
O
N
H
N
8.147
8.138
8.055
8.040
7.939
7.804
7.796
7.011
7.007
6.860
6.857
6.846
6.842
4.209
4.202
4.194
3.804
3.796
3.789
3.514
3.505
3.495
3.333
3.247
3.237
3.228
3.218
2.716
2.503
2.500
2.498
1.798
0.75
0.83
0.91
0.80
0.91
0.87
2.10
2.07
2.12
2.16
2.88
2.71
[rel]
20
15
10
5
0
8
6
4
2
[ppm]

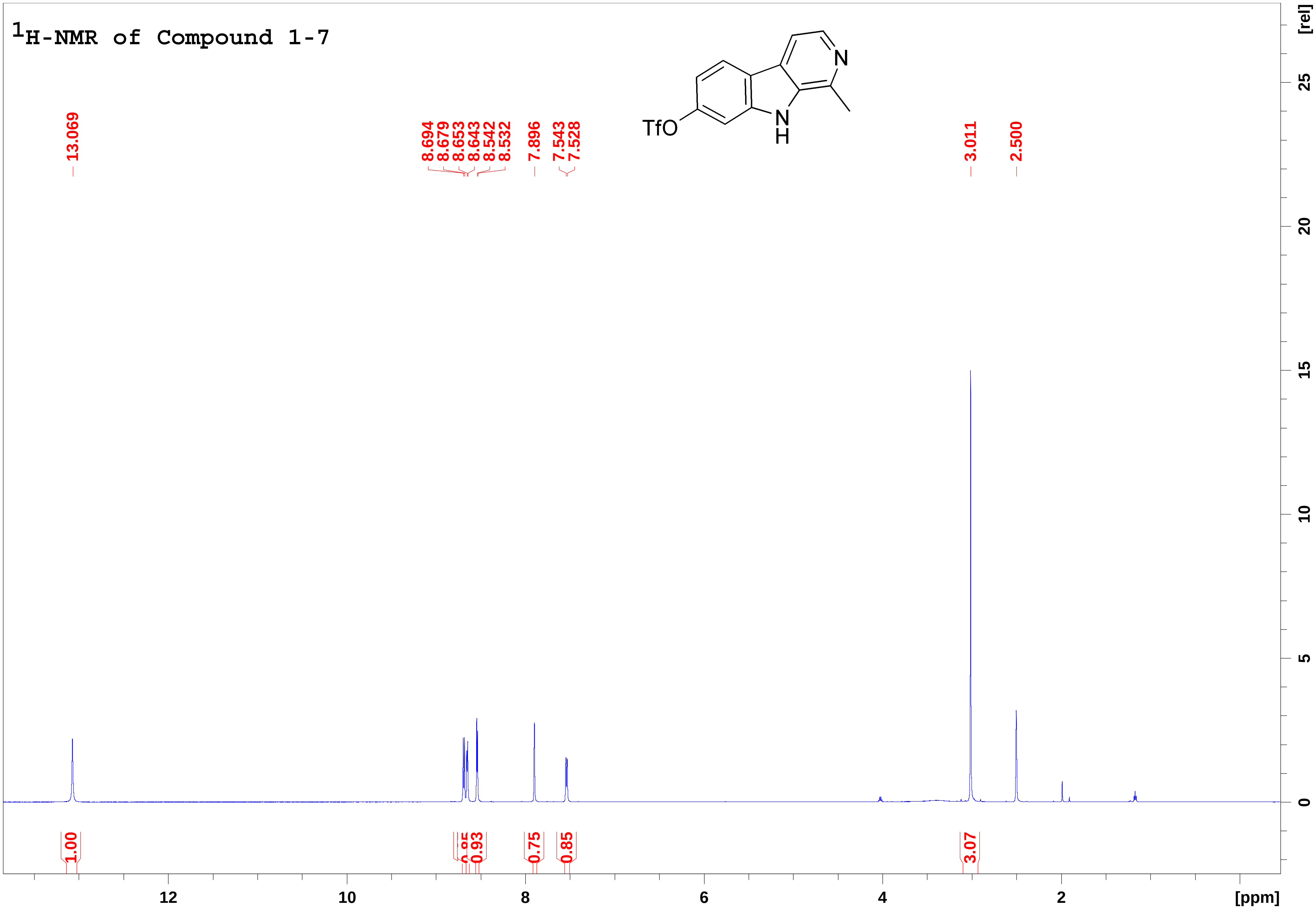

1H-NMR of Compound 1-7
TfO
N
N
H
13.069
8.694
8.679
8.653
8.643
8.542
8.532
7.896
7.543
7.528
3.011
2.500
1.00
0.93
0.75
0.85
3.07
[rel]
[ppm]

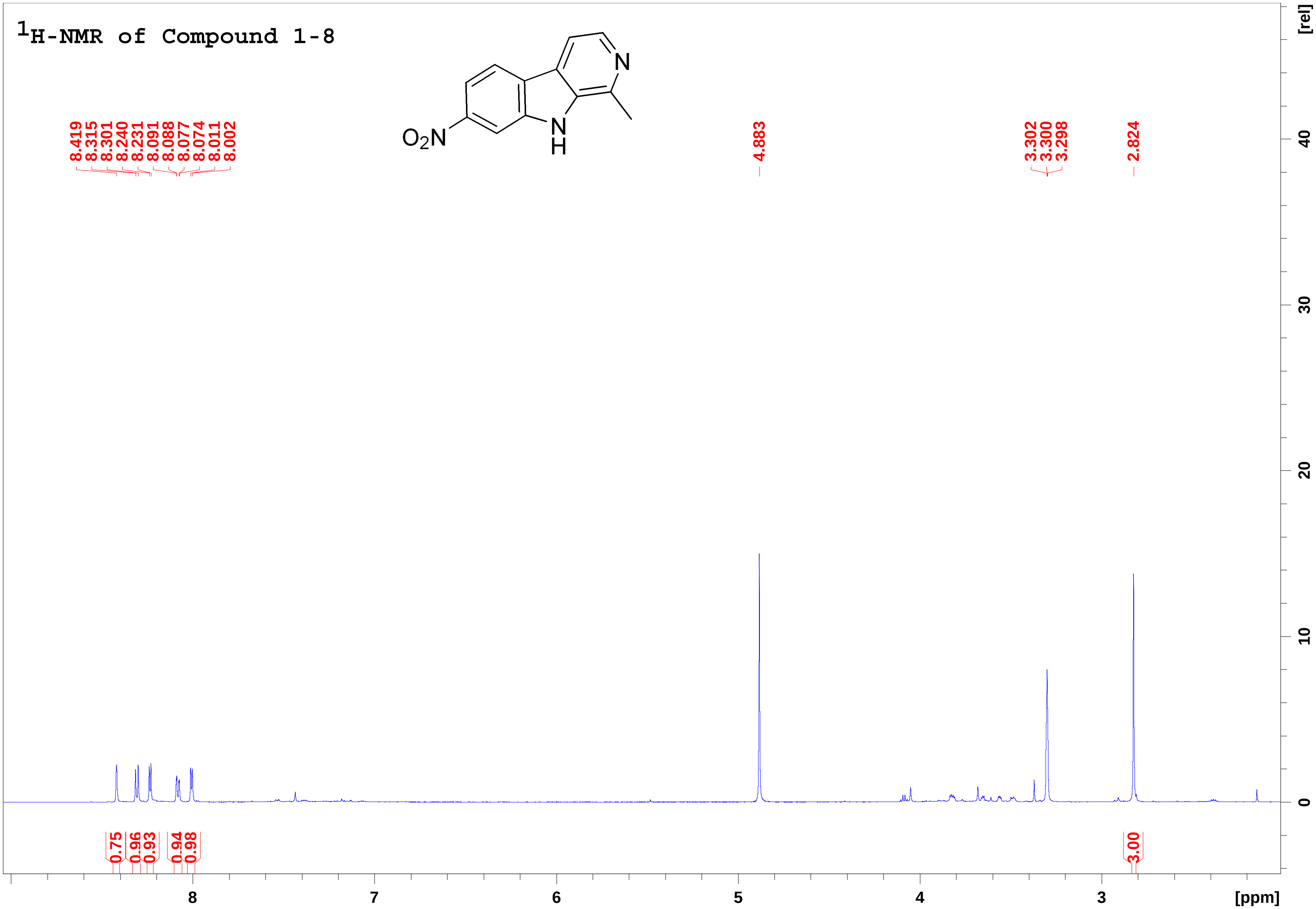

1H-NMR of Compound 1-8
O2N
N
H
N
8.419
8.315
8.301
8.240
8.231
8.091
8.088
8.077
8.074
8.011
8.002
4.883
3.302
3.300
3.298
2.824
0.75
0.96
0.93
0.94
0.98
3.00
[rel]
[ppm]

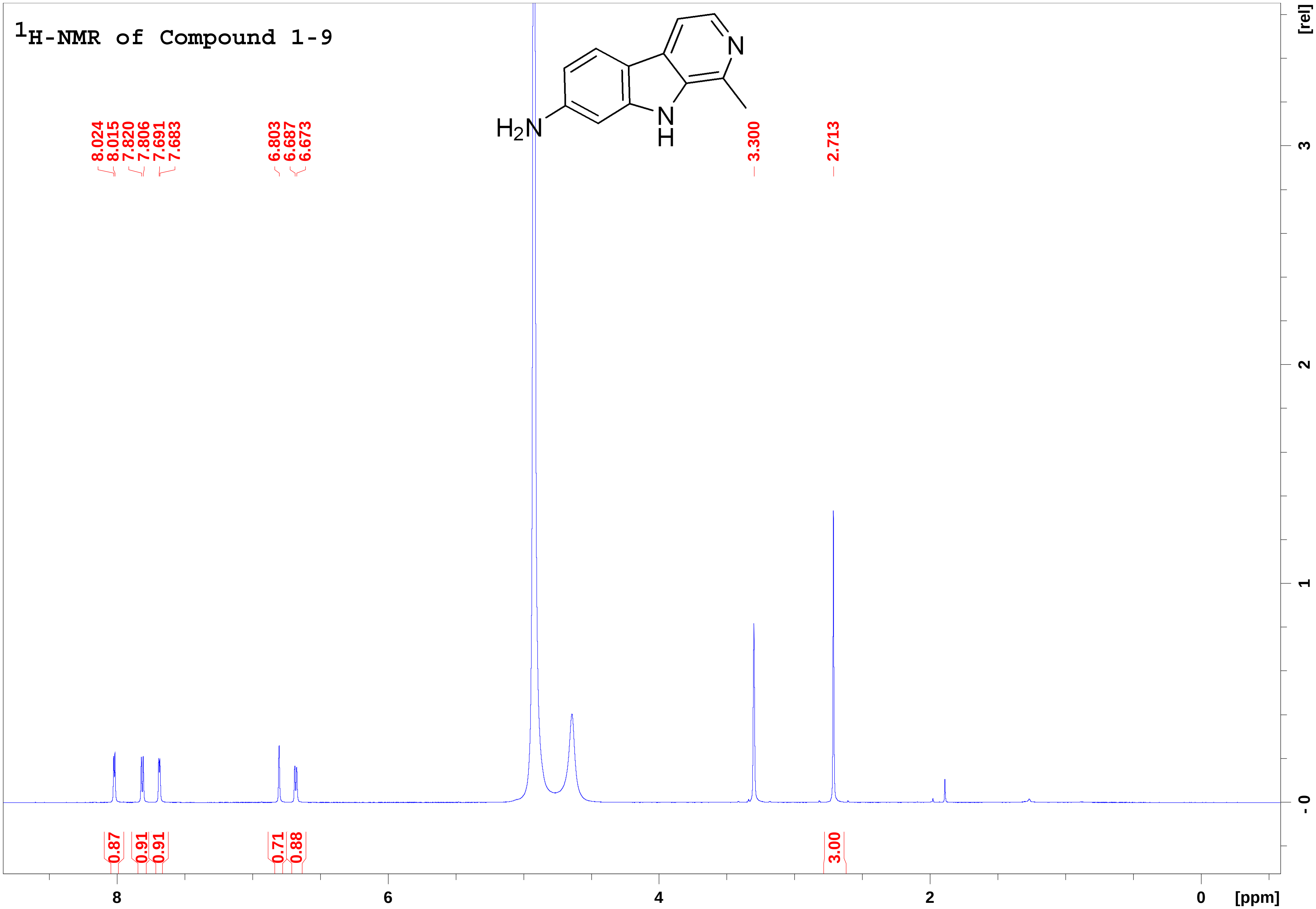

^{1}H-NMR of Compound 1-9
H_2N
N
N
H
8.024
8.015
7.820
7.806
7.691
7.683
6.803
6.687
6.673
3.300
2.713
0.87
0.91
0.91
0.71
0.88
3.00
8
6
4
2
0
[ppm]
[rel]
3
2
1
0

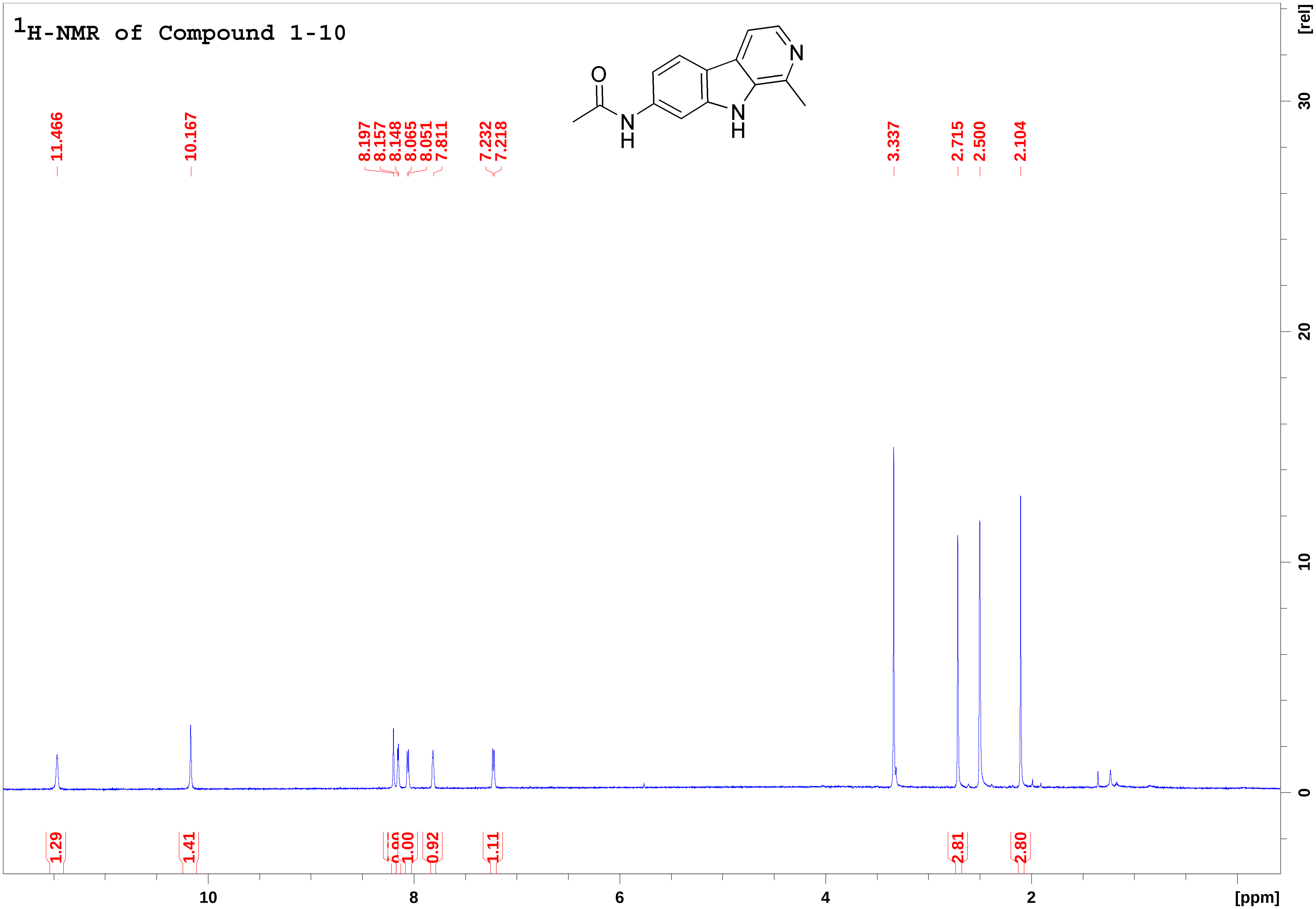

1H-NMR of Compound 1-10
O
N
H
N
H
N
11.466
10.167
8.197
8.157
8.148
8.065
8.051
7.811
7.232
7.218
3.337
2.715
2.500
2.104
1.29
1.41
1.00
0.92
1.11
2.81
2.80
[rel]
30
20
10
0
10
8
6
4
2
[ppm]

^{1}H-NMR of Compound 1-11

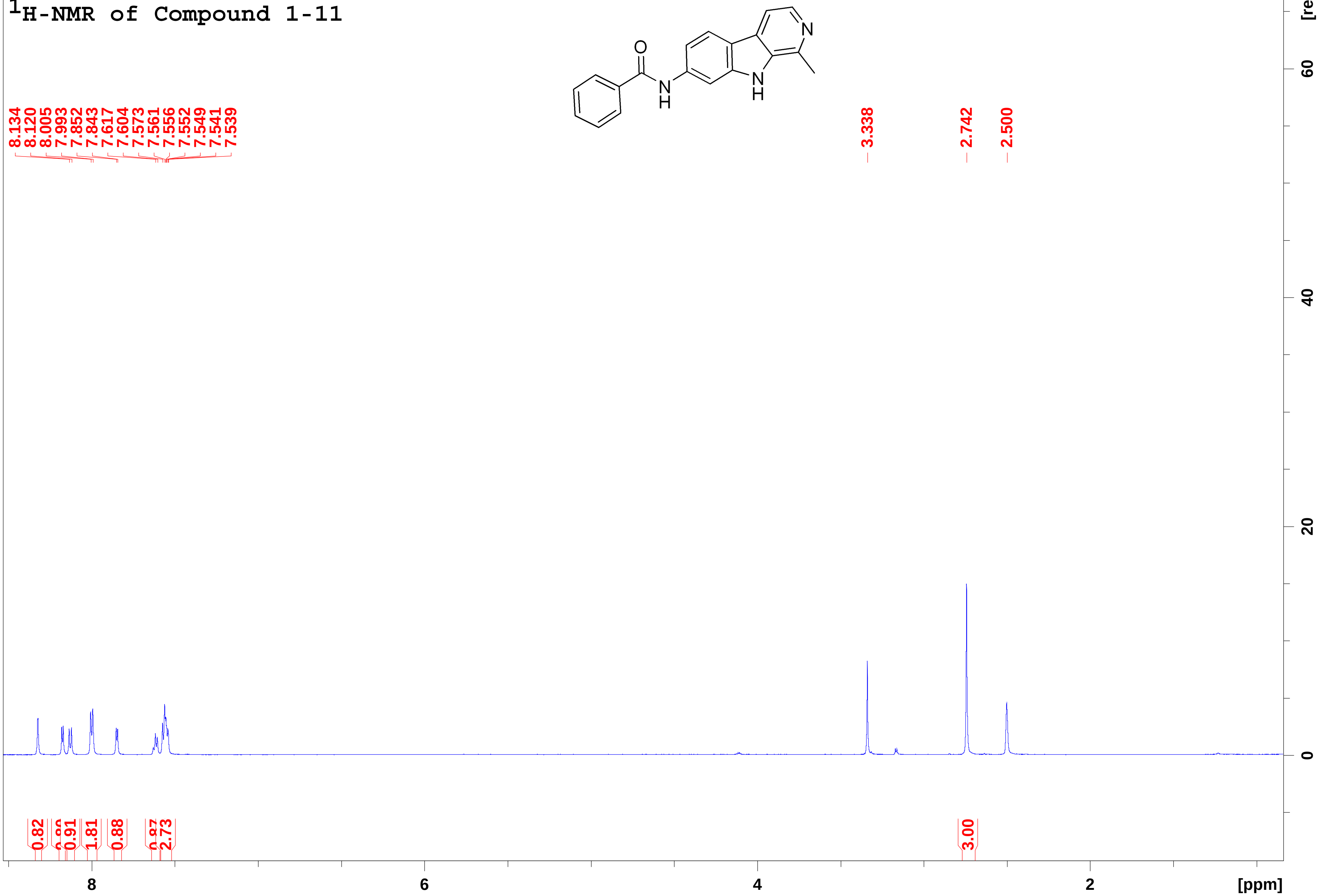

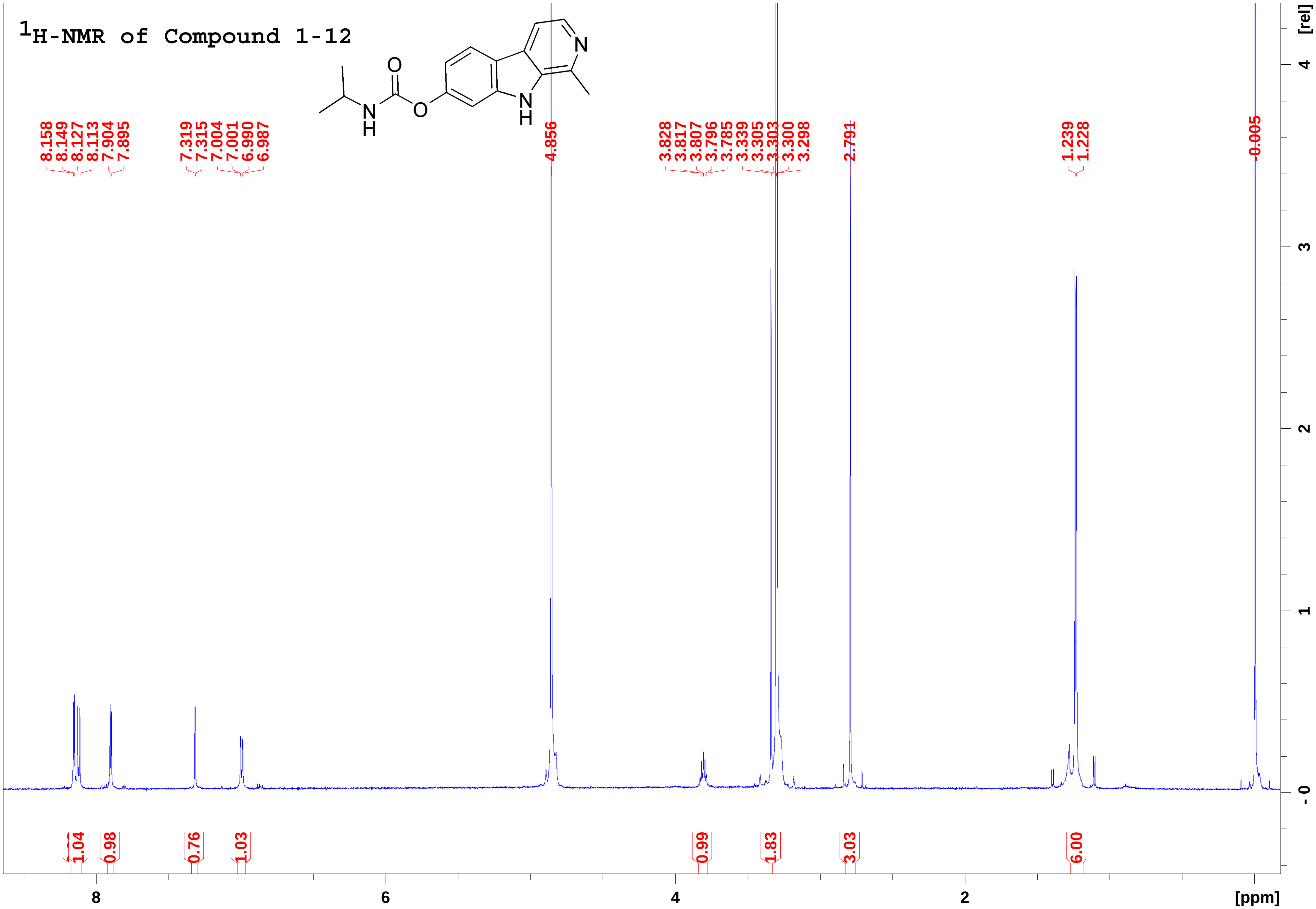

1H-NMR of Compound 1-12
O
N
H
O
N
N
H
8.158
8.149
8.127
8.113
7.904
7.895
7.319
7.315
7.004
7.001
6.990
6.987
4.856
3.828
3.817
3.807
3.796
3.785
3.339
3.305
3.303
3.300
3.298
2.791
1.239
1.228
-0.005
1.04
0.98
0.76
1.03
0.99
1.83
3.03
6.00
[rel]
4
3
2
1
0
8
6
4
2
[ppm]
